# Aerobic Exercise Training Exerts Neuroprotective Effects in Alzheimer's Disease Mice by Regulating Endoplasmic Reticulum Stress‐Autophagy Pathway‐Mediated Pyroptosis

**DOI:** 10.1111/cns.70620

**Published:** 2025-11-17

**Authors:** Yunliang Wang, Xiangyun Sun, Biao He, Shaowen Yu, Yiyou Yu

**Affiliations:** ^1^ College of Physical Education AnHui Normal University Wuhu People's Republic of China; ^2^ The Department of Geriatrics The First Affiliated Hospital of Wannan Medical College (Yijishan Hospital) Wuhu People's Republic of China; ^3^ Department of Basic Medicine Wannan Medical College Wuhu People's Republic of China; ^4^ Department of Graduate School Wannan Medical College Wuhu People's Republic of China

**Keywords:** aerobic exercise training, Alzheimer's disease, autophagy, cognitive function, endoplasmic reticulum stress, hippocampal CA1 region, PERK‐eIF2α pathway, pyroptosis

## Abstract

**Objective:**

This study probed into the neuroprotective effects of aerobic exercise training (AET) on Alzheimer's disease (AD) mice and further explored the molecular mechanisms through which AET regulates the endoplasmic reticulum stress (ERS)–autophagy pathway to mediate pyroptosis.

**Methods:**

APP/PS1 mice (AD model) underwent 8 weeks of treadmill‐based AET. In addition to the exercise regimen, mice were treated with intraperitoneal injections of an NLRP3 inflammasome activator, an autophagy inhibitor, an ERS inducer, and a PERK activator for assessing cognitive function and neuronal damage in the hippocampal CA1 region through cognitive assessments, histological analyses, and biochemical assays. BrdU/EdU labeling combined with NeuN and doublecortin immunostaining was used to evaluate AET‐stimulated neuronal proliferation and differentiation in the hippocampus.

**Results:**

AET improved cognitive function in AD mice. Following AET, neuronal damage in the hippocampal CA1 region was reduced, the number of Nissl bodies increased, and Aβ_1‐42_ and p‐Tau protein levels decreased. Mechanistically, AET alleviated NLRP3 inflammasome‐mediated pyroptosis and cognitive dysfunction in AD mice by inhibiting ERS and promoting autophagy in the hippocampal CA1 region. Activation of the NLRP3 inflammasome or inhibition of autophagy partially reversed the beneficial effects of AET on pyroptosis and cognitive dysfunction in AD mice. Moreover, AET reduced ERS by inhibiting the PERK‐eIF2α pathway, thereby enhancing autophagy, reducing pyroptosis, and improving cognitive dysfunction.

**Conclusion:**

AET reduces NLRP3 inflammasome‐mediated pyroptosis and neuronal damage in the hippocampal CA1 region of AD mice by regulating the ERS‐autophagy pathway through the inhibition of the PERK‐eIF2α pathway, thereby improving cognitive function in AD mice.

## Graphical abstract

Aerobic exercise training regulates the ERS‐autophagy pathway‐mediated cell pyroptosis by inhibiting the PERK‐elF2a pathway, and reduces the deposition of Aß and p‐Tau, thereby reducing nerve injury in the hippocampal CA1 region and improving cognitive function in AD mice.

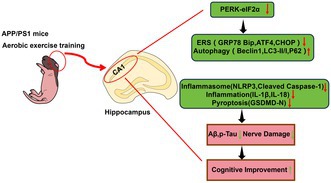

## 1 Introduction

Alzheimer's disease (AD) is a common neurodegenerative disorder characterized by cognitive impairment and memory decline, with a complex pathogenesis involving various molecular pathological changes [1]. Among the brain regions most severely affected in AD patients, the CA1 region of the hippocampus stands out as closely related to memory and spatial navigation, with its dysfunction contributing to cognitive deficits in AD [2]. The classical pathological features of AD include abnormal accumulation of amyloid‐β (Aβ)_1–42_ and hyperphosphorylation of Tau protein, leading to neuronal damage, neuroinflammation, and cell death [3]. Therefore, identifying effective interventions to slow down the progression of AD pathology and improve patients' cognitive function has become a hot research topic.

Recently, pyroptosis, a newly recognized form of programmed cell death, has been increasingly implicated in the pathogenesis of AD [4]. This process, which is dependent on NLRP3 inflammasome activation, exacerbates neuronal damage by triggering neuroinflammatory responses [5]. The activation of the NLRP3 inflammasome in AD leads to Caspase‐1 cleavage, ultimately inducing cell membrane pore formation through Gasdermin‐D (GSDMD) and initiating pyroptosis [6]. Recent evidence has shown a direct correlation between NLRP3 inflammasome activation and cognitive impairments in AD [7], suggesting that modulating NLRP3 inflammasome activation to reduce pyroptosis may offer new therapeutic targets for AD treatment.

Endoplasmic reticulum stress (ERS) plays a crucial role in the neurodegenerative process of AD, where it represents a cellular stress response to unfolded protein accumulation, activating the unfolded protein response to restore cellular homeostasis [8]. Excessive and sustained ERS may lead to neuronal apoptosis by modulating a series of key protein pathways, thereby exacerbating Aβ cytotoxicity and accelerating the progression of AD [9]. Studies have reported that treatment with the ERS inducer tunicamycin can reverse the autophagy‐promoting effect of ASPP2 shRNA, indicating that ERS suppresses autophagy [10]. Recent research further demonstrates that pharmacological downregulation of the unfolded protein response in the cortex of APP/PS1 mice enhances autophagic efficiency [11]. Therefore, modulating the interaction between ERS and autophagy may offer new strategies to slow down AD progression.

Aerobic exercise training (AET) has been demonstrated as a non‐pharmacological intervention with protective effects against various neurodegenerative diseases [12]. Studies have shown that aerobic exercise can improve cognitive impairments in AD mice through multiple molecular mechanisms. Specifically, AET can regulate neuroinflammatory responses, inhibit NLRP3 inflammasome activation to reduce pyroptosis, and suppress ERS to promote autophagy restoration [13, 14]. Previous studies in rodent models have demonstrated that 8–12 weeks of aerobic exercise is sufficient to induce improvements in AD‐related pathological markers, such as Aβ deposition and synaptic plasticity, as well as cognitive function [15, 16, 17]. For example, Dan Shi et al. have reported that an 8‐week moderate‐intensity aerobic exercise intervention significantly affects Aβ deposition, inflammatory cytokines, oxidative stress markers, neuronal damage, and cognitive function in APP/PS1 mice [16]. The exercise protocol used in our study was primarily based on this work, with minor modifications. In recent years, increasing evidence has highlighted the key role of the PERK‐eIF2α pathway in regulating ERS and autophagy [18]. However, it remains unclear whether AET can improve cognitive impairments in AD mice by modulating the ERS‐autophagy pathway to reduce pyroptosis. Thus, this study aims to elucidate the neuroprotective effects of AET in AD mice to further investigate its molecular mechanisms, focusing on the PERK‐eIF2α pathway's role in regulating the ERS‐autophagy pathway and its impact on pyroptosis in AD.

## 2 Materials and Methods

### 2.1 Experimental Animals and Grouping

A total of 150 male APP/PS‐1 double‐transgenic mice, aged 7 months, were purchased from Wannan Medical College [SYXK (Wan) 2023–005] (Wuhu, China). The mice were housed in cages at 20°C–26°C with free access to food and water, under a 12‐h light/dark cycle. All animal experiments in this study were reviewed and approved by the Animal Ethics Committee of The First Affiliated Hospital of Wannan Medical College (Yijishan Hospital).

The mice were randomized into the following seven groups, with 12 mice per group: AD group (APP/PS‐1), AET group, AET + Nig group (AET combined with 4 mg/kg NLRP3 activator Nigericin sodium salt) [19], AET + 3‐MA group (AET combined with 1.5 mg/kg autophagy inhibitor 3‐Methyladenine) [3‐MA] [20], AET + TG group (AET combined with 0.2 mg/kg ERS activator Thapsigargin) [21], AET + CCT group (AET combined with 2 mg/kg PERK activator CCT020312) [22], AET + DMSO group (AET combined with DMSO treatment). The AET and/or drug treatments were administered for 8 weeks, with drugs injected intraperitoneally once daily.

After 8 weeks, spatial learning and cognitive abilities of all mice in each group (*n* = 12) were evaluated using the Morris water maze (MWM) and step‐down avoidance (SDA) tests. Mice were then euthanized via intraperitoneal injection of 150 mg/kg sodium pentobarbital (Sigma Aldrich, St. Louis, MO, USA). Hippocampal tissue from the CA1 region of six randomly selected mice per group was fixed in 4% paraformaldehyde, dehydrated, paraffin‐embedded, and sectioned for immunohistochemistry (IHC), hematoxylin‐eosin (HE) staining, and Nissl staining. The remaining six mice per group had hippocampal CA1 tissue homogenized and frozen at −80°C for Western blot and ELISA analysis.

Nigericin sodium salt (HY‐100381), 3‐MA (HY‐19312), Thapsigargin (TG, HY‐13433), and CCT020312 (HY‐119240) were purchased from MCE (Shanghai Zhangjiang Biopharmaceutical Base, China).

### 2.2 AET

In this study, exercise training was conducted using a treadmill (Xinruan Technology Co. Ltd., Hangzhou, Zhejiang, China). The exercise protocol was a mildly modified version based on prior research, designed to simulate moderate‐intensity aerobic exercise [13, 15, 16]. Prior to the formal treadmill training, mice requiring exercise therapy underwent 1 week of an adaptive training period to familiarize themselves with treadmill operation. The adaptation schedule was as follows: Day 1: 5 m/min for 30 min; Days 3 and 4: 8 m/min for 30 min; Days 5–7: 12 m/min for 30 min, for a total of 5 days. After the end of adaptive training, the formal treadmill training protocol was implemented. Mice exercised on a zero‐inclination treadmill beginning with a 5‐min run at 5 m/min, followed by a 5‐min warm‐up at 8 m/min, then 30 min at 12 m/min as the main exercise phase, and concluding with a 5‐min recovery phase at 5 m/min. The training was performed 5 days per week for a total of 8 weeks as a formal training period. In accordance with the habits of rodents, exercise sessions commenced daily at 17:00.

### 2.3 SDA Test

The SDA test apparatus consisted of a chamber with an electrified grid floor and a circular insulated platform (4.5 cm in diameter and 4.5 cm high) placed in the center. Mice were placed in the chamber for 3 min to acclimate before the grid floor was electrified (36 V, 0.5 mA) for 5 min. Upon receiving a shock, the mice jumped onto the platform to avoid further stimulation. Learning and memory abilities were assessed by measuring the step‐down latency (duration before the animal stepped down from the platform for the first time) and the number of errors (number of times the mice jumped off the platform) within 5 min [23].

### 2.4 MWM Test

The MWM test consisted of navigation trials, spatial probe trials, and visible platform trials to assess the spatial learning and memory abilities of the mice [24]. In the water maze analysis system (Shanghai Xinruan, XR‐XM101), an opaque circular pool (120 cm in diameter, 50 cm deep) was filled with water maintained at 21°C ± 2°C. Mice underwent 4 daily trials for 5 days in the hidden platform test. On the 6th day, the platform was removed, and the mice were released from the same quadrant, with the time taken to reach the previous platform location being recorded. On the 7th and 8th days, the platform was elevated 1 cm above the water surface and relocated to another quadrant, and the number of times the mice crossed the target platform within 1 min was recorded.

### 2.5 HE Staining

Hippocampal CA1 tissues were fixed in 4% paraformaldehyde for 6 h and then embedded in paraffin. The paraffin‐embedded hippocampal tissues were sectioned into 3 μm slices, which were baked overnight at 60°C. The sections were deparaffinized twice in xylene I and xylene II (Nanjing Reagent, C0430530223), followed by hydration in ethanol at concentrations of 100%, 100%, 95%, 80%, and 70% for 5 min each. The sections were then stained with hematoxylin (Beyotime, Shanghai, China, C0107S) for 10 min, rinsed with water for 15 min to achieve bluing, and then counterstained with eosin (Beyotime, C0107M) for 30 s before being washed with double‐distilled water. Dehydration was performed using ethanol, and the sections were cleared with xylene before mounting with neutral resin (Solarbio, Beijing, China, G8590). Six random images of each section were captured under a microscope (Zeiss, Oberkochen, Germany, LSM900) to assess neuronal damage. The extent of neuronal injury in the CA1 region was evaluated based on the degree of neuronal death [25, 26]: Grade 0 (no damage), Grade 0.5 (< 10%), Grade 1.0 (10%–30%, mild damage), Grade 1.5 (30%–50%, mild to moderate damage), Grade 2.0 (50%–70%, moderate damage), Grade 2.5 (70%–90%, moderate to severe damage), and Grade 3.0 (> 90%, severe damage).

### 2.6 Nissl Staining

Paraffin‐embedded sections were deparaffinized with xylene and dehydrated with graded ethanol solutions. The sections were then stained with toluidine blue solution (Solarbio, G1220) for Nissl staining, followed by dehydration through graded ethanol, clearing with xylene, and mounting with neutral resin. The number of Nissl bodies in the CA1 region of the hippocampus was observed and quantified under a microscope (Zeiss, LSM900).

### 2.7 Western Blot Analysis

RIPA lysis buffer (Beyotime, P0039) containing protease inhibitor cocktail (Beyotime, P1006) was added to the tissue homogenate, followed by lysis on ice for 30 min. The lysates were centrifuged at 13,000 rpm for 10 min, and the supernatants were collected. Protein concentration was determined utilizing a BCA assay kit (Beyotime, P0012). Proteins were separated using 10% SDS‐PAGE and transferred to PVDF membranes (Millipore, Billerica, MA, USA, IPVH20200). The membranes were blocked with 5% non‐fat milk at room temperature for 2 h to prevent nonspecific binding and then incubated overnight at 4°C with rabbit anti‐β‐Actin (CST, Danvers, MA, USA, #4970), rabbit anti‐Aβ_1‐42_ (Abcam, Cambridge, UK, ab201060), rabbit anti‐GRP78/BIP (Proteintech, Wuhan, Hubei, China, 11587‐1‐AP), rabbit anti‐ATF‐4 (CST, 11815), rabbit anti‐CHOP (CST, 5554), rabbit anti‐Phospho‐PERK (CST, 3179), rabbit anti‐PERK (CST, 3192), rabbit anti‐Phospho‐eIF2α (CST, 3398), rabbit anti‐eIF2α (CST, 5324), rabbit anti‐Beclin1 (CST, 3495), rabbit anti‐LC3B (CST, 2775), and rabbit anti‐p62 (CST, 39749). The membranes were then incubated with horseradish peroxidase (HRP)‐conjugated goat anti‐rabbit IgG (Servicebio, Wuhan, Hubei, China, GB23303) for 1 h at room temperature, and the bands were visualized utilizing ECL reagent (Millipore, WBKLS0500). ImageProPlus6.0 software (MediaCybernetics, Silver Spring, MD, USA) was adopted to quantify the gray values of the bands, and β‐Actin served as the normalizer.

### 2.8 Elisa

Hippocampal CA1 tissues were weighed and homogenized in cold PBS at a ratio of 1:9. The homogenates were centrifuged at 3000 rpm for 10 min at 4°C, and the supernatants were harvested. Accordingly, IL‐1β and IL‐18 levels in the hippocampus were tested using the Mouse IL‐1β ELISA Kit (LIANKE BIOTECH, Hangzhou, Zhejiang, China, EK201B) and the Mouse IL‐18 ELISA Kit (LIANKE BIOTECH, EK218), respectively. Optical density was examined at 450 nm employing a microplate reader (BioTek Synergy HTX, USA).

### 2.9 IHC

The prepared paraffin sections were baked in a 45°C incubator for 3 h. After deparaffinization and hydration, IHC was done utilizing a Universal Mouse/Rabbit IHC Kit (Proteintech, PK10006). The sections were subjected to microwave antigen retrieval in citrate buffer (Proteintech, PR30001), with the solution heated to boiling and then naturally cooled for 5–10 min. The process was repeated three times. After cooling to room temperature, the sections were rinsed three times with TBS. To block endogenous peroxidase activity, 3% H_2_O_2_ was applied to the sections at room temperature for 10 min, followed by three washes with TBS. The sections were then incubated with 5% goat serum (Beyotime, C0265) at 37°C for 1 h in a humidified chamber, after which the blocking solution was removed. Primary antibodies, including anti‐NLRP3 (ThermoFisher, Waltham, MA, USA, MA5‐32255), anti‐GSDMD‐N (CST, 10137), and anti‐Caspase1 (Cleaved Asp210) (ThermoFisher, PA5‐38099), were applied to the sections for overnight incubation at 4°C in a humidified chamber. The next day, the sections were brought to room temperature and washed three times with Phosphate Buffered Saline with Tween (PBST). Biotinylated secondary antibody working solution was added to the sections for incubation at 37°C for 15 min in a humidified chamber, followed by the removal of unbound antibodies following three washes with PBST. HRP‐conjugated streptavidin working solution was then added, and the sections were incubated at 37°C for 15 min in a humidified chamber, followed by three additional washes with PBST.

### 2.10 BrdU, Doublecortin (DCX), and NeuN Staining

Mice were intraperitoneally injected with BrdU (50 mg/kg/day) for 3 consecutive days. Mice were sacrificed at 14 and 28 days after BrdU injection, and hippocampal CA1 tissues were collected for analyses. Specifically, tissues collected at 14 days were used to detect newborn immature neurons (BrdU^+^DCX^+^), while those collected at 28 days after BrdU injection were used to assess newborn mature neurons (BrdU^+^NeuN^+^). The samples were fixed in 4% paraformaldehyde at 4°C for 24 h, followed by cryosectioning. Frozen sections were cut at a thickness of 25 μM. DNA denaturation was performed by incubating the sections with 2 M hydrochloric acid at 37°C for 30 min. Subsequently, specimens were covered with 5% goat serum (Beyotime, C0265) in a humidified chamber at 37°C for 1 h. After removing the blocking solution, primary antibody working solutions against BrdU (Proteintech, 66241‐1‐Ig), NeuN (Proteintech, 26975‐1‐AP), and DCX (Proteintech, 13925‐1‐AP) were added to sections for incubation overnight at 4°C in a humid chamber. The following day, sections were washed three times with PBST and then incubated for 2 h with the Alexa Fluor 594‐conjugated donkey anti‐mouse IgG (H + L) secondary antibody (ThermoFisher, R37115). Sections were washed three times with PBST to remove unbound reagents. Thereafter, the sections were incubated with the HRP‐labeled streptavidin working solution in a humidified chamber at 37°C for 15 min, followed by three PBST washes.

### 2.11 Statistical Analysis

All data were processed employing GraphPad 9.5 software (San Diego, CA, USA). All data were subjected to the Shapiro–Wilk normality test. Measurement data of normal distribution were tested by the parameter test method, with the independent sample *t* test used to compare the data between two groups, and one‐way analysis of variance (ANOVA) analysis used to compare the data among multiple groups, followed by Tukey's multiple comparison test. The Mann–Whitney U test was used to analyze the variables that did not conform to the normal distribution. All tests were two‐sided, and differences were deemed statistically significant at *p* < 0.05.

## 3 Results

### 3.1 AET Inhibits NLRP3 Inflammasome Activation and Reduces Pyroptosis in the Hippocampal CA1 Region to Improve Cognitive Dysfunction in AD Mice

**FIGURE 1:**
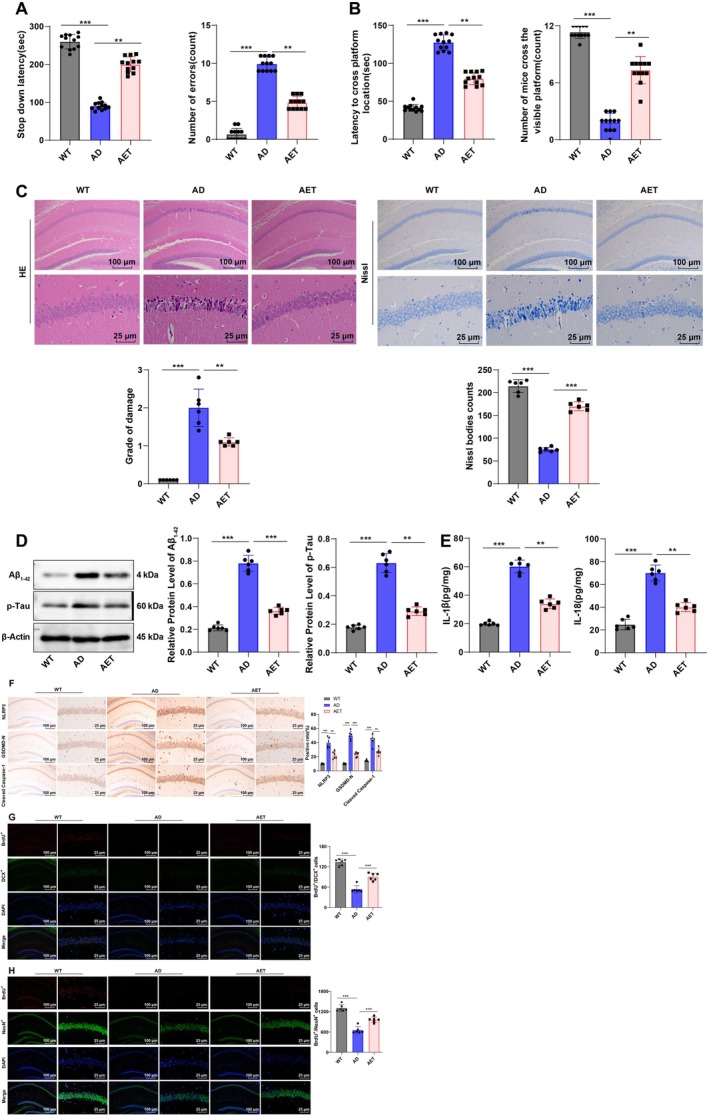
Effects of AET on pyroptosis and cognitive function in the hippocampal CA1 region of APP/PS1 mice. (A) Step‐down latency and number of errors in the SDA test, *N* = 12. (B) Time to reach the target platform and the number of crossings in the MWM test, *N* = 12. (C) HE staining and Nissl staining were adopted to observe neuronal damage in the hippocampal CA1 region, *N* = 6. (D) Western blot analysis of Aβ_1‐42_ and p‐Tau expression in the hippocampus, *N* = 6. (E) ELISA analysis of IL‐1β and IL‐18 levels, *N* = 6. (F) IHC detection of NLRP3, GSDMD‐N, and Cleaved Caspase‐1 expression in the hippocampus, *N* = 6. (G) Immunostaining to detect BrdU^+^ and DCX^+^ cells in AD mice after 14 days of BrdU treatment, *N* = 6. (H) Immunostaining to detect BrdU^+^ and NeuN^+^ cells in AD mice after 28 days of BrdU treatment, *N* = 6. The Shapiro–Wilk test showed that data in panel A did not conform to the normal distribution, and the Mann–Whitney U test was used for comparisons between groups. Other data were tested by the Shapiro–Wilk test to be in line with the normal distribution, which were presented as mean ± standard deviation, with one‐way ANOVA used for inter‐group comparisons, and the Tukey's multiple comparison test used afterwards. **p < 0.01, ***p < 0.001.

To investigate the effects of AET on cognitive dysfunction in APP/PS1 mice, we performed the SDA and MWM tests (Figure 1A,B). In the SDA test, compared to WT mice, AD mice showed a decreased step‐down latency and an increased number of errors (Figure 1A, both *p* < 0.001). In the MWM test, AD mice exhibited a longer time to find the target quadrant and a lower frequency of platform crossings than WT mice (Figure 1B, both *p* < 0.001); however, AET treatment significantly improved cognitive deficits in APP/PS1 mice (Figure 1A,B, all *p* < 0.01).

Histological analysis using HE and Nissl staining further examined the effects of AET on neuronal injury in the hippocampal CA1 region of APP/PS1 mice. Compared to WT mice, AD mice presented notably enhanced neuronal damage and reduced Nissl body numbers in the CA1 region, whereas AET alleviated histopathological changes and neuronal loss in the hippocampal CA1 region of APP/PS1 mice (Figure 1C, all *p* < 0.01).

Western blot analysis demonstrated that AD mice showed higher Aβ_1‐42_ and p‐Tau expression than WT mice, while AET evidently diminished Aβ_1‐42_ and p‐Tau accumulation in AD mice (Figure 1D, both *p* < 0.01). Previous evidence has identified the critical role of pyroptosis in AD pathology [27]. ELISA revealed that mice in the AD group displayed elevated levels of inflammatory cytokines IL‐1β and IL‐18 relative to those in the WT group; yet, AET diminished the levels of IL‐1β and IL‐18 in the hippocampal CA1 region of AD mice (Figure 1E, both *p* < 0.01). IHC demonstrated that compared with WT mice, NLRP3, GSDMD‐N, and Cleaved Caspase‐1 expression in the hippocampal CA1 region of AD mice was significantly increased, while AET led to reductions in the expression of NLRP3, Cleaved Caspase‐1, and GSDMD‐N, which are associated with inflammasome activation and pyroptosis, in the hippocampal CA1 region (Figure 1F, all *p* < 0.01). The number of BrdU^+^/DCX^+^ and BrdU^+^/NeuN^+^ cells can be used to detect the number of newborn immature neurons and newborn mature neurons, respectively [28]. The results showed that BrdU^+^/DCX^+^ and BrdU^+^/NeuN^+^ in the hippocampal CA1 region of AD mice were significantly decreased compared to WT mice, but were significantly increased upon AET intervention (Figure 1G,H, all *p* < 0.001). Conclusively, AET inhibits NLRP3 inflammasome activation to reduce pyroptosis in the hippocampal CA1 region and stimulate neuronal proliferation and differentiation in the hippocampus, thus improving cognitive dysfunction in AD mice.

### 3.2 Activation of NLRP3 Inflammasome Partially Reverses the Protective Effects of AET on Pyroptosis and Cognitive Dysfunction in AD Mice

**FIGURE 2:**
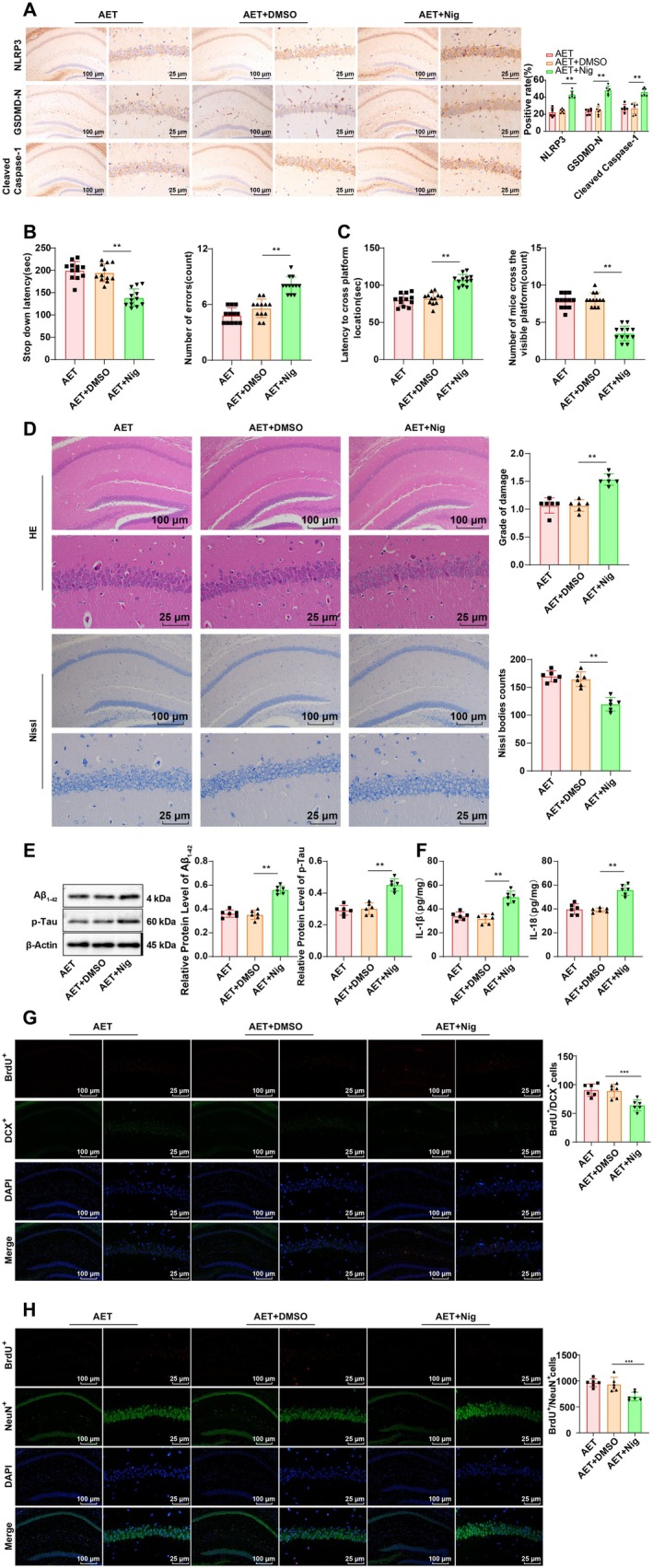
Effects of NLRP3 inflammasome activation on pyroptosis and cognitive function in the hippocampal CA1 region of APP/PS1 mice following AET. (A) IHC detection of NLRP3, Cleaved Caspase‐1, and GSDMD‐N expression in the hippocampus, *N* = 6. (B) Step‐down latency and number of errors in the SDA test, *N* = 12. (C) Time to reach the target platform and the number of crossings in the MWM test, *N* = 12. (D) HE staining and Nissl staining were adopted to observe neuronal damage in the hippocampal CA1 region, *N* = 6. (E) Western blot analysis of Aβ_1‐42_ and p‐Tau expression in the hippocampus, *N* = 6. (F) ELISA analysis of IL‐1β and IL‐18 expression in the hippocampus, *N* = 6. (G) Detection of BrdU^+^ and DCX^+^ cells in AD mice after 14 days of BrdU treatment using immunostaining, *N* = 6. (H) Detection of BrdU^+^ and NeuN^+^ cells in AD mice after 28 days of BrdU treatment using immunostaining, *N* = 6. Data in panels B and D were tested by the Shapiro–Wilk test to be not conform to the normal distribution, and the Mann–Whitney U test was used for analyses. Other data were tested by the Shapiro–Wilk test to be in line with the normal distribution, which were presented as mean ± standard deviation. Comparisons between groups were performed using one‐way ANOVA followed by Tukey's multiple comparisons test. **p < 0.01, ***p < 0.001.

We then focused on whether the reduction in neuronal pyroptosis in the hippocampal CA1 region is linked to the improvement in cognitive function in AD mice following AET. Mice were treated with the NLRP3 inflammasome activator Nigericin sodium salt in addition to AET. It was evident that compared to the AET + DMSO group, AET + Nig‐treated mice exhibited notably elevated expression of NLRP3, GSDMD‐N, and Cleaved Caspase‐1 in the hippocampal CA1 region (Figure 2A, all *p* < 0.01). In the SDA test, AET + Nig‐treated mice had a shorter step‐down latency and an increased number of errors (Figure 2B, both *p* < 0.01). In the MWM test, AET + Nig‐treated mice took a longer time to find the target quadrant and exhibited a reduced frequency of platform crossings (Figure 2C, both *p* < 0.01). Furthermore, these mice displayed increased neuronal damage in the hippocampal CA1 region, decreased Nissl body numbers (Figure 2D, all *p* < 0.01), diminished BrdU^+^/DCX^+^ and BrdU^+^/NeuN^+^ (Figure 2G,H, all *p* < 0.001), and increased accumulation of Aβ_1‐42_ and p‐Tau (Figure 2E, all *p* < 0.01). Additionally, the levels of IL‐1β and IL‐18 were elevated in the hippocampal CA1 region of AET + Nig‐treated mice (Figure 2F, both *p* < 0.01). All in all, activation of the NLRP3 inflammasome can partially reverse the protective effects of AET on neuronal pyroptosis and cognitive dysfunction in AD mice.

### 3.3 AET Inhibits ERS and Increases Autophagy Levels in the Hippocampal CA1 Region of AD Mice

**FIGURE 3:**
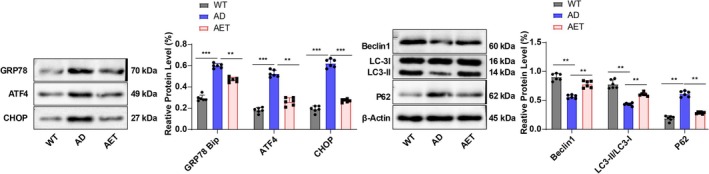
Effects of AET on ERS and autophagy levels in the hippocampal CA1 region of AD mice. Western blot analysis of GRP78/Bip, ATF4, CHOP, Beclin1, LC3‐I/II, and P62 protein level, *N* = 6. All data subjected to the Shapiro–Wilk test were confirmed to be in accordance with the normal distribution. Data were presented as mean ± standard deviation. Comparisons between groups were performed using one‐way ANOVA, followed by Tukey's multiple comparisons test. **p < 0.01, ***p < 0.001.

Autophagy is known to negatively regulate NLRP3 inflammasome activation, thereby inhibiting pyroptosis, while dysfunction in autophagy exacerbates AD pathology [29]. Research indicates that the ERS inducer treatment counteracts the autophagy facilitated by ASPP2 shRNA treatment, indicating that ERS suppresses autophagy [10]. To explore whether AET exerts its effects through the ERS‐autophagy pathway, we further examined the expression of ERS‐related genes GRP78/Bip, ATF4, and CHOP, as well as autophagy‐related genes Beclin1, LC3‐II, LC3‐I, and P62 in the hippocampal CA1 region of AD mice by Western blot. The results demonstrated that the protein level of ERS‐related genes was notably elevated in the AD group versus the WT group, whereas AET treatment reversed this phenomenon (Figure 3, all *p* < 0.01), highlighting that AET alleviated excessive ERS in the hippocampal CA1 region of AD mice. In addition, compared with the WT group, mice in the AD group had significantly decreased autophagy initiation markers Beclin1 and the LC3‐II/I ratio in the hippocampal CA1 region, as well as raised P62 protein levels; AET increased Beclin1 and the LC3‐II/I ratio and decreased the P62 protein level (Figure 3, all *p* < 0.01). These findings uncover that AET can inhibit ERS and enhance autophagy levels in the hippocampal CA1 region of AD mice.

### 3.4 Inhibition of Autophagy Partially Reverses the Regulatory Effects of AET in AD Mice

**FIGURE 4:**
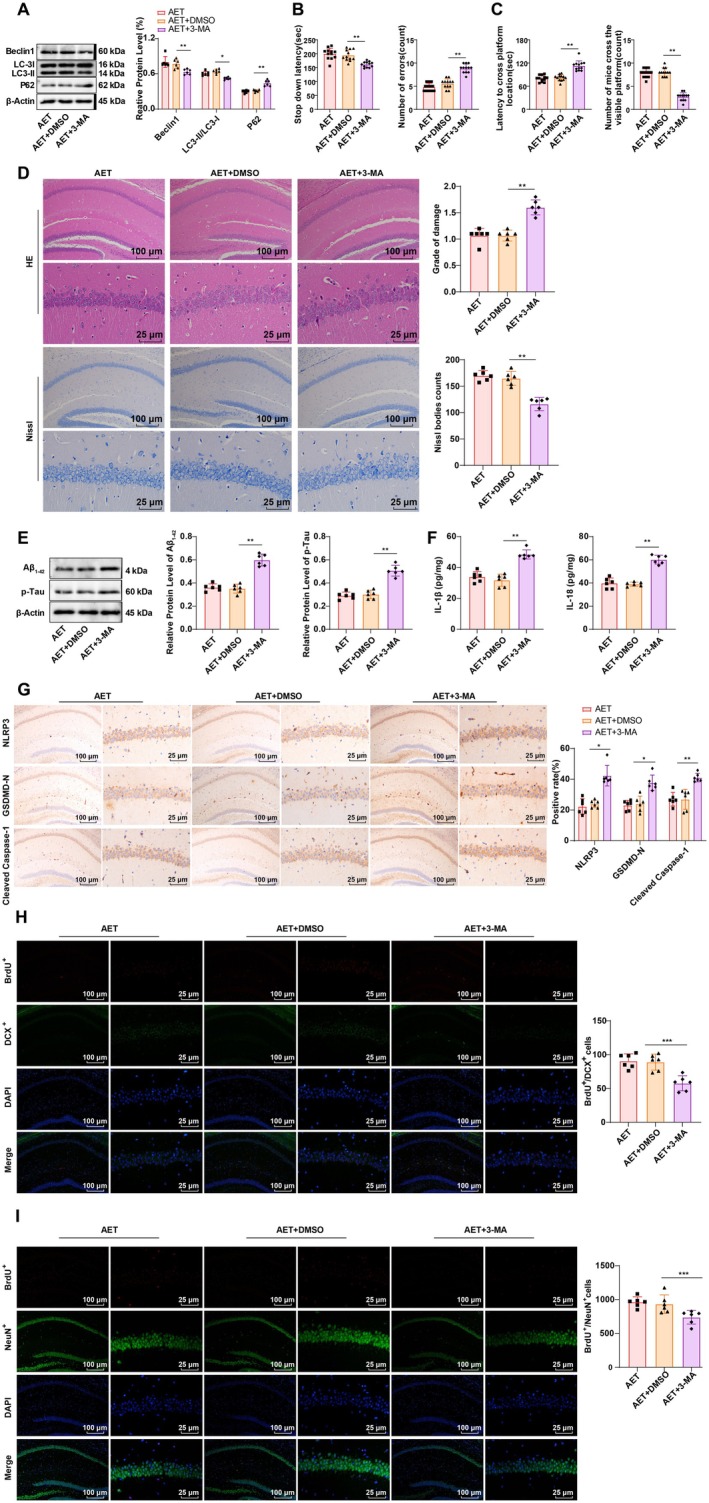
Effects of autophagy inhibition on pyroptosis and cognitive function in the hippocampal CA1 region of APP/PS1 mice following AET. (A, E) Western blot analysis of Beclin1, LC3‐I, LC3‐II, P62, Aβ_1‐42_, and p‐Tau expression in the hippocampal CA1 region, *N* = 6. (B) Step‐down latency and number of errors in the SDA test, *N* = 12. (C) Time to reach the target platform and the number of crossings in the MWM test, *N* = 12. (D) HE staining and Nissl staining were performed to observe neuronal damage in the hippocampal CA1 region, *N* = 6. (F) ELISA analysis of IL‐1β and IL‐18 levels in the hippocampus, *N* = 6. (G) IHC detection of NLRP3, GSDMD‐N, and Cleaved Caspase‐1 expression in the hippocampal CA1 region, *N* = 6. (H) Detection of BrdU^+^ and DCX^+^ cells in AD mice after 14 days of BrdU treatment using immunostaining, *N* = 6. (I) Detection of BrdU^+^ and NeuN^+^ cells in AD mice after 28 days of BrdU treatment using immunostaining, *N* = 6. Data in panels B and D were confirmed by the Shapiro–Wilk test to be not conform to the normal distribution, and the Mann–Whitney U test was used for analyses. Other data were confirmed by the Shapiro–Wilk test to be in line with the normal distribution. Data were presented as mean ± standard deviation. Comparisons between groups were performed using one‐way ANOVA followed by Tukey's multiple comparisons test. *p < 0.05, **p < 0.01, ***p < 0.001.

Our next focus was the significance of autophagy activation in the hippocampus of AD mice on the therapeutic effects of AET. Mice following AET were treated with the autophagy inhibitor 3‐MA [20]. Protein expression determination identified that 3‐MA treatment inhibited the autophagy activation induced by AET in the hippocampus of AD mice (Figure 4A, all *p* < 0.01). Moreover, compared to the AET + DMSO group, the AET + 3‐MA group exhibited a partial reversal of the improvement in cognitive dysfunction observed with AET (Figure 4B,C, both *p* < 0.01). Histologically, neuronal damage in the hippocampal CA1 region increased, and the number of Nissl bodies decreased in the AET + 3‐MA group (Figure 4D, both *p* < 0.01), and BrdU^+^/DCX^+^ and BrdU^+^/NeuN^+^ were also significantly diminished in the AET + 3‐MA group (Figure 4H,I, all *p* < 0.001). Additionally, the expression of Aβ_1‐42_ and p‐Tau was elevated in the hippocampal CA1 region (Figure 4E), as were the levels of IL‐1β and IL‐18 (Figure 4F, all *p* < 0.01). Furthermore, 3‐MA treatment led to a notable increase in the expression of NLRP3, Cleaved Caspase‐1, and GSDMD‐N (Figure 4G, all *p* < 0.05). These findings reveal that inhibiting autophagy can partially reverse the effects of AET on suppressing hippocampal CA1 pyroptosis and improving cognitive function in AD mice.

### 3.5 AET Improves Pyroptosis and Cognitive Dysfunction in AD Mice by Inhibiting ERS and Activating Autophagy in the Hippocampal CA1 Region

**FIGURE 5:**
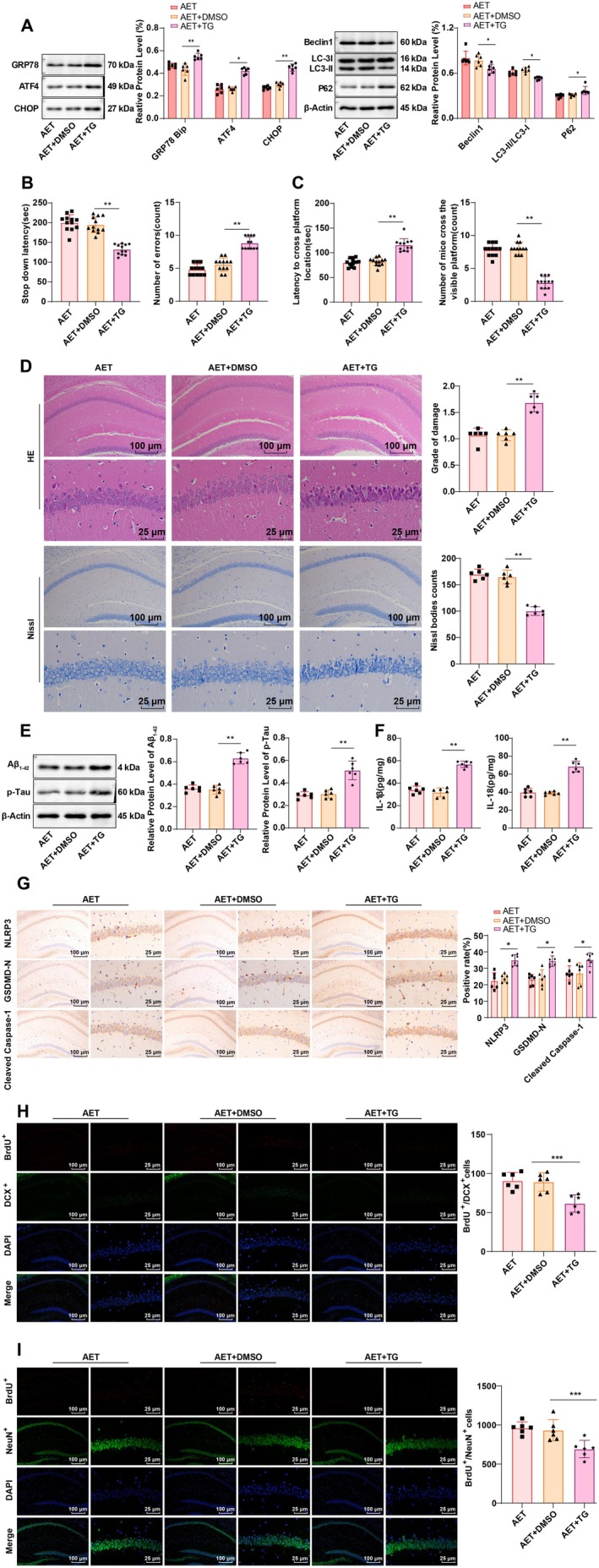
Effects of ERS activation on autophagy, pyroptosis, and cognitive function in the hippocampal CA1 region of APP/PS1 mice following AET. (A, E) Western blot analysis of GRP78/Bip, ATF4, CHOP, Beclin1, LC3‐I/II, P62, Aβ_1‐42_, and p‐Tau expression in the hippocampus, *N* = 6. (B) Step‐down latency and number of errors in the SDA test, *N* = 12. (C) Time to reach the target platform and the number of crossings in the MWM test, *N* = 12. (D) HE staining and Nissl staining were conducted to observe neuronal damage in the hippocampal CA1 region, *N* = 6. (F) ELISA analysis of IL‐1β and IL‐18 levels in the hippocampal CA1 region, *N* = 6. (G) IHC detection of NLRP3, GSDMD‐N, and Cleaved Caspase‐1 expression in the hippocampal CA1 region, *N* = 6. Data in panels B and D were confirmed by the Shapiro–Wilk test to be not conform to the normal distribution, and the Mann–Whitney U test was used for analyses. Other data were confirmed by the Shapiro–Wilk test to be in line with the normal distribution. Data were presented as mean ± standard deviation. Comparisons between groups were performed using one‐way ANOVA followed by Tukey's multiple comparisons test. *p < 0.05, **p < 0.01, ***p < 0.001.

Since ERS can induce autophagy, we further investigated the molecular mechanisms underlying the relationship between ERS and autophagy in the hippocampal CA1 region of AD mice following AET. To this end, mice were treated with the ERS activator TG [21] in combination with AET. The results demonstrated that, compared to the AET + DMSO group, the AET + TG group exhibited an increased level of GRP78/Bip, ATF4, and CHOP, while the level of Beclin1 and the LC3‐II/I ratio decreased, and P62 expression elevated (Figure 5A, all *p* < 0.05), and the promoting effect of AET on autophagy was partially reversed by TG treatment. Furthermore, TG treatment partially reversed the improvement in cognitive dysfunction induced by AET, as shown by worsened performance in behavioral tests (Figure 5B,C, both *p* < 0.01), increased neuronal damage in the hippocampal CA1 region, decreased the number of Nissl bodies (Figure 5D, all *p* < 0.01), decreased BrdU^+^/DCX^+^ and BrdU^+^/NeuN^+^ (Figure 5H,I, all *p* < 0.001), elevated expression of Aβ_1‐42_ and p‐Tau (Figure 5E, both *p* < 0.01), along with the levels of IL‐1β and IL‐18 (Figure 5F, both *p* < 0.01), and expression of NLRP3, Cleaved Caspase‐1, and GSDMD‐N (Figure 5G, all *p* < 0.05). These findings unfold that activation of ERS reduces autophagy in the hippocampal CA1 region and partially reverses the protective effects of AET on neuronal pyroptosis and cognitive dysfunction in AD mice.

### 3.6 AET Improves Cognitive Dysfunction in AD Mice by Regulating the ERS‐Autophagy Pathway Through Inhibition of the PERK‐eIF2α Pathway

**FIGURE 6:**
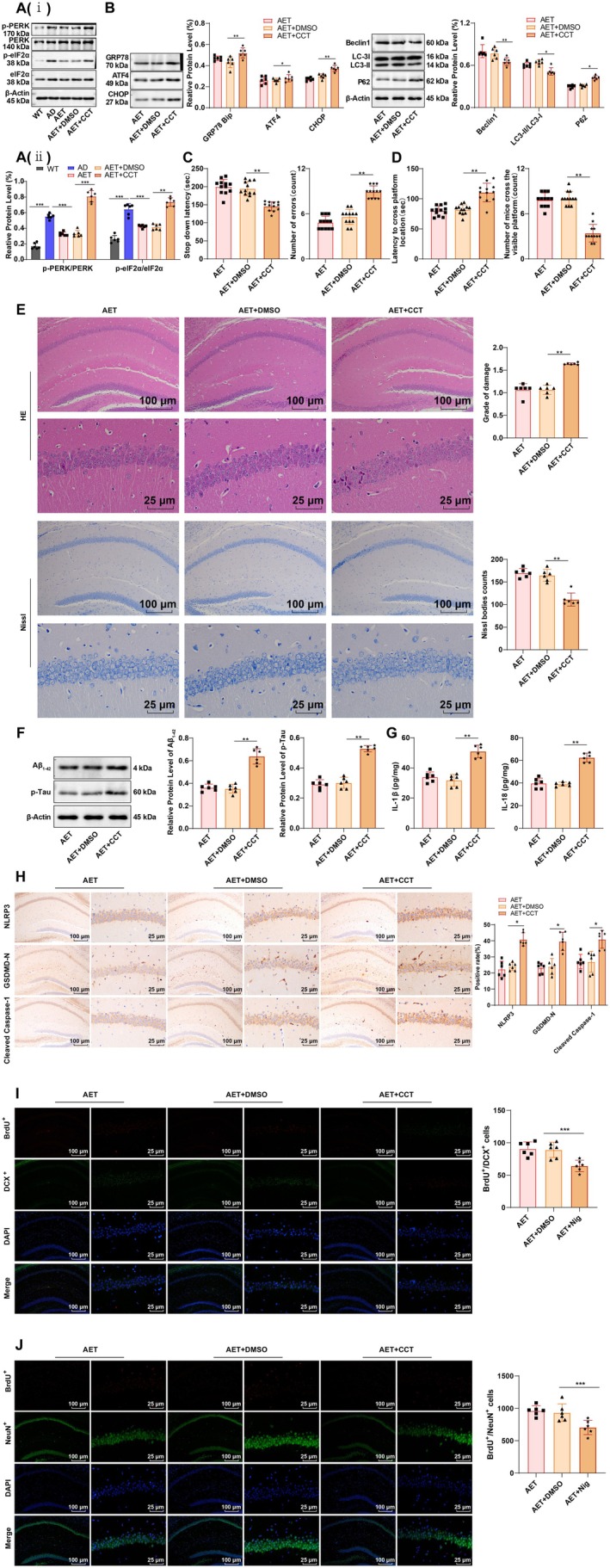
Effects of PERK activation on autophagy, pyroptosis, and cognitive function in the hippocampal CA1 region of APP/PS1 mice following AET. (A, B, F) Western blot analysis of p‐PERK, PERK, p‐eIF2α, eIF2α, GRP78/Bip, ATF4, CHOP, Beclin1, LC3‐II/I, P62, Aβ_1‐42_, and p‐Tau expression. (C) Step‐down latency and number of errors in the SDA test. (D) Time to reach the target platform and the number of crossings in the MWM test. (E) HE staining and Nissl staining to observe neuronal damage in the hippocampal CA1 region. (G) ELISA analysis of IL‐1β and IL‐18 levels in the hippocampus. (H) IHC detection of NLRP3, GSDMD‐N, and Cleaved Caspase‐1 expression in the hippocampus. (I) Detection of BrdU^+^ and DCX^+^ cells in AD mice after 14 days of BrdU treatment using immunostaining, *N* = 6. (J) Detection of BrdU^+^ and NeuN^+^ cells in AD mice after 28 days of BrdU treatment using immunostaining, *N* = 6. Data in panels B and D were confirmed by the Shapiro–Wilk test to be not conform to the normal distribution, and the Mann–Whitney U test was used for analyses. Other data were confirmed by the Shapiro–Wilk test to be in line with the normal distribution. Data were presented as mean ± standard deviation. Comparisons between groups were performed using one‐way ANOVA followed by Tukey's multiple comparisons test. *p < 0.05, **p < 0.01, ***p < 0.001.

Previous studies have documented excessive activation of the PERK‐eIF2α pathway in the brains of APP/PS1 mice, which plays a crucial role in alleviating ERS and promoting autophagy [18, 30]. To further investigate the role of the PERK‐eIF2α pathway in AET, we treated AET mice with the PERK activator CCT020312. Western blot analysis revealed that ratios of p‐PERK/PERK and p‐eIF2α/eIF2α in the AD group were significantly increased relative to those in the WT group, while the AET group had lower ratios of p‐PERK/PERK and p‐eIF2α/eIF2α than the AD group. (Figure 6A, both *p* < 0.001). However, compared to the AET + DMSO group, the AET + CCT group showed increased p‐PERK/PERK and p‐eIF2α/eIF2α ratios (Figure 6A, both *p* < 0.01). In addition, four ERS‐related genes were upregulated, while Beclin1 and the LC3‐II/I ratio were downregulated, and P62 expression was elevated in the AET + CCT group compared to the AET + DMSO group (Figure 6B, all *p* < 0.01). Behavioral tests showed that the activation of the PERK‐eIF2α pathway partially reversed the cognitive improvements induced by AET (Figure 6C,D, both *p* < 0.01). Neuronal damage in the hippocampal CA1 region increased, the number of Nissl bodies decreased significantly (Figure 6E, both *p* < 0.01), and BrdU^+^/DCX^+^ and BrdU^+^/NeuN^+^ were significantly lessened (Figure 6I,J, all *p* < 0.001). The expression of Aβ_1‐42_ and p‐Tau in the hippocampal CA1 region increased (Figure 6F, both *p* < 0.01), as did the levels of IL‐1β, IL‐18 (Figure 6G, both *p* < 0.01), NLRP3, Cleaved Caspase‐1, and GSDMD‐N (Figure 6H, all *p* < 0.05). Thus, activation of the PERK‐eIF2α pathway can exacerbate ERS and suppress autophagy, thus partially reversing the protective effects of AET on neuronal pyroptosis and cognitive function in AD mice.

## 4 Discussion

In recent years, evidence has confirmed that exercise interventions provide multiple benefits to patients with AD [31, 32]. Our current study here demonstrated that AET reduces NLRP3 inflammasome‐mediated pyroptosis and neuronal damage in the hippocampal CA1 region of AD mice. This occurs through the regulation of the ERS‐autophagy pathway via inhibition of the PERK‐eIF2α pathway, thereby improving cognitive function in AD mice. The present findings offer a promising avenue for future research and clinical strategies for managing AD.

AD is the predominant form of dementia, characterized by a gradual decline in episodic memory and cognitive abilities. This decline is often followed by impairments in language and visuospatial skills [33]. In AD, the pathology is marked by the buildup of extracellular amyloid plaques containing abnormal beta‐amyloid proteins, as well as intracellular neurofibrillary tangles formed by misfolded tau proteins within the brain [34]. Current clinical studies have shown that in patients diagnosed with AD, especially at early stages of the disease, AET alone or combined with cognitive stimulation can lead to improvements in certain aspects of brain function [35, 36]. In humans, AET has been reported to reduce the release of pro‐inflammatory cytokines in individuals with mild cognitive impairment or dementia [37], which is consistent with our findings. Moreover, recent research has investigated exercise‐induced hormone irisin levels in the cerebrospinal fluid and plasma of AD patients, examining its correlation with disease biomarkers to further elucidate the mechanisms of exercise therapy [38]. Our study here validated that after AET, cognitive function remarkably improved in AD mice, with reduced neuronal damage in the hippocampal CA1 region, increased Nissl bodies, and significant reductions in the level of Aβ_1‐42_ and p‐Tau proteins. Research indicates that engaging in regular exercise can enhance daily functioning for older adults and slow down cognitive decline in individuals diagnosed with AD [31]. More specifically, existing evidence has documented that aerobic exercise has a moderate beneficial impact on neurocognitive function in those experiencing AD, including enhancements in attention, executive function, and memory [32, 39, 40].

The importance of the NLRP3 inflammasome in AD pathology has gained significant attention in recent years. Studies have shown that NLRP3 activation is closely associated with neuroinflammation and pyroptosis, playing a crucial pathogenic role in AD [41, 42, 43]. The results of this study demonstrated that AET notably inhibited the expression of NLRP3, cleaved Caspase‐1, and GSDMD‐N, indicating that AET improves cognitive function in AD mice by reducing NLRP3‐mediated pyroptosis. Importantly, other studies have also demonstrated that inhibiting the NLRP3 inflammasome can reduce AD pathology progression and improve cognitive impairments [44, 45, 46]. Supporting our findings, recent evidence has also validated that aerobic exercise is capable of alleviating pyroptosis‐related diseases (including AD) by repressing the NLRP3 inflammasome [14].

ERS is a critical factor in AD pathology, exacerbating neuronal damage and death to promote AD progression [8]. In this study, AET was proved to suppress ERS to activate autophagy in the hippocampal CA1 region of AD mice. Autophagy possesses great potential in maintaining neuronal homeostasis and clearing toxic substances such as Aβ and Tau proteins [47]. Additionally, autophagy also shares a negative correlation with NLRP3 inflammasome activation, subsequently inhibiting pyroptosis [48]. Dysfunction in autophagic processes can worsen AD pathology [49]. Research indicates that reducing ERS can reverse the inhibition of autophagy‐related protein expression in the brains of APP/PS1 mice, resulting in decreased Aβ accumulation [50]. Supporting this, accumulating evidence has confirmed the suppressive role of exercise in ERS [51, 52]. Thus, our results further indicate that inhibiting ERS is an important mechanism by which AET exerts neuroprotective effects in AD.

Furthermore, this study further demonstrated that AET improved cognitive dysfunction in AD mice by modulating the ERS‐autophagy pathway through blockage of the PERK‐eIF2α pathway. Studies have documented the over activation of the PERK‐eIF2α pathway in the brains of APP/PS1 mice, which is critical in mitigating ERS and initiating autophagy [18, 30]. AET notably decreased the p‐PERK/PERK and p‐eIF2α/eIF2α ratios, while treatment with a PERK activator restored these ratios, exacerbating ERS and suppressing autophagy. These results highlighted the important regulatory role of the PERK‐eIF2α pathway in AET‐mediated modulation of the ERS‐autophagy pathway. Other research also indicates that inhibiting the PERK‐eIF2α pathway can alleviate neuronal damage and improve cognitive function in AD [18, 53]. As recently proved, exercise can effectively diminish the expression of PERK/eIF2α/ATF4 pathways, therefore repressing ERS and apoptosis [54]. At the same time, our study also proved that AET treatment inhibited ERS and increased the level of autophagy in the hippocampus of mice, and we verified the regulatory effect of ERS on autophagy by activating ERS to inhibit autophagy. This is consistent with previous research reporting that spermidine can significantly reduce inflammation and collagen deposition in mice by inducing autophagy and inhibiting ERS, thereby reducing pulmonary fibrosis in mice [55]. These results further reinforce the neuroprotective role of AET in AD through the modulation of these pathways.

In conclusion, this study demonstrated that AET had neuroprotective effects on mice with AD by modulating the ERS‐autophagy pathway via the PERK‐eIF2α pathway. AET not only enhanced cognitive function and decreased neuronal damage in the hippocampal CA1 region by reducing NLRP3 inflammasome‐mediated pyroptosis, but also offered potential therapeutic benefits for AD. In terms of theoretical mechanistic innovation, this study clearly proposes the “ERS‐autophagy‐pyroptosis” cascade pathway (PERK–eIF2α → LC3‐II → NLRP3), providing a novel framework for understanding the interaction between cellular stress and inflammation in AD. This mechanistic insight may also extend to the study of other neurodegenerative disorders such as Parkinson's disease [56]. From a clinical translational perspective, this discovery offers critical targets for the development of personalized exercise program design in AD patients, particularly in early stages; for instance, exercise intensity may need to reach the PERK inhibition threshold [8]. Furthermore, based on the regulatory axis of ERS‐autophagy‐pyroptosis, AET may synergize with existing pharmacological agents, such as the autophagy inducer rapamycin or the NLRP3 inhibitor MCC950. Recent clinical trials have begun to explore such a combined effect of exercise and drugs, and this study provides molecular‐level guidance for optimizing those therapeutic strategies [57, 58].

However, this study still has the following limitations. (1) This study has evaluated the short‐term effects of AET (8 weeks) on AD mice, but it is not clear whether these effects persist after exercise cessation. Given that AD is a progressive disease, assessing the long‐term effects of AET is critical to determine its therapeutic potential; (2) Referring to previous studies [13, 15], male mice were selected for this study, and the effect of sex differences on the neuroprotective effect of AET was not systematically evaluated; (3) Only one AD mouse model (APP/PS1 double‐transgenic mice) was selected for the study. It is unclear whether these results are applicable to other AD models; and (4) Evidence suggests that electrical stimulation therapy can exert positive effects on motor behaviors and biochemical changes [59]. Electrical stimulation therapy has been found to effectively slow the AD progression down in specific neurophysiological domains and improve cognitive performance in affected individuals [60]. Nevertheless, this study only investigated the effect of AET, and did not explore the effect of combining it with other AD treatment methods. In future studies, we will further clarify the combination therapy of the best combination regimen (such as exercise type and electrical stimulation mode), and evaluate its short‐term and long‐term therapeutic effects in mice of different sexes and other AD models (such as 3xTg, 5xFAD, and high‐fat diet‐induced sporadic AD models) to further evaluate its therapeutic potential. Future investigations should focus on examining the combined therapeutic potential of AET with other interventions to enhance cognitive function in AD models, elucidating the relevant molecular pathways, and considering potential clinical applications for treating AD.

## Author Contributions

Conceptualization, Xiangyun Sun; Methodology, Xiangyun Sun; Software, Biao He; Validation, Xiangyun Sun, Shaowen Yu, and Yiyou Yu; Formal Analysis, Xiangyun Sun and Yunliang Wang; Investigation, Yunliang Wang; Resources, Biao He; Data Curation, Yunliang Wang; Writing – Original Draft Preparation, Xiangyun Sun; Writing – Review and Editing, Yunliang Wang; Visualization, Yunliang Wang; Supervision, Yunliang Wang; Project Administration, Xiangyun Sun; Funding Acquisition, Yunliang Wang.

## Ethics Statement

All animal experiments in this study were reviewed and approved by the Animal Ethics Committee of The First Affiliated Hospital of Wannan Medical College (Yijishan Hospital).

## Consent

The authors have nothing to report.

## Conflicts of Interest

The authors declare no conflicts of interest.

## Supporting information


**Data S1:** cns70620‐sup‐0001‐Supinfo1.pdf.

## Data Availability

The data that support the findings of this study are available from the corresponding author upon reasonable request.

## References

[1] A. A. Rostagno , “Pathogenesis of Alzheimer's Disease,” International Journal of Molecular Sciences 24, no. 1 (2022): 107. 36613544 10.3390/ijms24010107 PMC9820480

[2] R. Pluta , S. Januszewski , and S. J. Czuczwar , “Post‐Ischemic Neurodegeneration of the Hippocampus Resembling Alzheimer's Disease Proteinopathy,” International Journal of Molecular Sciences 23, no. 1 (2021): 306. 35008731 10.3390/ijms23010306 PMC8745293

[3] Y. Cai , Y. Chai , Y. Fu , et al., “Salidroside Ameliorates Alzheimer's Disease by Targeting NLRP3 Inflammasome‐Mediated Pyroptosis,” Frontiers in Aging Neuroscience 13 (2021): 809433. 35126093 10.3389/fnagi.2021.809433 PMC8814655

[4] Q. Wang , J. Sun , T. Chen , et al., “Ferroptosis, Pyroptosis, and Cuproptosis in Alzheimer's Disease,” ACS Chemical Neuroscience 14, no. 19 (2023): 3564–3587. 37703318 10.1021/acschemneuro.3c00343

[5] Y. Ju , L. Zhao , S. Li , and Q. Zhao , “The Role of Pyroptosis in Alzheimer's Disease,” Journal of Integrative Neuroscience 22, no. 5 (2023): 129. 37735117 10.31083/j.jin2205129

[6] W. Xue , D. Cui , and Y. Qiu , “Research Progress of Pyroptosis in Alzheimer's Disease,” Frontiers in Molecular Neuroscience 15 (2022): 872471. 35782390 10.3389/fnmol.2022.872471 PMC9244792

[7] H. Bai and Q. Zhang , “Activation of NLRP3 Inflammasome and Onset of Alzheimer's Disease,” Frontiers in Immunology 12 (2021): 701282. 34381452 10.3389/fimmu.2021.701282 PMC8350495

[8] P. Nagar , P. Sharma , R. Dhapola , S. Kumari , B. Medhi , and D. HariKrishnaReddy , “Endoplasmic Reticulum Stress in Alzheimer's Disease: Molecular Mechanisms and Therapeutic Prospects,” Life Sciences 330 (2023): 121983. 37524162 10.1016/j.lfs.2023.121983

[9] C. Hetz and S. Saxena , “ER Stress and the Unfolded Protein Response in Neurodegeneration,” Nature Reviews. Neurology 13, no. 8 (2017): 477–491. 28731040 10.1038/nrneurol.2017.99

[10] J. Yao , H. Yang , H. Wang , et al., “ASPP2 Coordinates ERS‐Mediated Autophagy and Apoptosis Through mTORC1 Pathway in Hepatocyte Injury Induced by TNF‐Alpha,” Frontiers in Pharmacology 13 (2022): 865389. 35418864 10.3389/fphar.2022.865389 PMC8996113

[11] X. Guo , J. Lv , J. Lu , et al., “Protopanaxadiol Derivative DDPU Improves Behavior and Cognitive Deficit in AD Mice Involving Regulation of Both ER Stress and Autophagy,” Neuropharmacology 130 (2018): 77–91. 29197516 10.1016/j.neuropharm.2017.11.033

[12] J. M. Gaitan , H. Y. Moon , M. Stremlau , et al., “Effects of Aerobic Exercise Training on Systemic Biomarkers and Cognition in Late Middle‐Aged Adults at Risk for Alzheimer's Disease,” Frontiers in Endocrinology (Lausanne) 12 (2021): 660181. 10.3389/fendo.2021.660181 PMC8173166 34093436

[13] N. Zhao , X. Zhang , C. Song , Y. Yang , B. He , and B. Xu , “The Effects of Treadmill Exercise on Autophagy in Hippocampus of APP/PS1 Transgenic Mice,” Neuroreport 29, no. 10 (2018): 819–825. 29672446 10.1097/WNR.0000000000001038 PMC5999367

[14] S. Hu , X. Wan , X. Li , and X. Wang , “Aerobic Exercise Alleviates Pyroptosis‐Related Diseases by Regulating NLRP3 Inflammasome,” Frontiers in Physiology 13 (2022): 965366. 36187801 10.3389/fphys.2022.965366 PMC9520335

[15] L. Xu , M. Li , A. Wei , et al., “Treadmill Exercise Promotes E3 Ubiquitin Ligase to Remove Amyloid Beta and P‐Tau and Improve Cognitive Ability in APP/PS1 Transgenic Mice,” Journal of Neuroinflammation 19, no. 1 (2022): 243. 36195875 10.1186/s12974-022-02607-7 PMC9531430

[16] D. Shi , Z. Hao , W. Qi , F. Jiang , K. Liu , and X. Shi , “Aerobic Exercise Combined With Chlorogenic Acid Exerts Neuroprotective Effects and Reverses Cognitive Decline in Alzheimer's Disease Model Mice (APP/PS1) via the SIRT1//PGC‐1alpha/PPARgamma Signaling Pathway,” Frontiers in Aging Neuroscience 15 (2023): 1269952. 38046466 10.3389/fnagi.2023.1269952 PMC10693339

[17] S. Shamsipour , G. Sharifi , and F. Taghian , “An 8‐Week Administration of Bifidobacterium Bifidum and *Lactobacillus Plantarum* Combined With Exercise Training Alleviates Neurotoxicity of Abeta and Spatial Learning via Acetylcholine in Alzheimer Rat Model,” Journal of Molecular Neuroscience 71, no. 7 (2021): 1495–1505. 33715084 10.1007/s12031-021-01812-y

[18] Y. Wu , Q. Chen , B. Wen , N. Wu , B. He , and J. Chen , “Berberine Reduces Abeta(42) Deposition and Tau Hyperphosphorylation via Ameliorating Endoplasmic Reticulum Stress,” Frontiers in Pharmacology 12 (2021): 640758. 34349640 10.3389/fphar.2021.640758 PMC8327086

[19] C. C. Deng , Y. Liang , M. S. Wu , et al., “Nigericin Selectively Targets Cancer Stem Cells in Nasopharyngeal Carcinoma,” International Journal of Biochemistry & Cell Biology 45, no. 9 (2013): 1997–2006. 23831840 10.1016/j.biocel.2013.06.023

[20] S. T. Wu , S. S. Han , X. M. Xu , et al., “3‐Methyladenine Ameliorates Surgery‐Induced Anxiety‐Like Behaviors in Aged Mice by Inhibiting Autophagy‐Induced Excessive Oxidative Stress,” Metabolic Brain Disease 38, no. 6 (2023): 1913–1923. 37097438 10.1007/s11011-023-01217-3

[21] Z. S. Wang , F. E. Lu , L. J. Xu , and H. Dong , “Berberine Reduces Endoplasmic Reticulum Stress and Improves Insulin Signal Transduction in Hep G2 Cells,” Acta Pharmacologica Sinica 31, no. 5 (2010): 578–584. 20383171 10.1038/aps.2010.30 PMC4002745

[22] J. Bruch , H. Xu , T. W. Rosler , et al., “PERK Activation Mitigates Tau Pathology In Vitro and In Vivo,” EMBO Molecular Medicine 9, no. 3 (2017): 371–384. 28148553 10.15252/emmm.201606664 PMC5331260

[23] X. Zhou , W. Xiao , Z. Su , et al., “Hippocampal Proteomic Alteration in Triple Transgenic Mouse Model of Alzheimer's Disease and Implication of PINK 1 Regulation in Donepezil Treatment,” Journal of Proteome Research 18, no. 4 (2019): 1542–1552. 30484658 10.1021/acs.jproteome.8b00818

[24] C. V. Vorhees and M. T. Williams , “Morris Water Maze: Procedures for Assessing Spatial and Related Forms of Learning and Memory,” Nature Protocols 1, no. 2 (2006): 848–858. 17406317 10.1038/nprot.2006.116 PMC2895266

[25] C. Z. Yang , S. H. Wang , R. H. Zhang , et al., “Neuroprotective Effect of Astragalin via Activating PI3K/Akt‐mTOR‐Mediated Autophagy on APP/PS1 Mice,” Cell Death Discovery 9, no. 1 (2023): 15. 36681681 10.1038/s41420-023-01324-1 PMC9867706

[26] Z. Shi , L. Zhu , T. Li , et al., “Neuroprotective Mechanisms of *Lycium barbarum* Polysaccharides Against Ischemic Insults by Regulating NR2B and NR2A Containing NMDA Receptor Signaling Pathways,” Frontiers in Cellular Neuroscience 11 (2017): 288. 29021742 10.3389/fncel.2017.00288 PMC5623723

[27] C. Dempsey , A. Rubio Araiz , K. J. Bryson , et al., “Inhibiting the NLRP3 Inflammasome With MCC950 Promotes Non‐Phlogistic Clearance of Amyloid‐Beta and Cognitive Function in APP/PS1 Mice,” Brain, Behavior, and Immunity 61 (2017): 306–316. 28003153 10.1016/j.bbi.2016.12.014

[28] M. S. Rao and A. K. Shetty , “Efficacy of Doublecortin as a Marker to Analyse the Absolute Number and Dendritic Growth of Newly Generated Neurons in the Adult Dentate Gyrus,” European Journal of Neuroscience 19, no. 2 (2004): 234–246. 14725617 10.1111/j.0953-816x.2003.03123.x

[29] R. Zhou , A. S. Yazdi , P. Menu , and J. Tschopp , “A Role for Mitochondria in NLRP3 Inflammasome Activation,” Nature 469, no. 7329 (2011): 221–225. 21124315 10.1038/nature09663

[30] Y. Kouroku , E. Fujita , I. Tanida , et al., “ER Stress (PERK/eIF2alpha Phosphorylation) Mediates the Polyglutamine‐Induced LC3 Conversion, an Essential Step for Autophagy Formation,” Cell Death and Differentiation 14, no. 2 (2007): 230–239. 16794605 10.1038/sj.cdd.4401984

[31] S. Lopez‐Ortiz , P. L. Valenzuela , M. M. Seisdedos , et al., “Exercise Interventions in Alzheimer's Disease: A Systematic Review and Meta‐Analysis of Randomized Controlled Trials,” Ageing Research Reviews 72 (2021): 101479. 34601135 10.1016/j.arr.2021.101479

[32] S. Lopez‐Ortiz , S. Lista , P. L. Valenzuela , et al., “Effects of Physical Activity and Exercise Interventions on Alzheimer's Disease: An Umbrella Review of Existing Meta‐Analyses,” Journal of Neurology 270, no. 2 (2023): 711–725. 36342524 10.1007/s00415-022-11454-8

[33] M. V. F. Silva , C. M. G. Loures , L. C. V. Alves , L. C. de Souza , K. B. G. Borges , and M. D. G. Carvalho , “Alzheimer's Disease: Risk Factors and Potentially Protective Measures,” Journal of Biomedical Science 26, no. 1 (2019): 33. 31072403 10.1186/s12929-019-0524-y PMC6507104

[34] G. Garcia‐Escobar , R. M. Manero , A. Fernandez‐Lebrero , et al., “Blood Biomarkers of Alzheimer's Disease and Cognition: A Literature Review,” Biomolecules 14, no. 1 (2024): 93. 38254693 10.3390/biom14010093 PMC10813472

[35] G. A. Panza , B. A. Taylor , H. V. MacDonald , et al., “Can Exercise Improve Cognitive Symptoms of Alzheimer's Disease?,” Journal of the American Geriatrics Society 66, no. 3 (2018): 487–495. 29363108 10.1111/jgs.15241

[36] J. K. Morris , E. D. Vidoni , D. K. Johnson , et al., “Aerobic Exercise for Alzheimer's Disease: A Randomized Controlled Pilot Trial,” PLoS One 12, no. 2 (2017): e0170547. 28187125 10.1371/journal.pone.0170547 PMC5302785

[37] S. Ayari , A. Abellard , M. Carayol , E. Guedj , and O. Gavarry , “A Systematic Review of Exercise Modalities That Reduce Pro‐Inflammatory Cytokines in Humans and Animals' Models With Mild Cognitive Impairment or Dementia,” Experimental Gerontology 175 (2023): 112141. 36898593 10.1016/j.exger.2023.112141

[38] M. Dicarlo , P. Pignataro , C. Zecca , et al., “Irisin Levels in Cerebrospinal Fluid Correlate With Biomarkers and Clinical Dementia Scores in Alzheimer Disease,” Annals of Neurology 96, no. 1 (2024): 61–73. 38780366 10.1002/ana.26946

[39] S. Zhang , K. Zhen , Q. Su , Y. Chen , Y. Lv , and L. Yu , “The Effect of Aerobic Exercise on Cognitive Function in People With Alzheimer's Disease: A Systematic Review and Meta‐Analysis of Randomized Controlled Trials,” International Journal of Environmental Research and Public Health 19, no. 23 (2022): 15700. 36497772 10.3390/ijerph192315700 PMC9736612

[40] R. X. Jia , J. H. Liang , Y. Xu , and Y. Q. Wang , “Effects of Physical Activity and Exercise on the Cognitive Function of Patients With Alzheimer Disease: A Meta‐Analysis,” BMC Geriatrics 19, no. 1 (2019): 181. 31266451 10.1186/s12877-019-1175-2 PMC6604129

[41] J. Yang , L. Wise , and K. I. Fukuchi , “TLR4 Cross‐Talk With NLRP3 Inflammasome and Complement Signaling Pathways in Alzheimer's Disease,” Frontiers in Immunology 11 (2020): 724. 32391019 10.3389/fimmu.2020.00724 PMC7190872

[42] S. Tao , W. Fan , J. Liu , et al., “NLRP3 Inflammasome: An Emerging Therapeutic Target for Alzheimer's Disease,” Journal of Alzheimer's Disease 96, no. 4 (2023): 1383–1398. 10.3233/JAD-230567 37980662

[43] R. M. McManus and E. Latz , “NLRP3 Inflammasome Signalling in Alzheimer's Disease,” Neuropharmacology 252 (2024): 109941. 38565393 10.1016/j.neuropharm.2024.109941

[44] M. T. Milner , M. Maddugoda , J. Gotz , S. S. Burgener , and K. Schroder , “The NLRP3 Inflammasome Triggers Sterile Neuroinflammation and Alzheimer's Disease,” Current Opinion in Immunology 68 (2021): 116–124. 33181351 10.1016/j.coi.2020.10.011

[45] N. Kelley , D. Jeltema , Y. Duan , and Y. He , “The NLRP3 Inflammasome: An Overview of Mechanisms of Activation and Regulation,” International Journal of Molecular Sciences 20, no. 13 (2019): 3328. 31284572 10.3390/ijms20133328 PMC6651423

[46] M. Golzari‐Sorkheh , C. E. Brown , D. F. Weaver , and M. A. Reed , “The NLRP3 Inflammasome in the Pathogenesis and Treatment of Alzheimer's Disease,” Journal of Alzheimer's Disease 84, no. 2 (2021): 579–598. 10.3233/JAD-210660 34569958

[47] Z. Zhang , X. Yang , Y. Q. Song , and J. Tu , “Autophagy in Alzheimer's Disease Pathogenesis: Therapeutic Potential and Future Perspectives,” Ageing Research Reviews 72 (2021): 101464. 34551326 10.1016/j.arr.2021.101464

[48] Z. Cao , Y. Wang , Z. Long , and G. He , “Interaction Between Autophagy and the NLRP3 Inflammasome,” Acta Biochimica et Biophysica Sinica 51, no. 11 (2019): 1087–1095. 31609412 10.1093/abbs/gmz098

[49] X. W. Zhang , X. X. Zhu , D. S. Tang , and J. H. Lu , “Targeting Autophagy in Alzheimer's Disease: Animal Models and Mechanisms,” Zoological Research 44, no. 6 (2023): 1132–1145. 37963840 10.24272/j.issn.2095-8137.2023.294 PMC10802106

[50] T. Panagaki , S. Gengler , and C. Holscher , “The Novel DA‐CH3 Dual Incretin Restores Endoplasmic Reticulum Stress and Autophagy Impairments to Attenuate Alzheimer‐Like Pathology and Cognitive Decrements in the APPSWE/PS1DeltaE9 Mouse Model,” Journal of Alzheimer's Disease 66, no. 1 (2018): 195–218. 10.3233/JAD-180584 30282365

[51] M. Wang , H. Zhang , J. Liang , J. Huang , and N. Chen , “Exercise Suppresses Neuroinflammation for Alleviating Alzheimer's Disease,” Journal of Neuroinflammation 20, no. 1 (2023): 76. 36935511 10.1186/s12974-023-02753-6 PMC10026496

[52] M. S. de Sousa Fernandes , G. Badicu , G. C. J. Santos , et al., “Physical Exercise Decreases Endoplasmic Reticulum Stress in Central and Peripheral Tissues of Rodents: A Systematic Review,” European Journal of Investigation in Health, Psychology and Education 13, no. 6 (2023): 1082–1096. 37366786 10.3390/ejihpe13060082 PMC10297180

[53] M. Ohno , “PERK as a Hub of Multiple Pathogenic Pathways Leading to Memory Deficits and Neurodegeneration in Alzheimer's Disease,” Brain Research Bulletin 141 (2018): 72–78. 28804008 10.1016/j.brainresbull.2017.08.007

[54] W. Chen , M. Ma , Y. Song , et al., “Exercise Attenuates Myocardial Ischemia‐Reperfusion Injury by Regulating Endoplasmic Reticulum Stress and Mitophagy Through M(2) Acetylcholine Receptor,” Antioxidants & Redox Signaling 40, no. 4–6 (2024): 209–221. 37294203 10.1089/ars.2022.0168

[55] A. R. Baek , J. Hong , K. S. Song , et al., “Spermidine Attenuates Bleomycin‐Induced Lung Fibrosis by Inducing Autophagy and Inhibiting Endoplasmic Reticulum Stress (ERS)‐Induced Cell Death in Mice,” Experimental & Molecular Medicine 52, no. 12 (2020): 2034–2045. 33318630 10.1038/s12276-020-00545-z PMC8080799

[56] W. Zhang , D. Xiao , Q. Mao , and H. Xia , “Role of Neuroinflammation in Neurodegeneration Development,” Signal Transduction and Targeted Therapy 8, no. 1 (2023): 267. 37433768 10.1038/s41392-023-01486-5 PMC10336149

[57] E. D. Vidoni , J. Perales , M. Alshehri , A. M. Giles , C. F. Siengsukon , and J. M. Burns , “Aerobic Exercise Sustains Performance of Instrumental Activities of Daily Living in Early‐Stage Alzheimer Disease,” Journal of Geriatric Physical Therapy 42, no. 3 (2019): E129–E134. 29286983 10.1519/JPT.0000000000000172 PMC6023779

[58] I. Terao and W. Kodama , “Comparative Efficacy, Tolerability, and Acceptability of Donanemab, Lecanemab, Aducanumab, Melatonin, and Aerobic Exercise for a Short Time on Cognitive Function in Mild Cognitive Impairment and Mild Alzheimer's Disease: A Systematic Review and Network Meta‐Analysis,” Journal of Alzheimer's Disease 98, no. 3 (2024): 825–835. 10.3233/JAD-230911 38461503

[59] S. H. Yu , S. D. Park , and K. C. Sim , “The Effect of tDCS on Cognition and Neurologic Recovery of Rats With Alzheimer's Disease,” Journal of Physical Therapy Science 26, no. 2 (2014): 247–249. 24648641 10.1589/jpts.26.247 PMC3944298

[60] C. S. Liu , N. Herrmann , B. X. Song , et al., “Exercise Priming With Transcranial Direct Current Stimulation: A Study Protocol for a Randomized, Parallel‐Design, Sham‐Controlled Trial in Mild Cognitive Impairment and Alzheimer's Disease,” BMC Geriatrics 21, no. 1 (2021): 677. 34863115 10.1186/s12877-021-02636-6 PMC8645072

