# Supplementary material for: Aerobic Exercise Training Exerts Neuroprotective Effects in Alzheimer's Disease Mice by Regulating Endoplasmic Reticulum Stress‐Autophagy Pathway‐Mediated Pyroptosis
Source: CNS Neurosci Ther. 2025 Nov 17;31(11):e70620. doi: 10.1111/cns.70620 (PMC12623150; doi:10.1111/cns.70620)

Full unedited gel/blot for Figure 1D

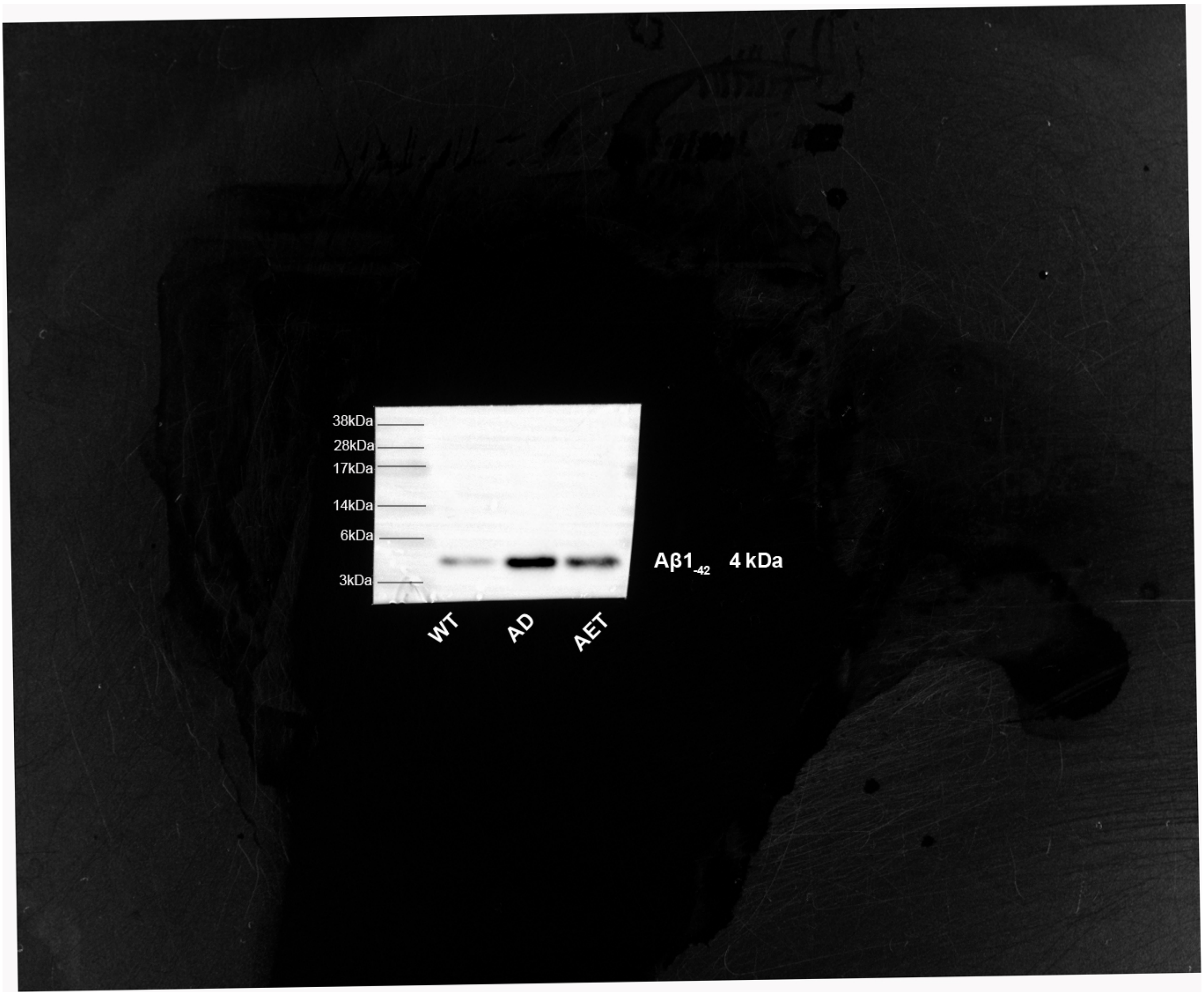

Full unedited gel/blot for Figure 1D

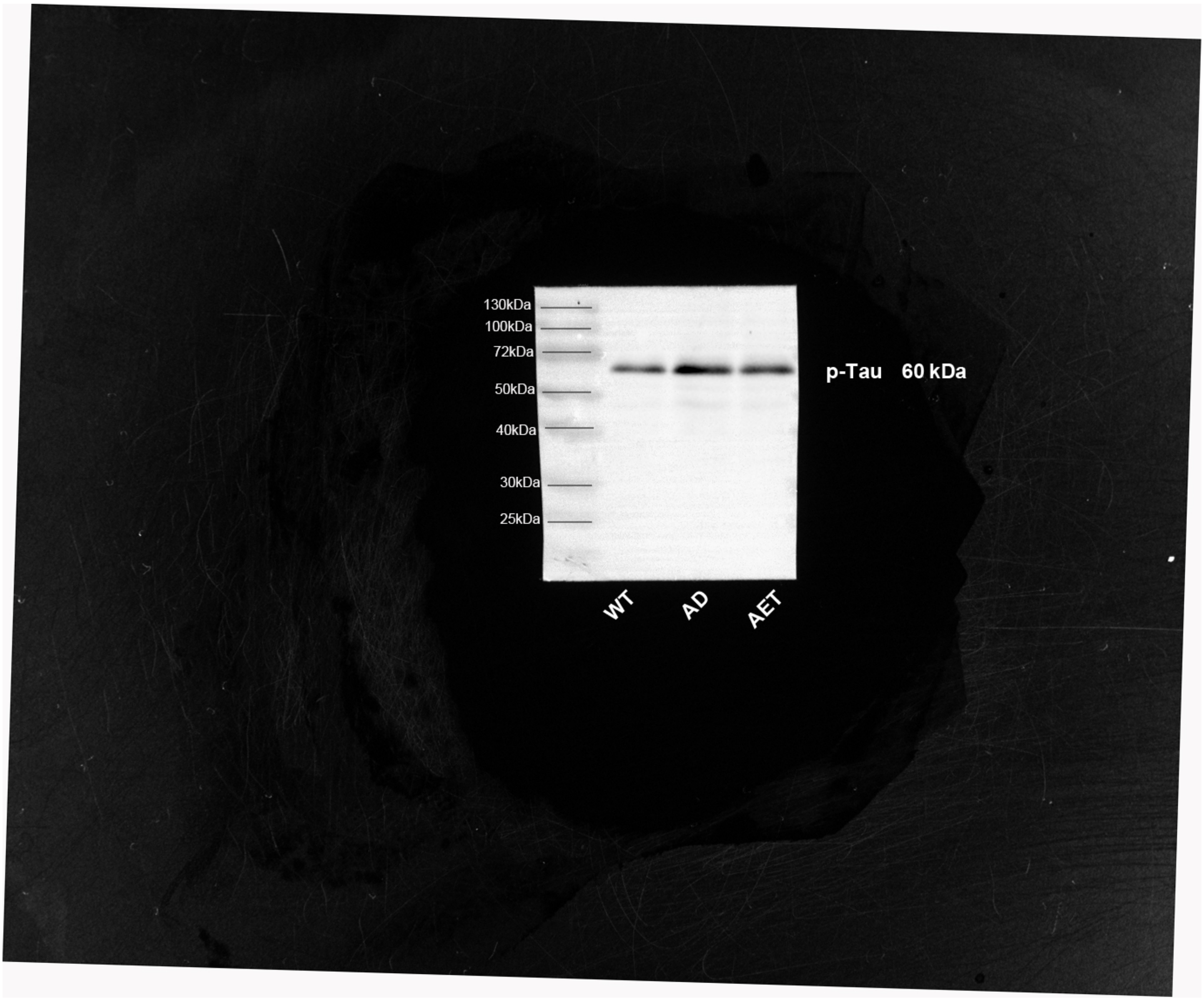

Full unedited gel/blot for Figure 1D

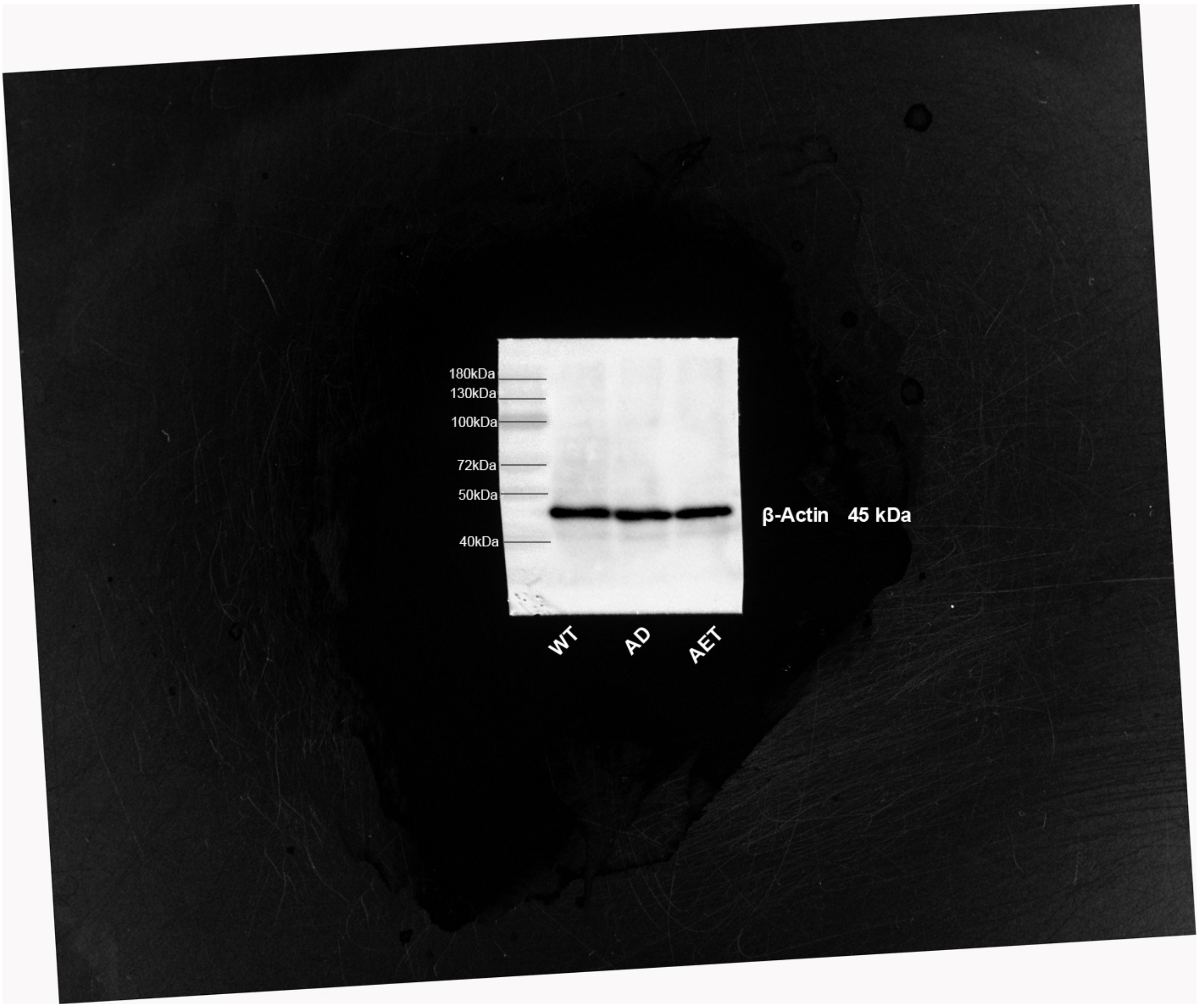

Full unedited gel/blot for Figure 2E

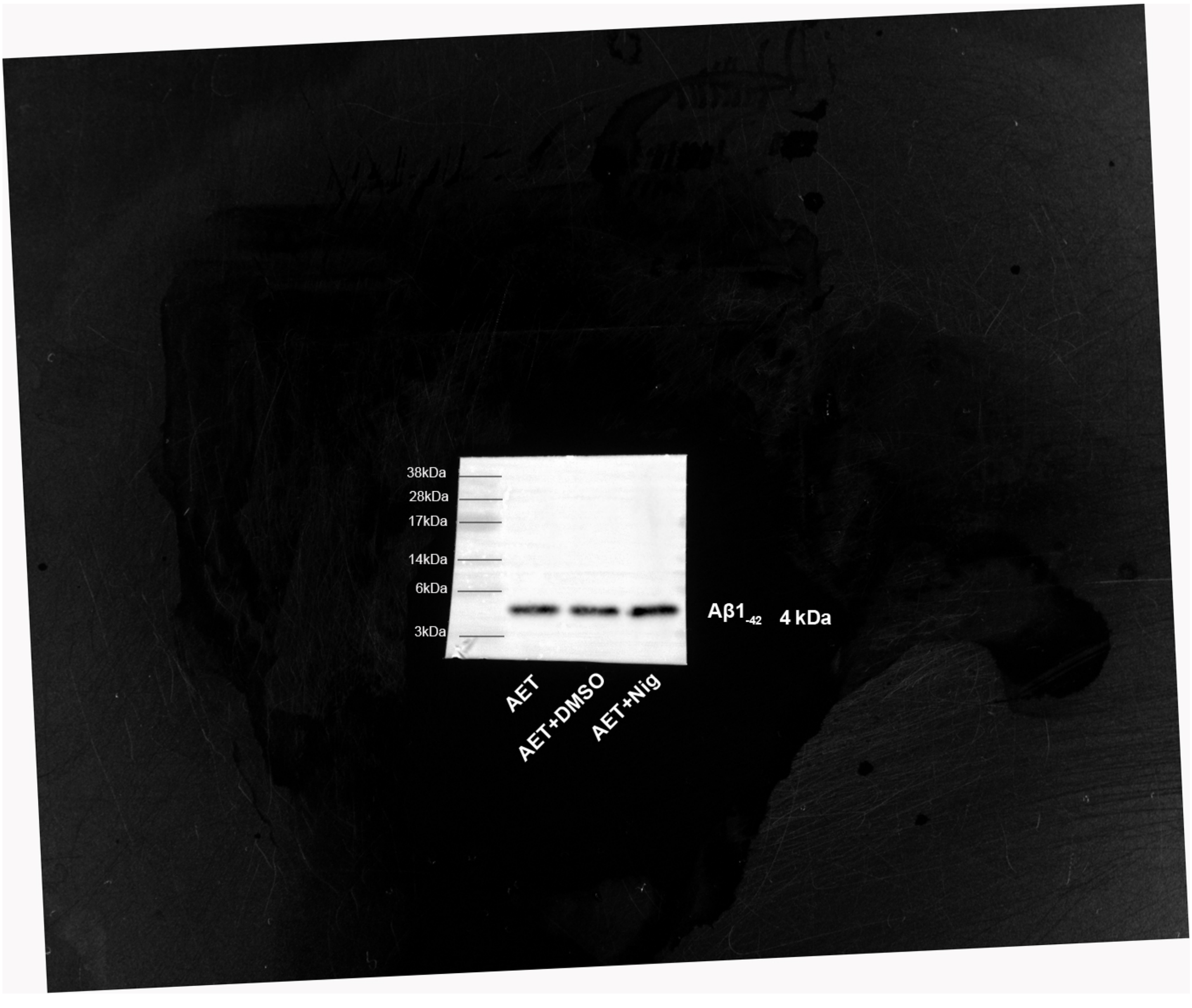

Full unedited gel/blot for Figure 2E

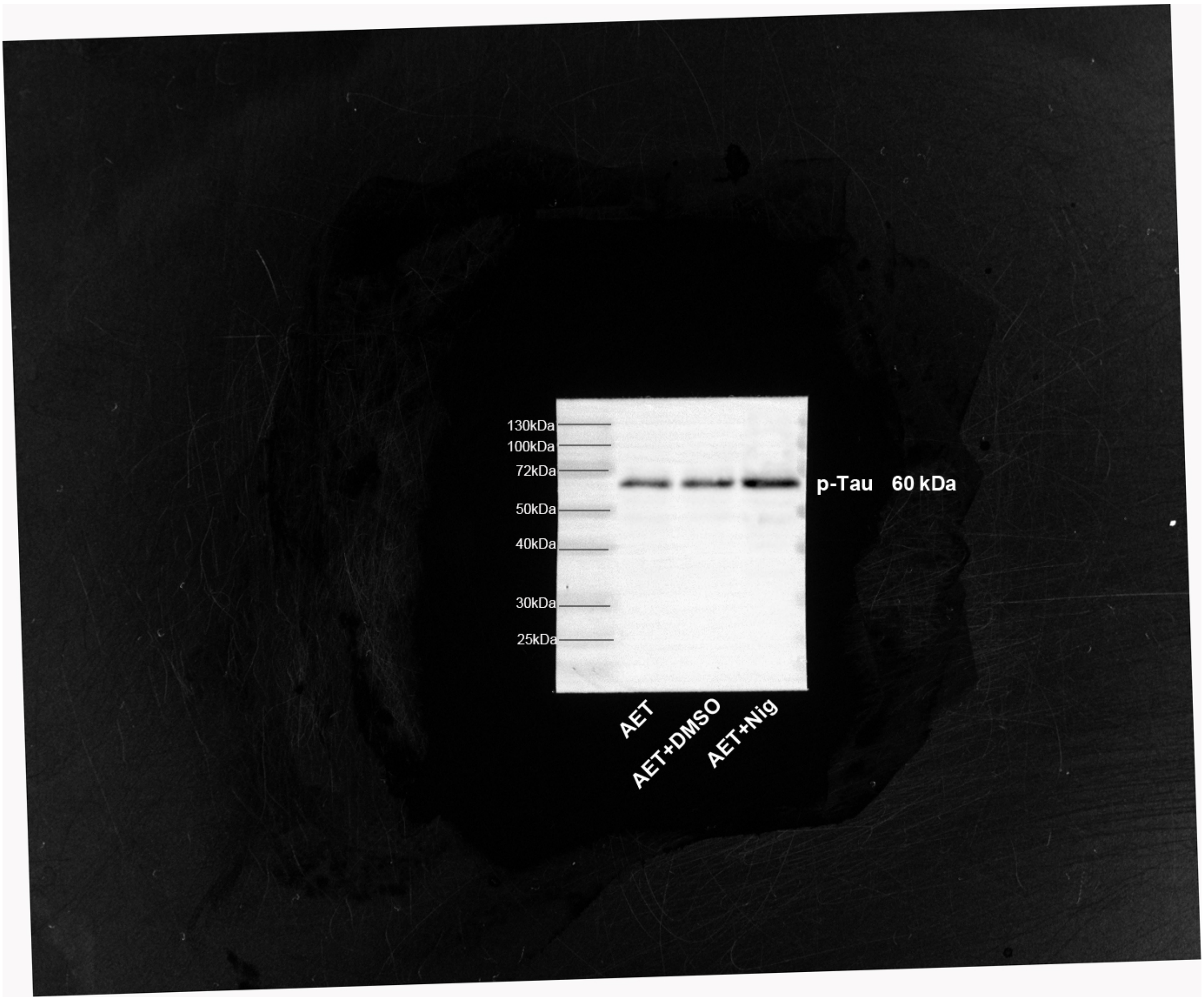

Full unedited gel/blot for Figure 2E

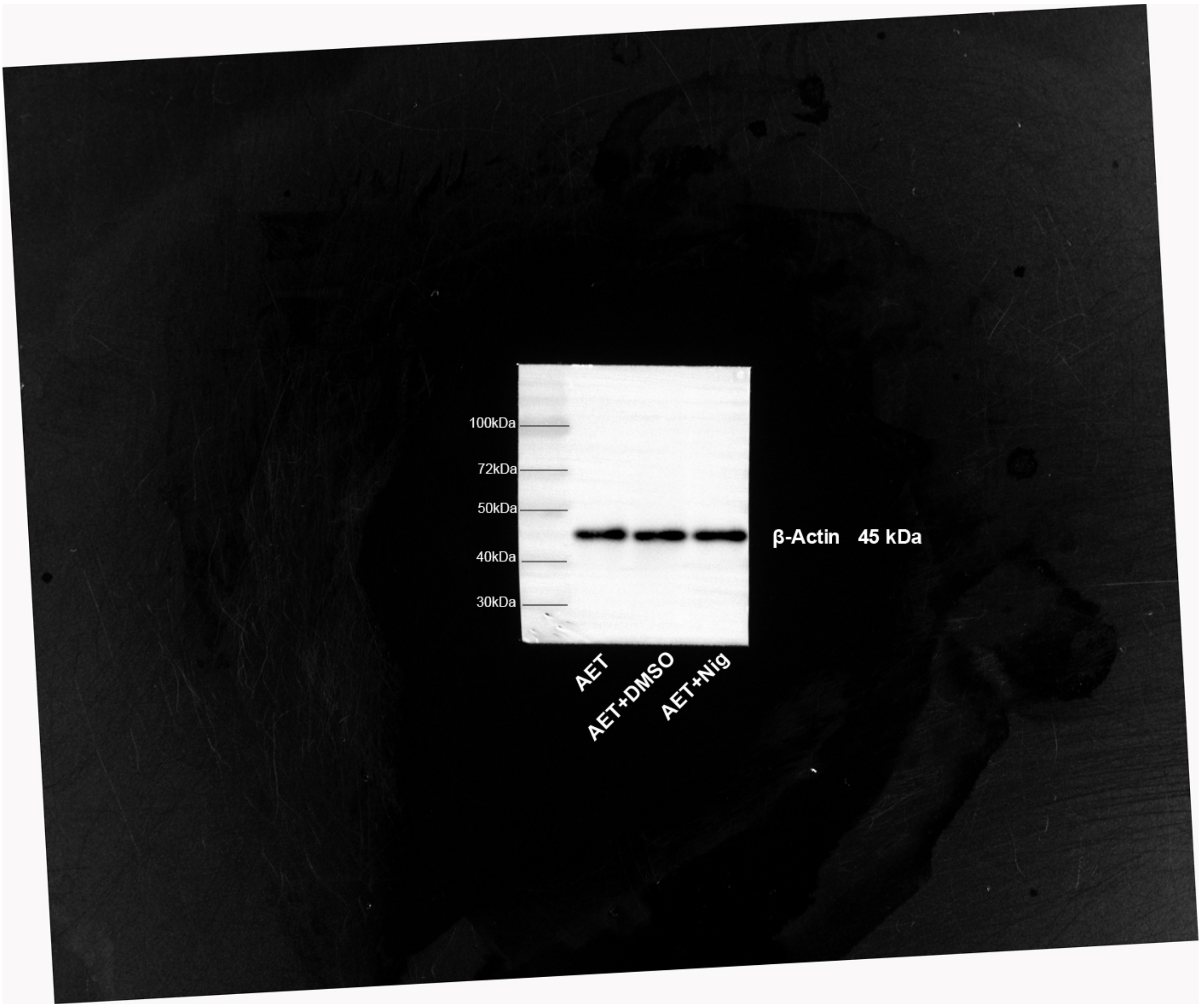

Full unedited gel/blot for Figure 3

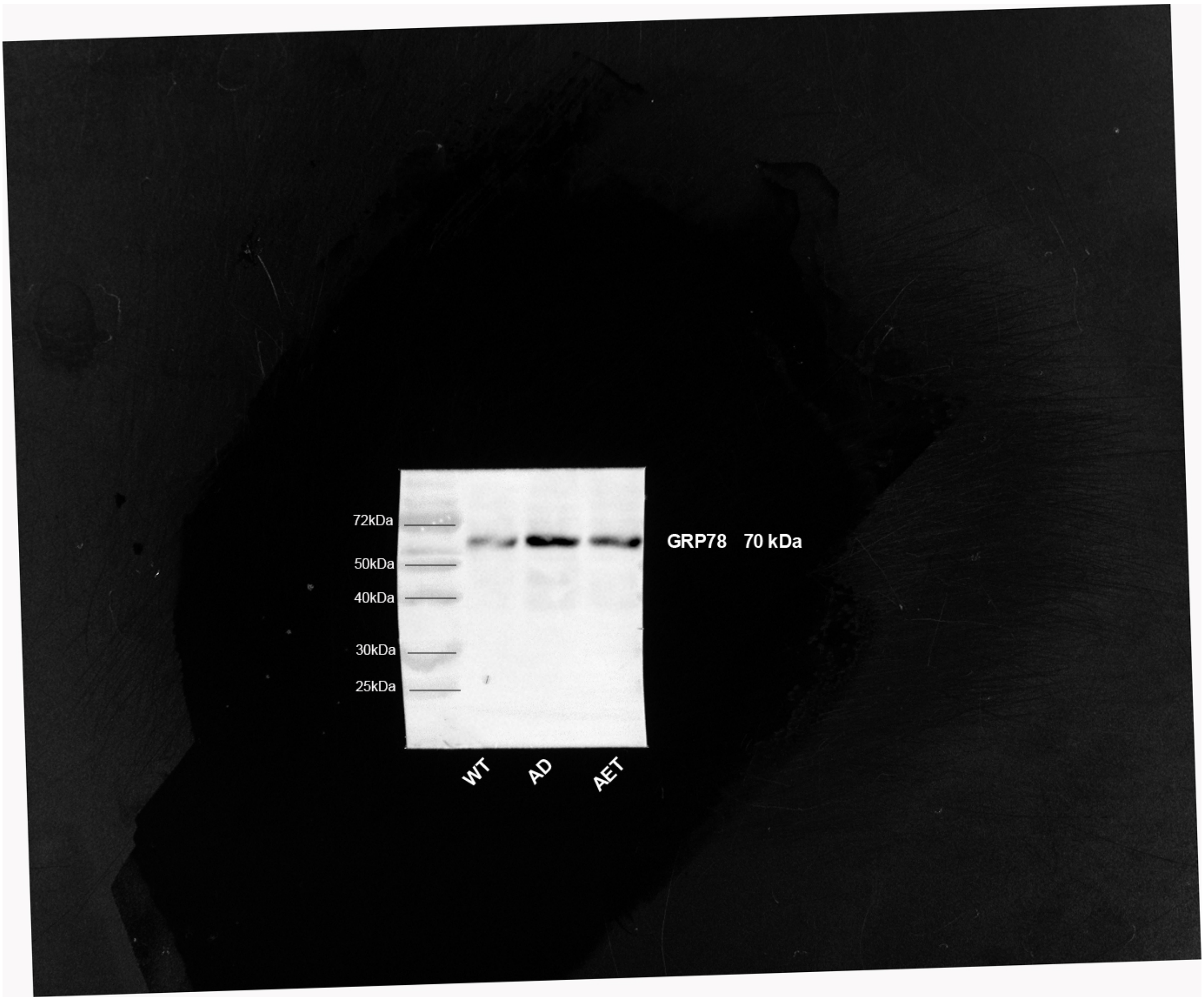

Full unedited gel/blot for Figure 3

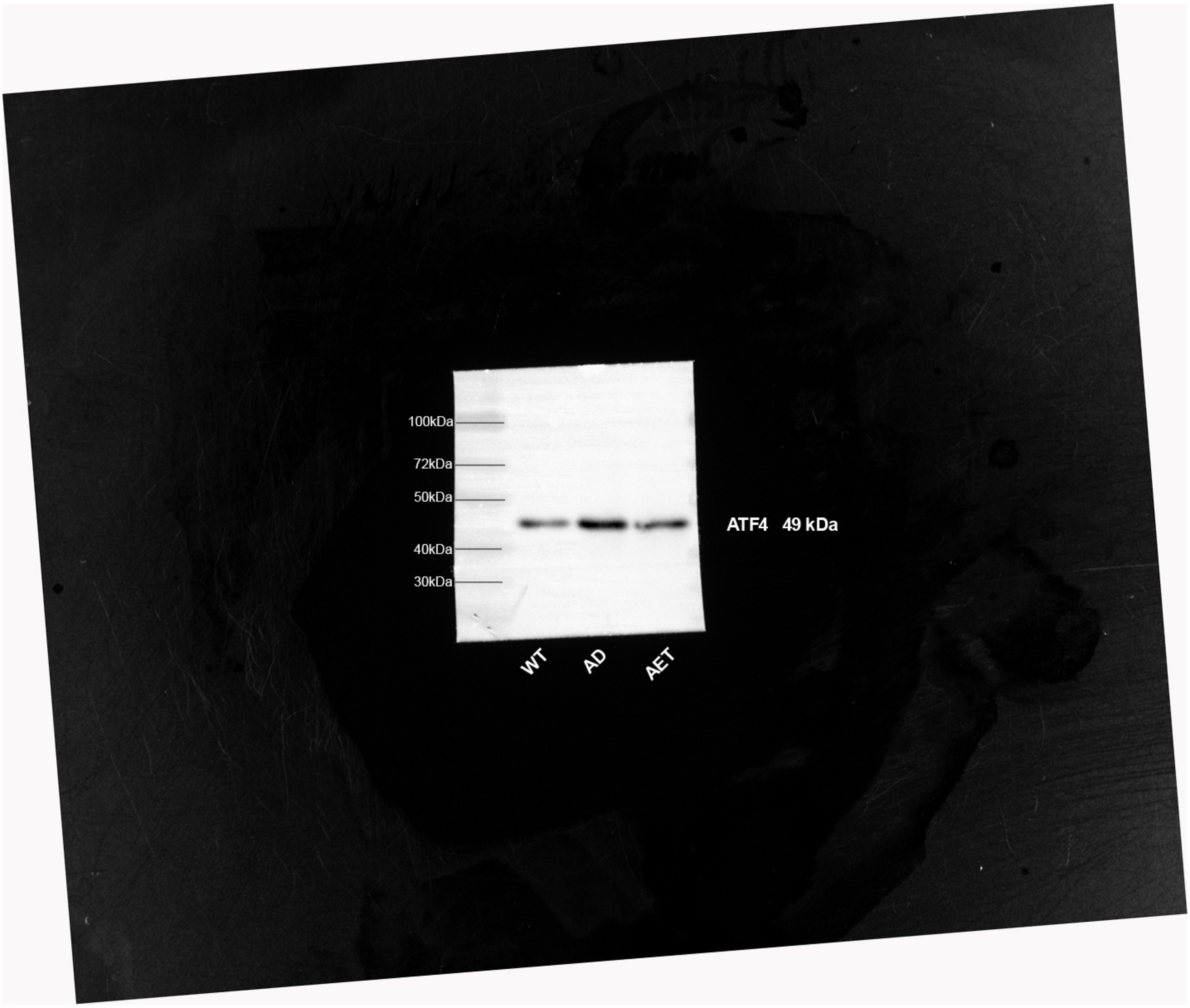

Full unedited gel/blot for Figure 3

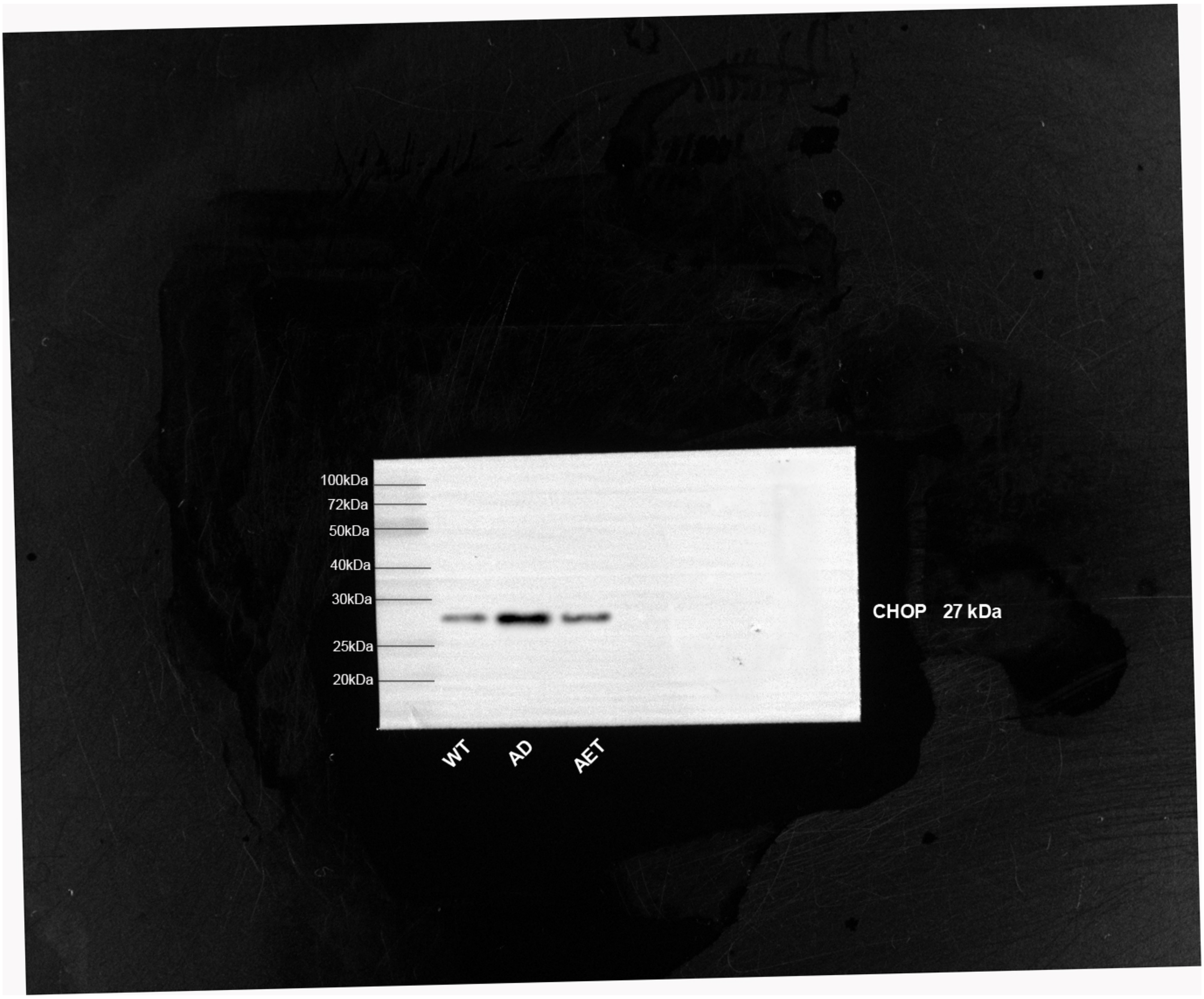

Full unedited gel/blot for Figure 3

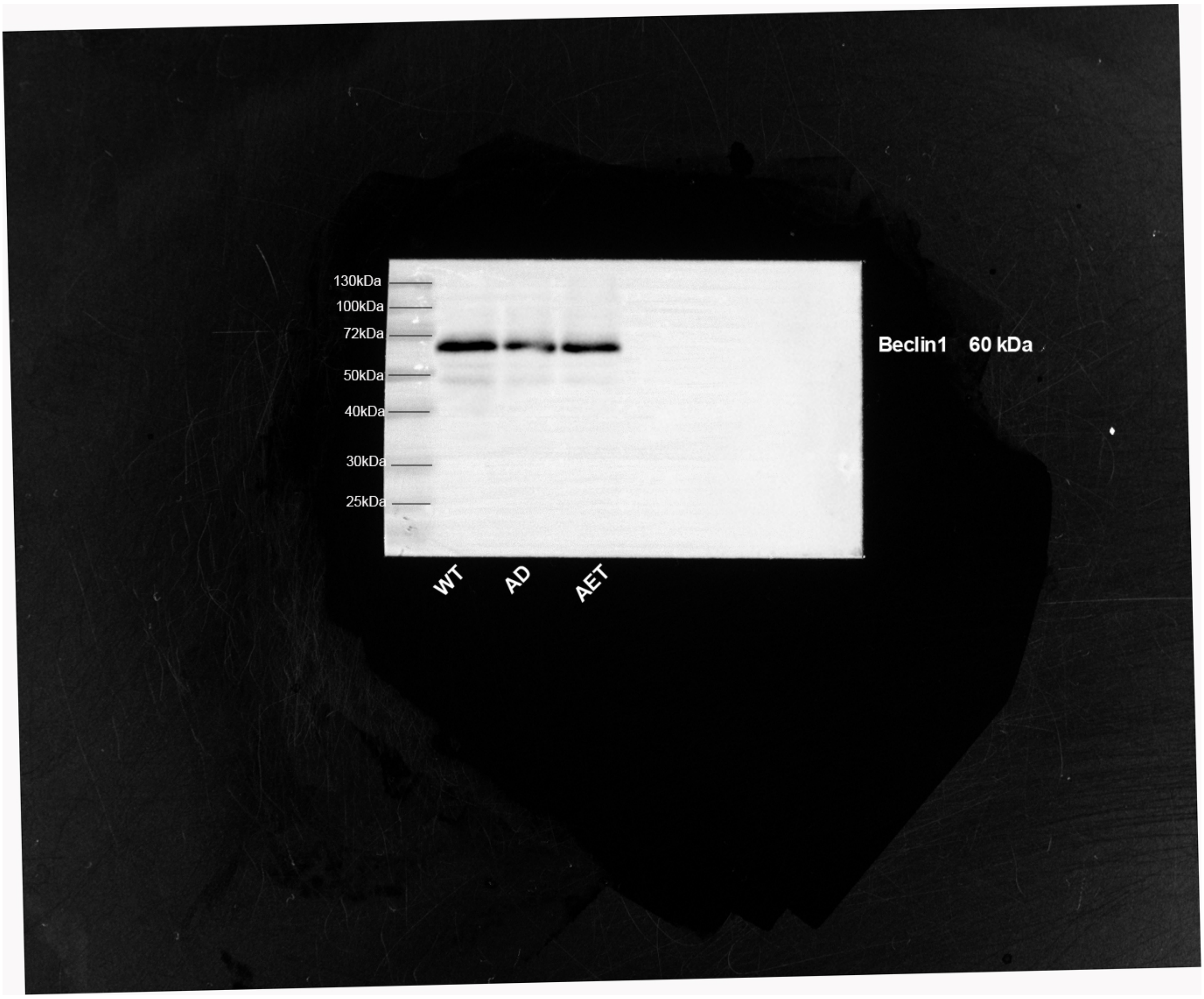

Full unedited gel/blot for Figure 3

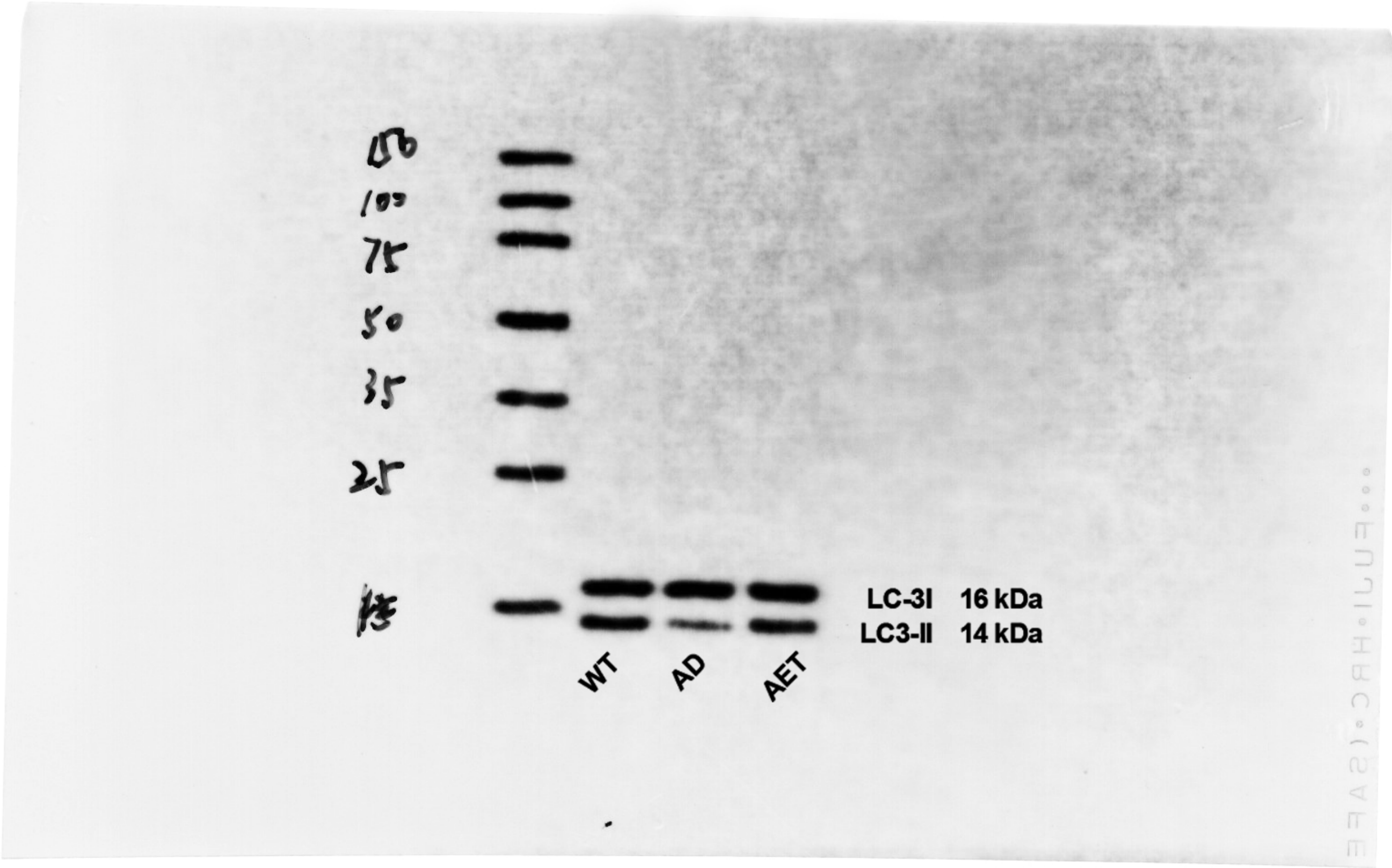

Full unedited gel/blot for Figure 3

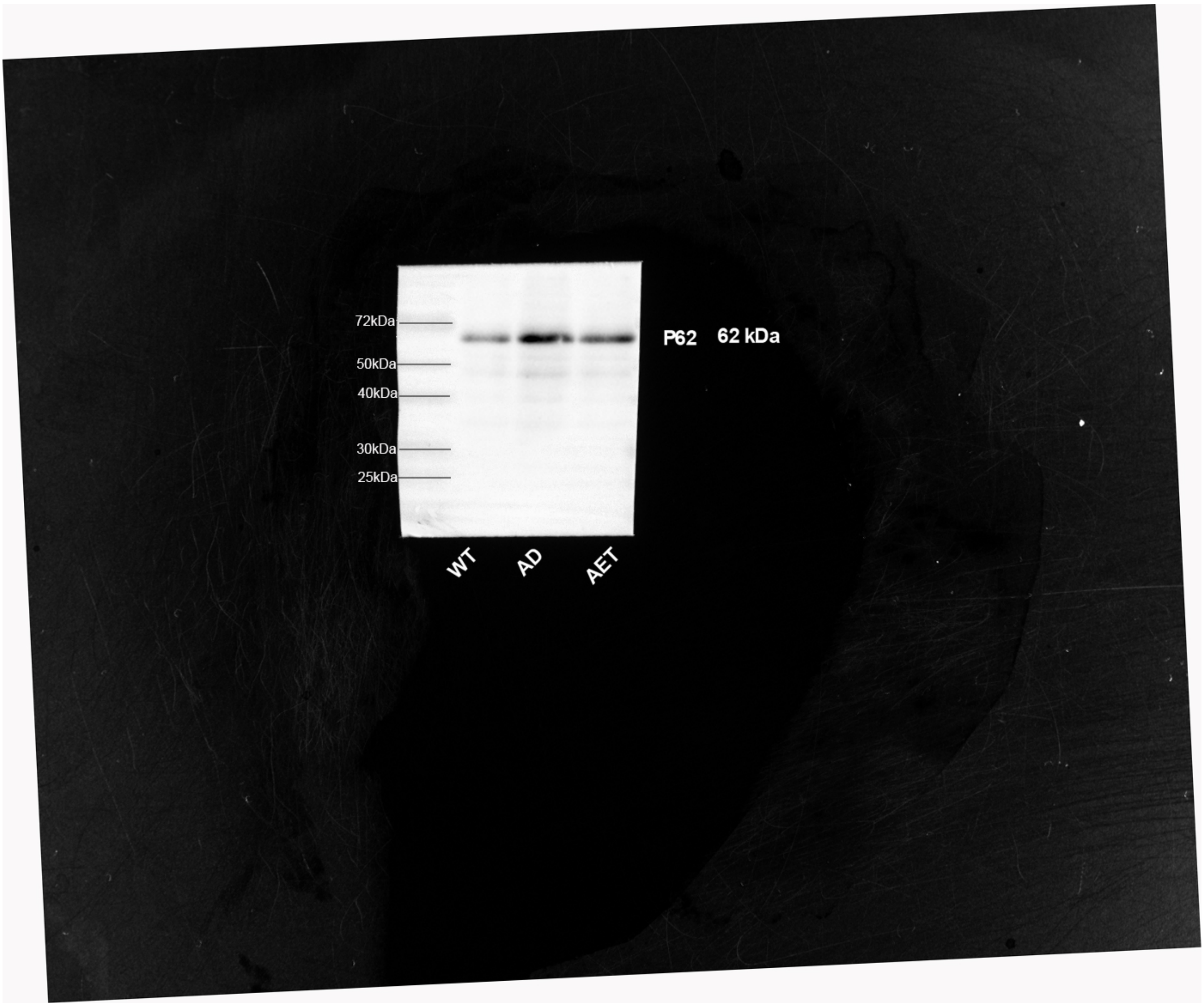

Full unedited gel/blot for Figure 3

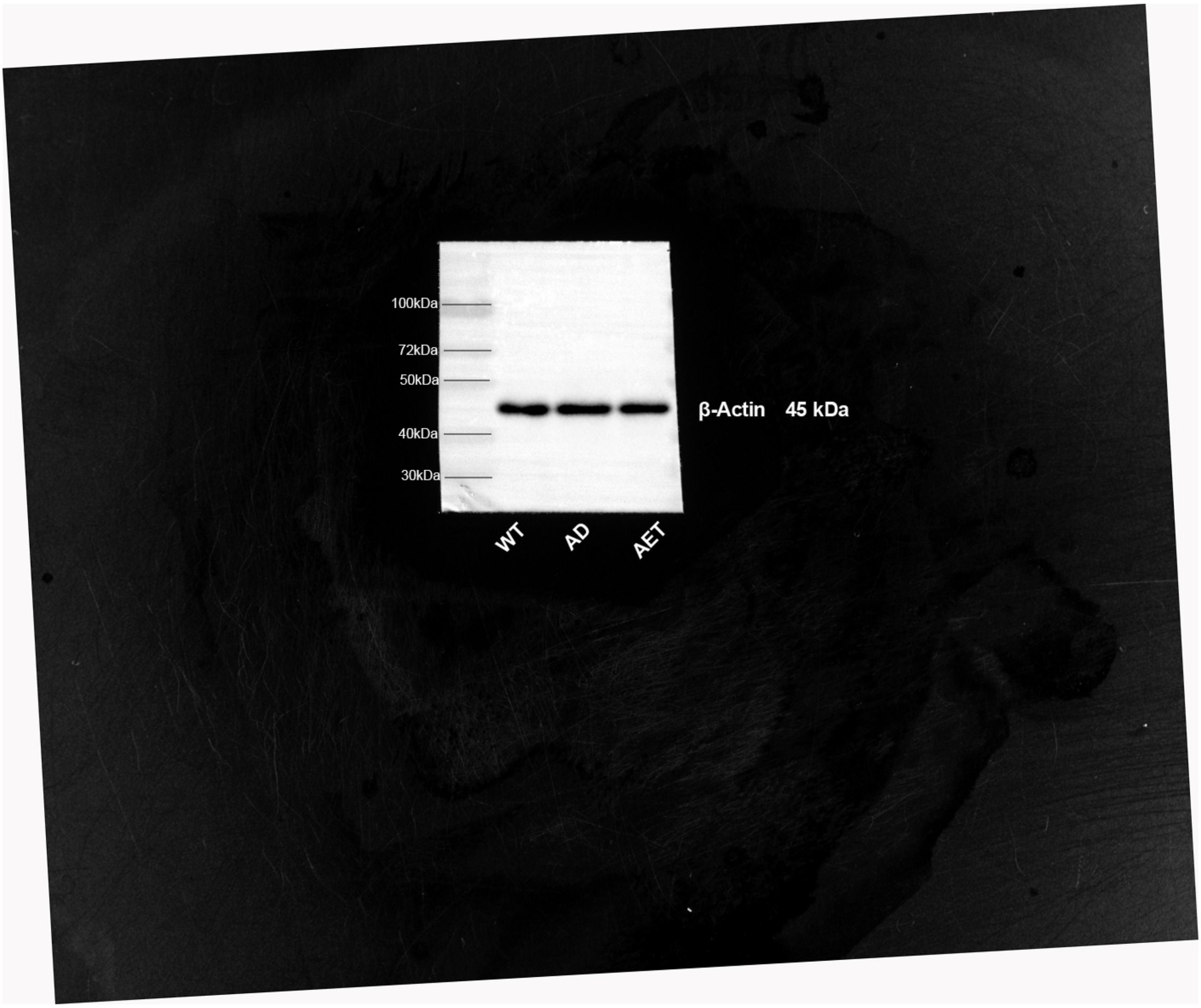

Full unedited gel/blot for Figure 4A

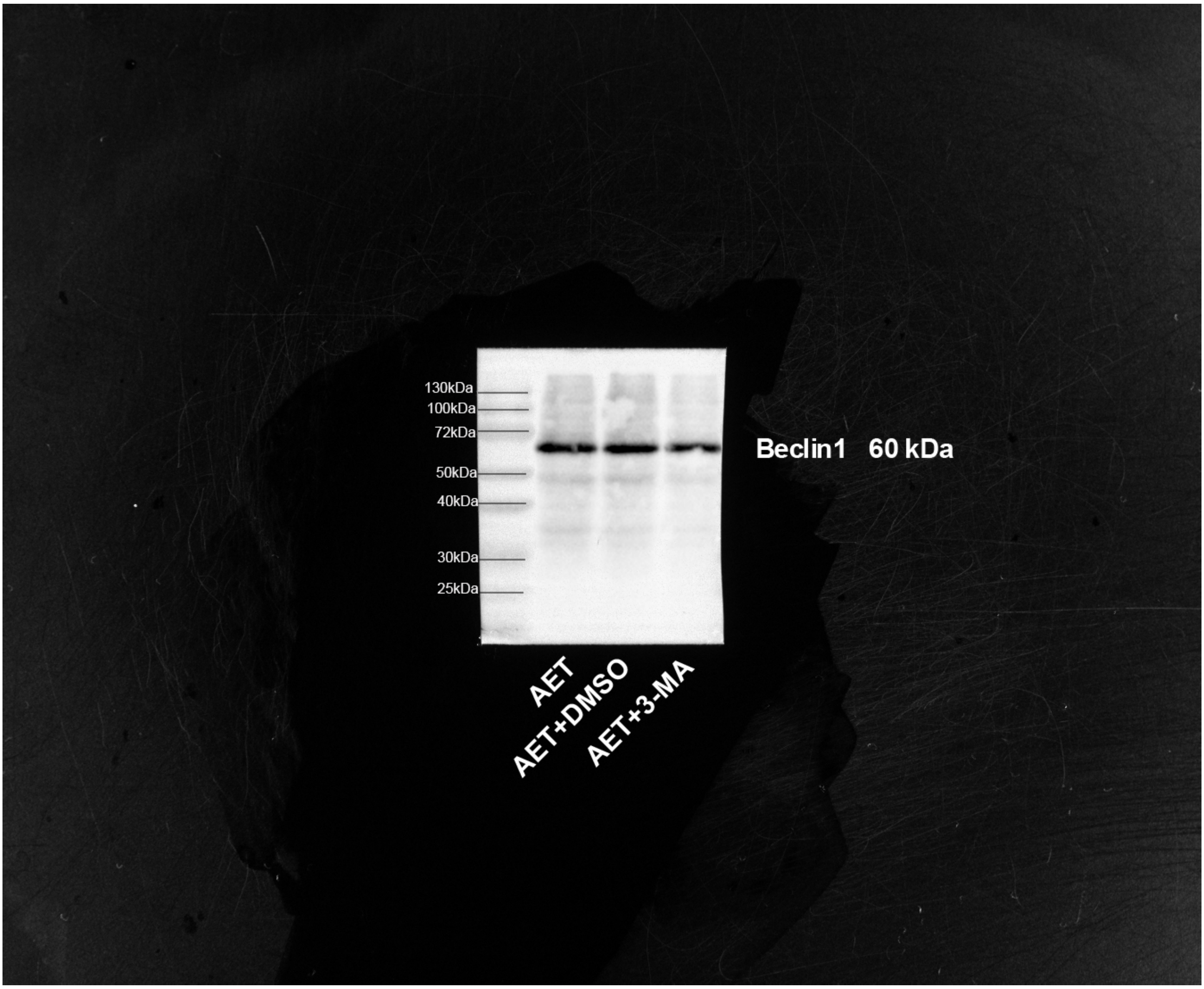

Full unedited gel/blot for Figure 4A

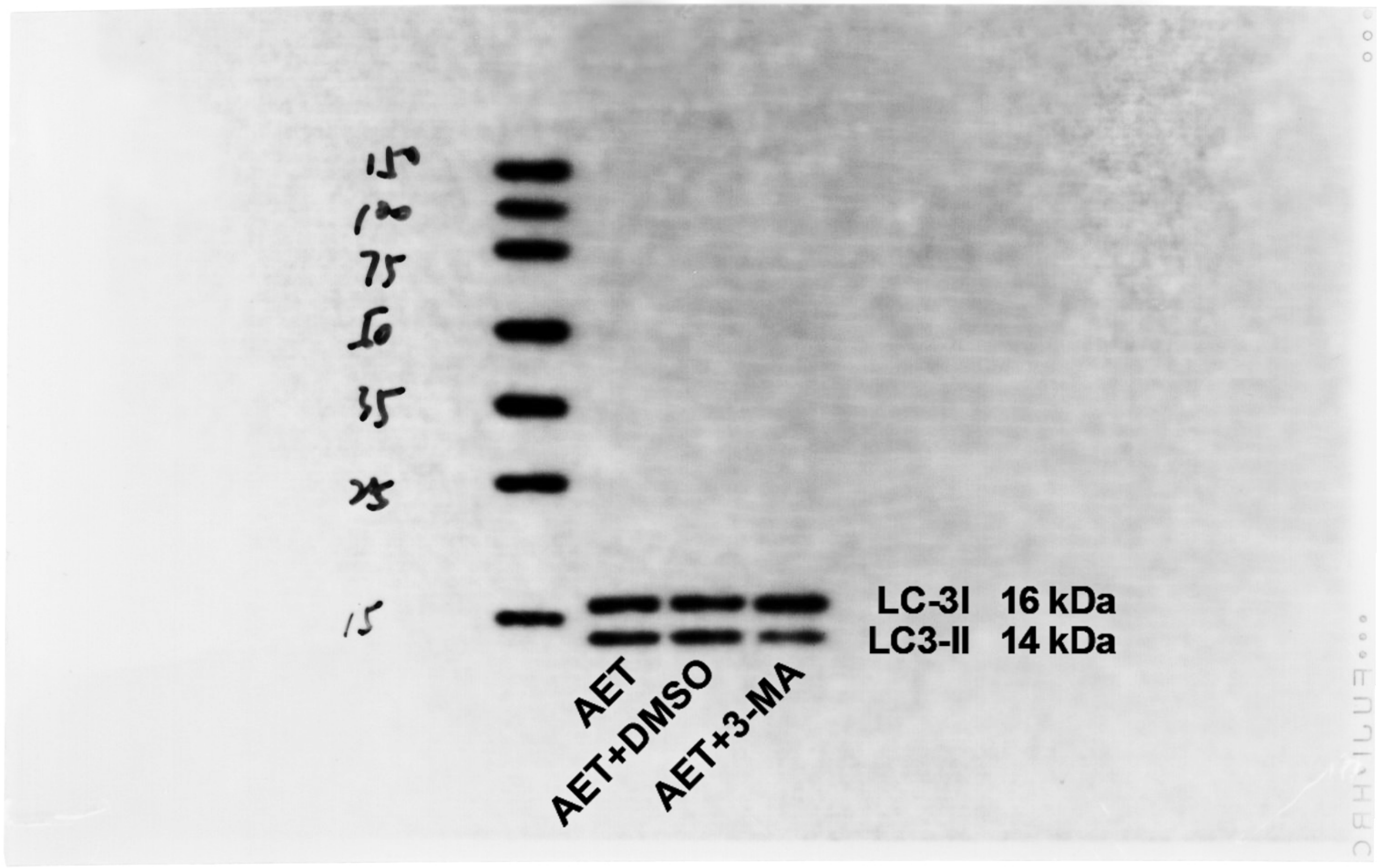

Full unedited gel/blot for Figure 4A

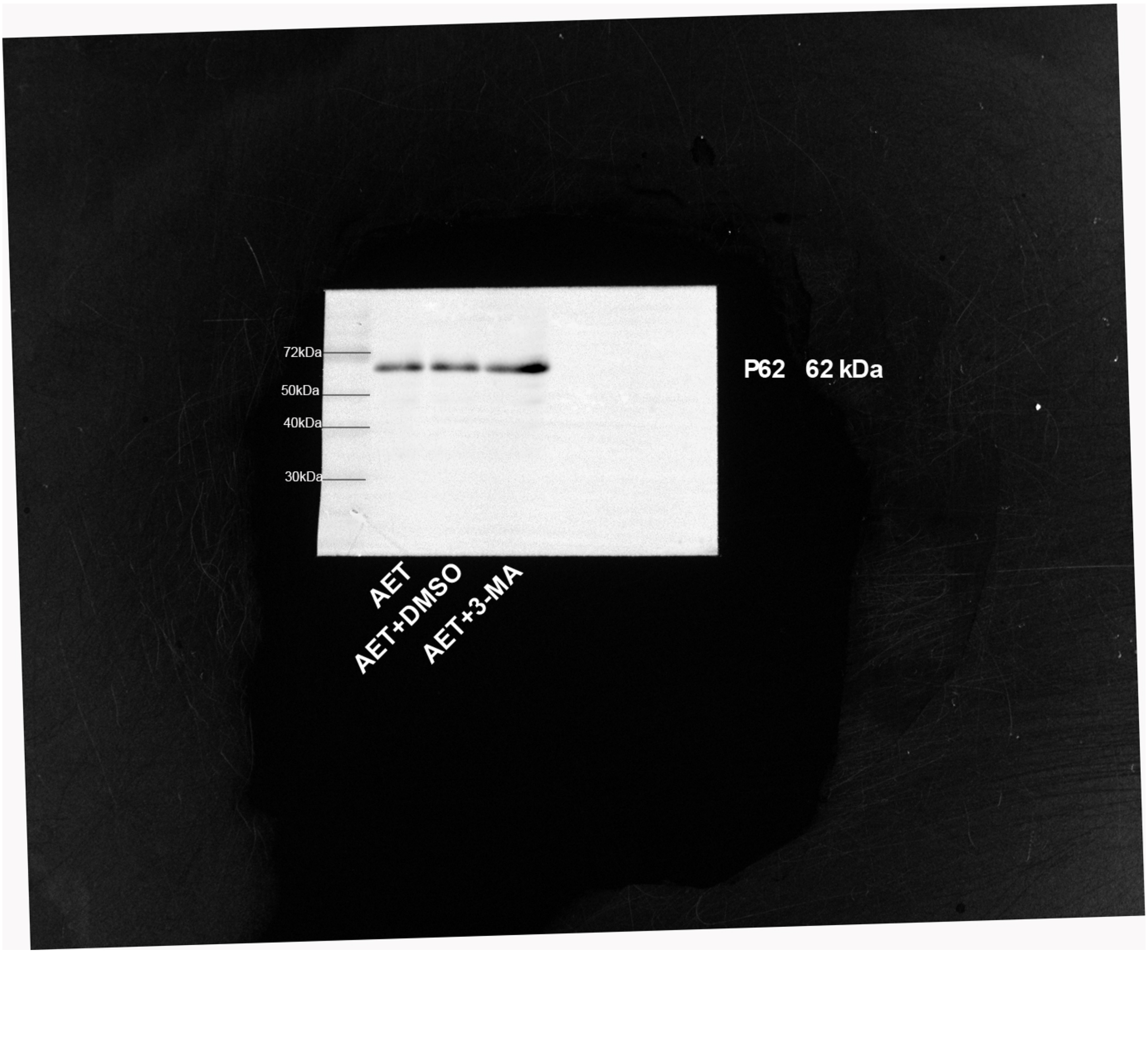

Full unedited gel/blot for Figure 4A

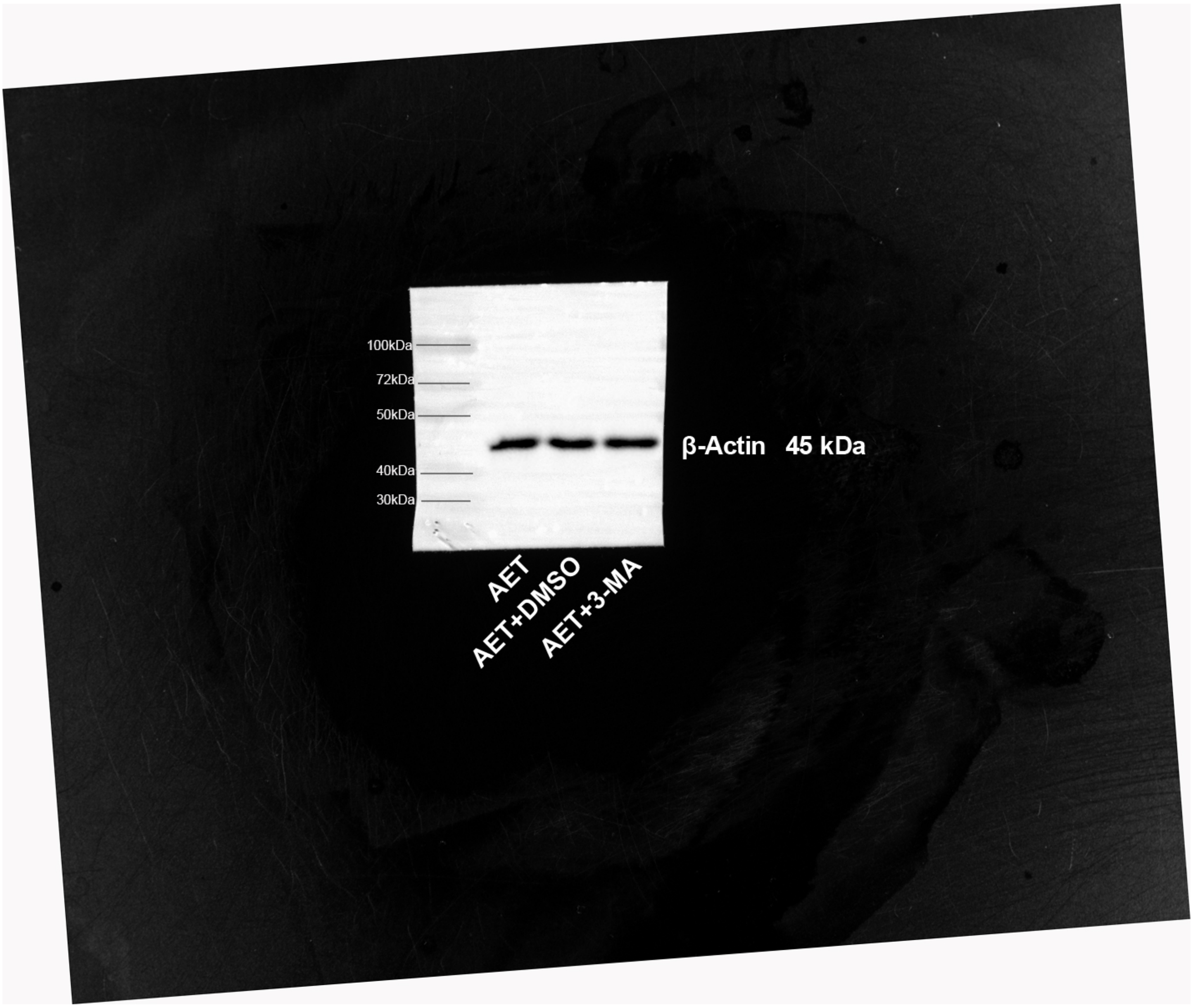

Full unedited gel/blot for Figure 4E

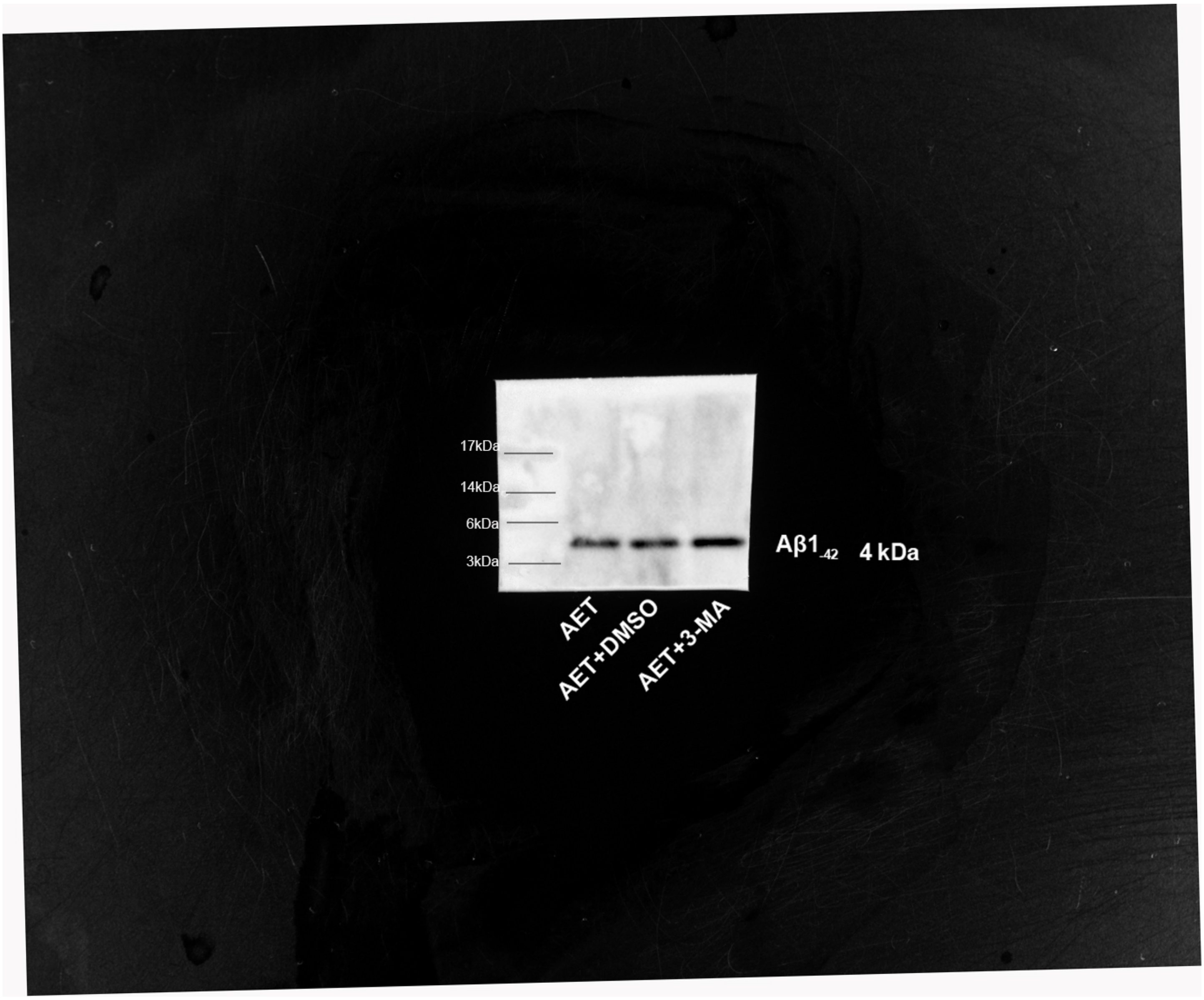

Full unedited gel/blot for Figure 4E

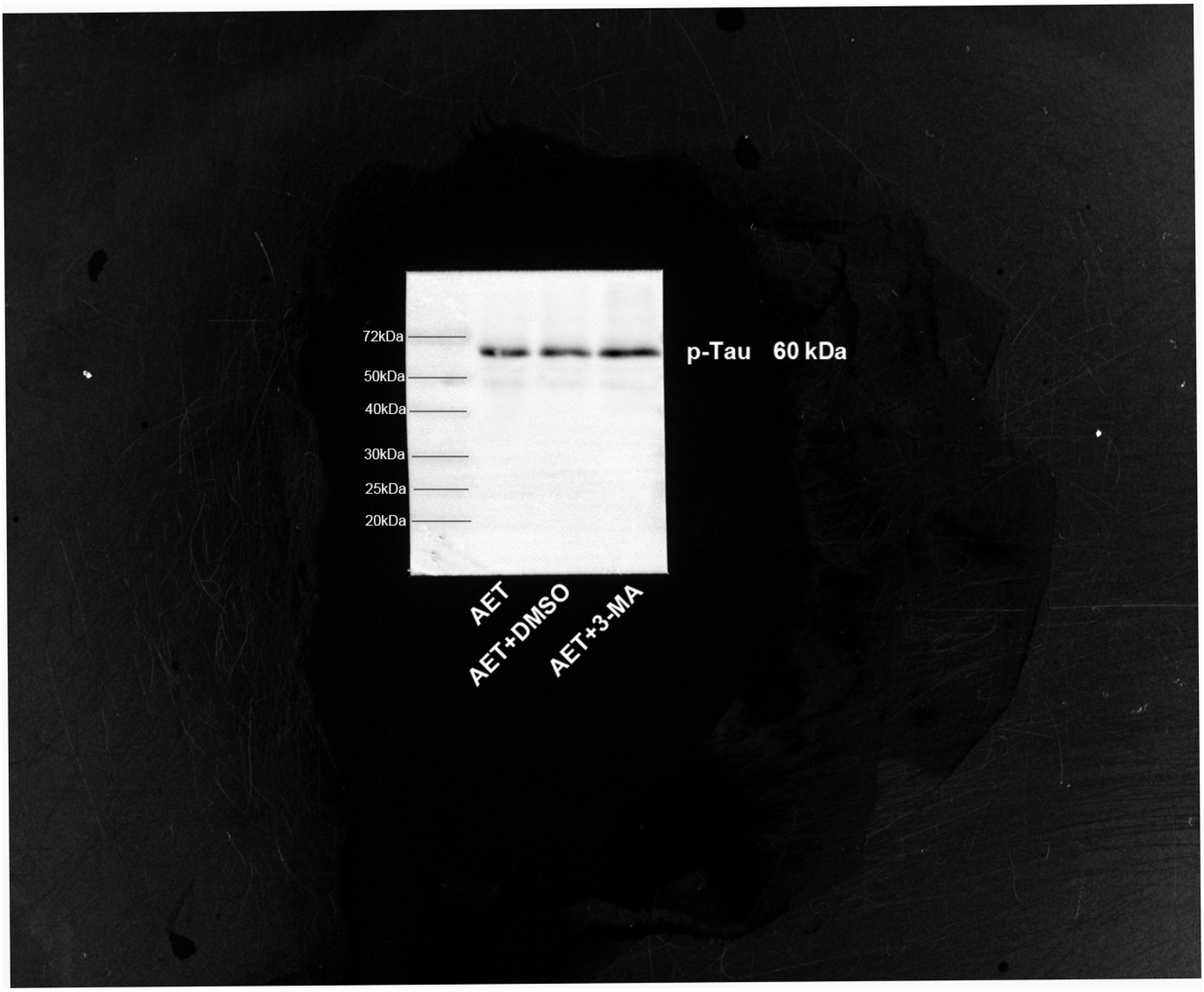

Full unedited gel/blot for Figure 4E

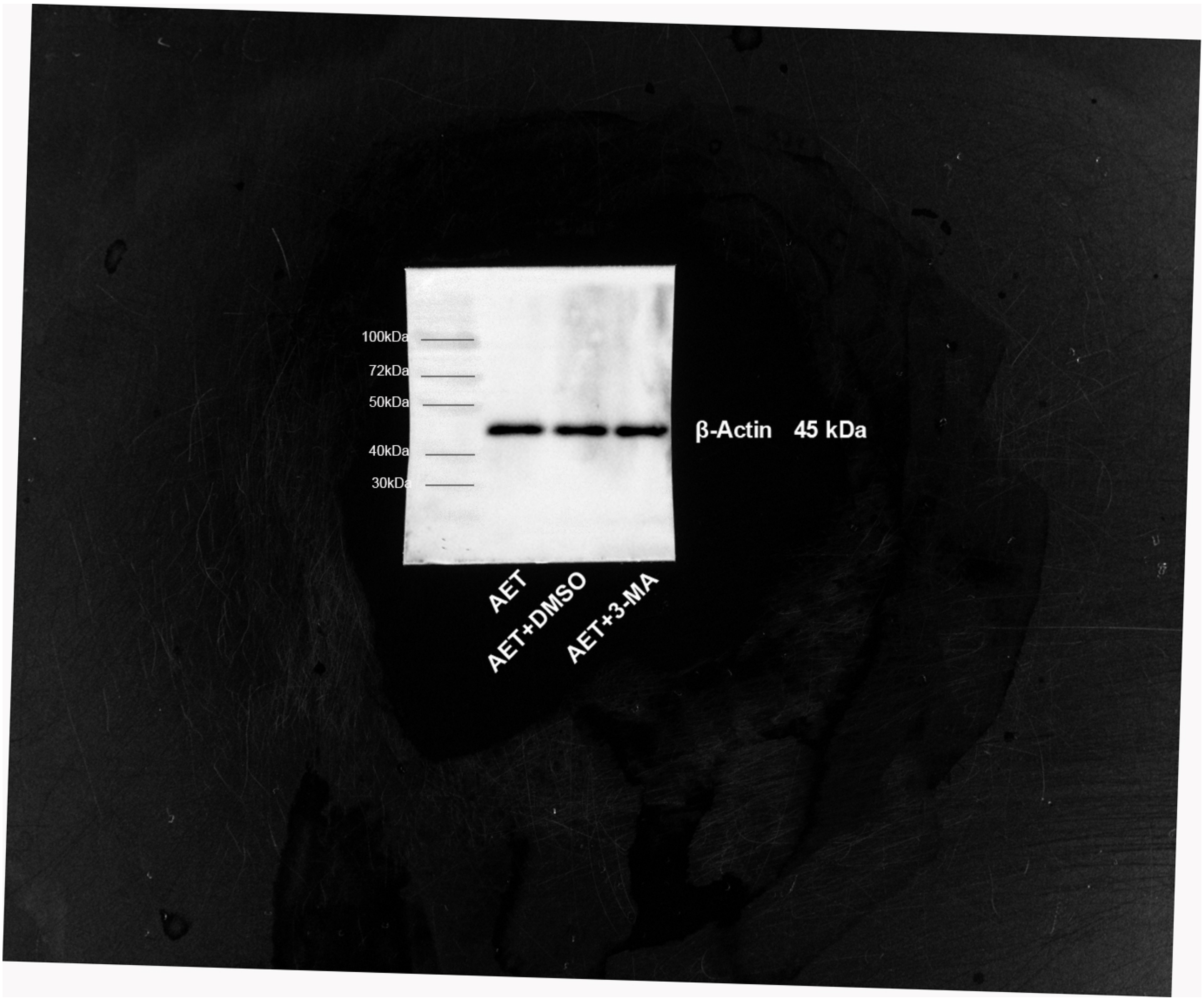

Full unedited gel/blot for Figure 5A

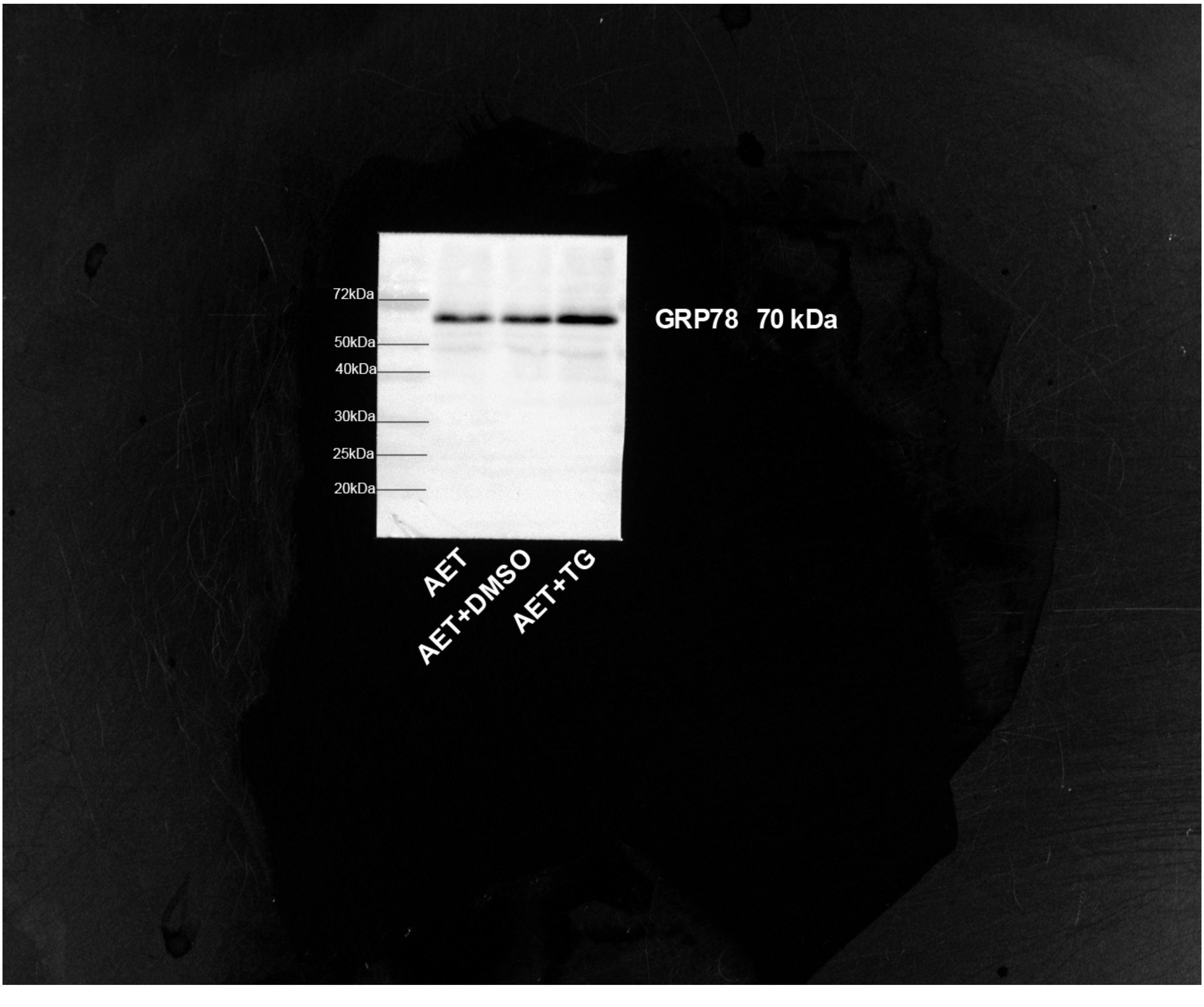

Full unedited gel/blot for Figure 5A

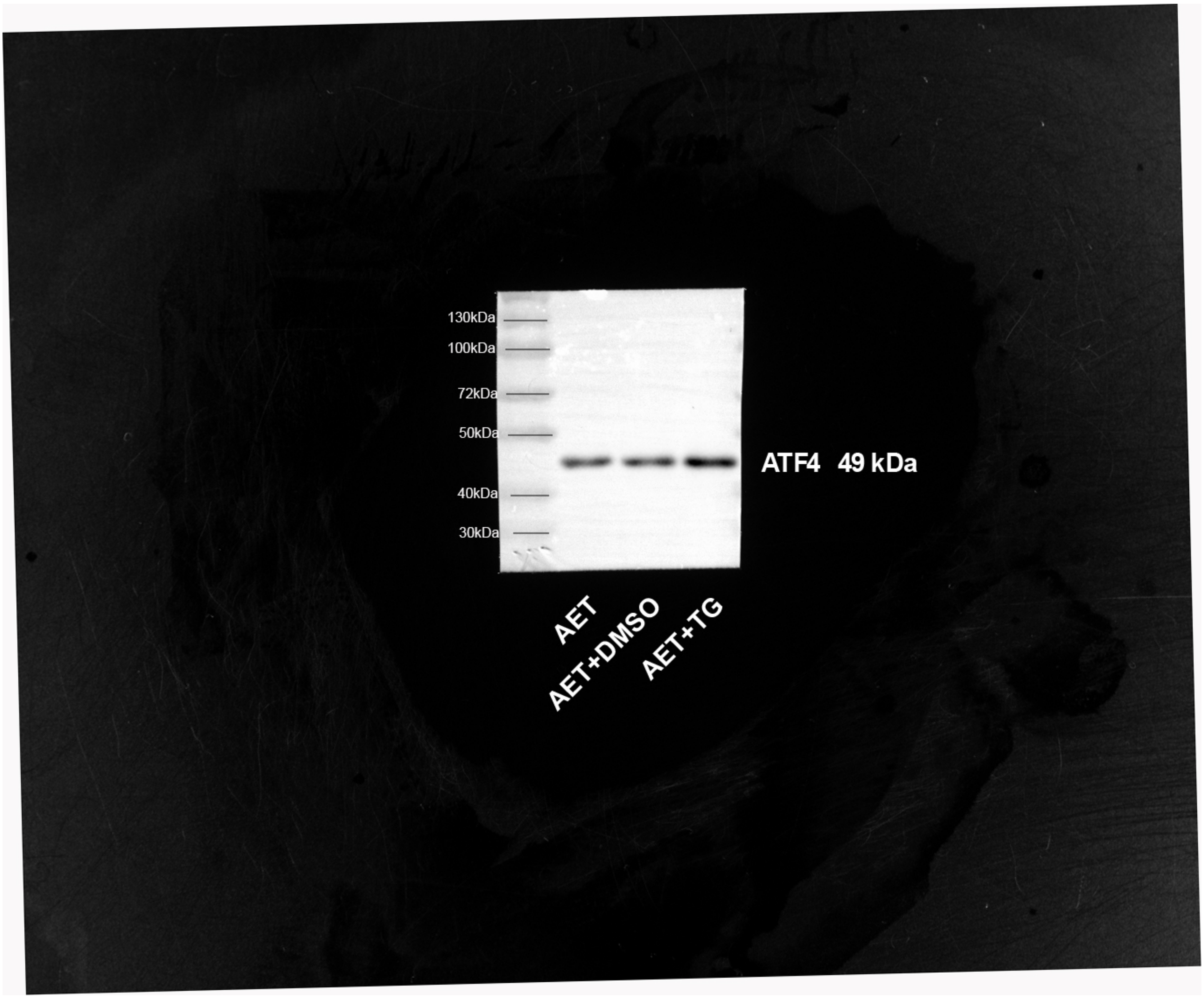

Full unedited gel/blot for Figure 5A

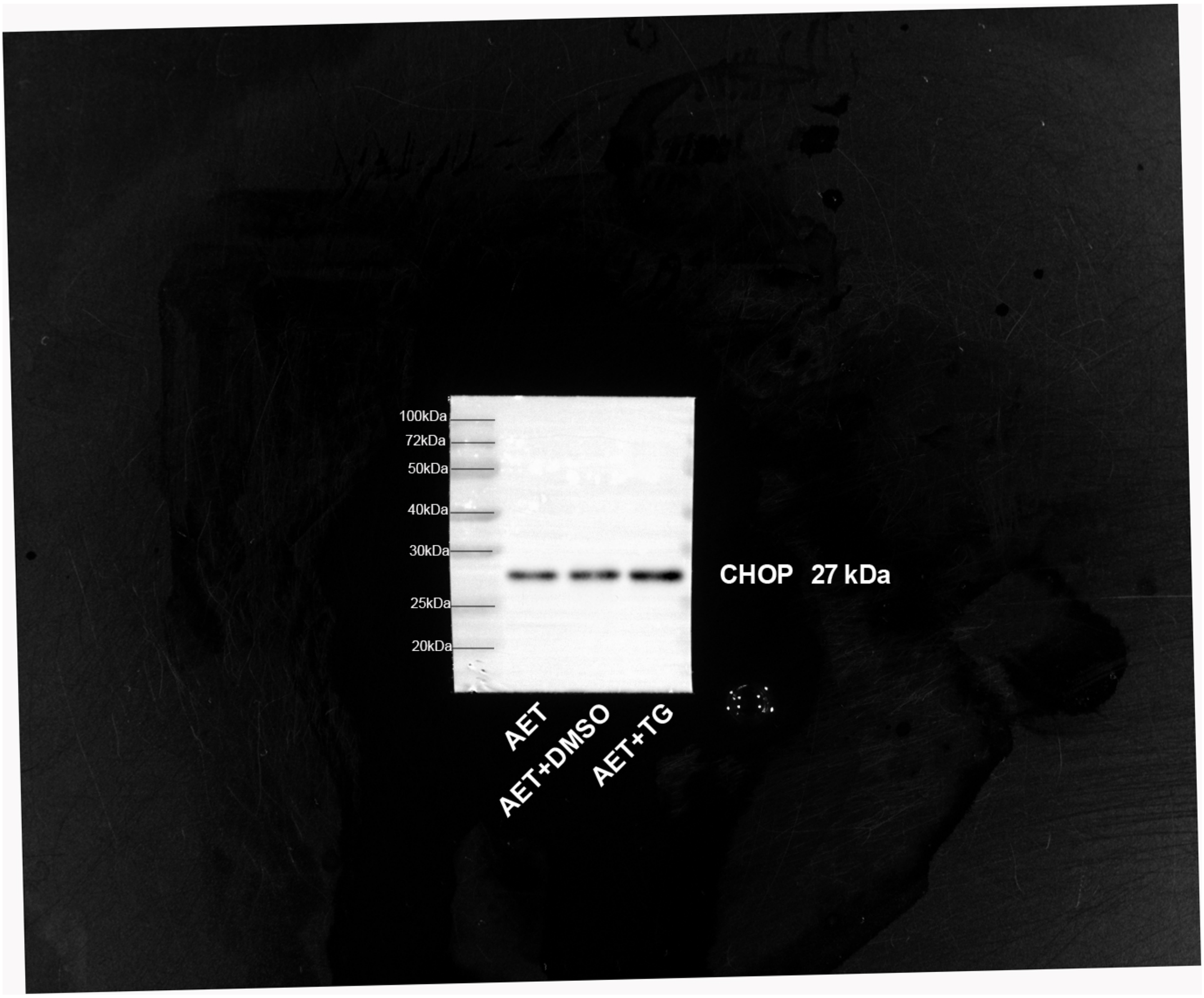

Full unedited gel/blot for Figure 5A

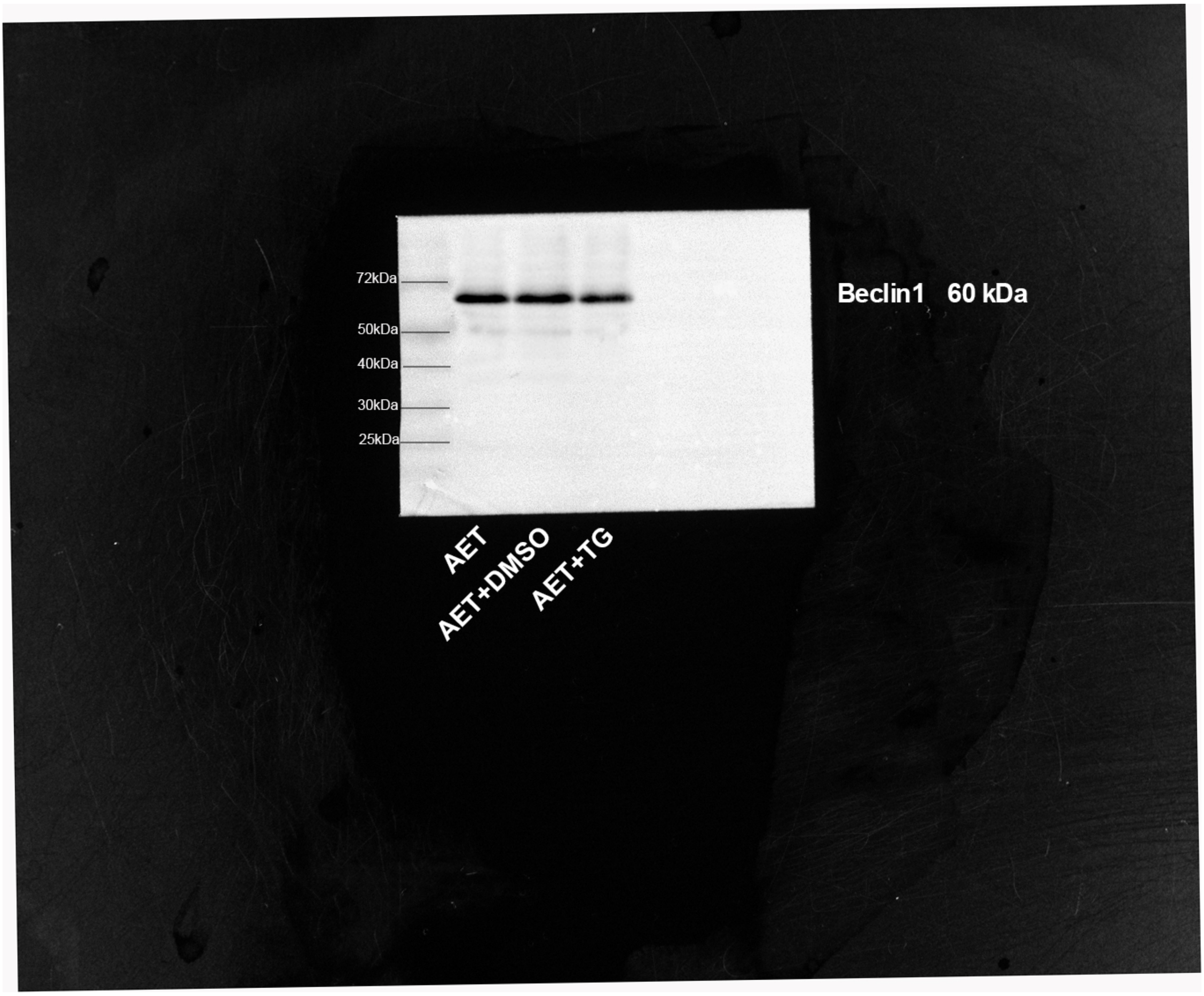

Full unedited gel/blot for Figure 5A

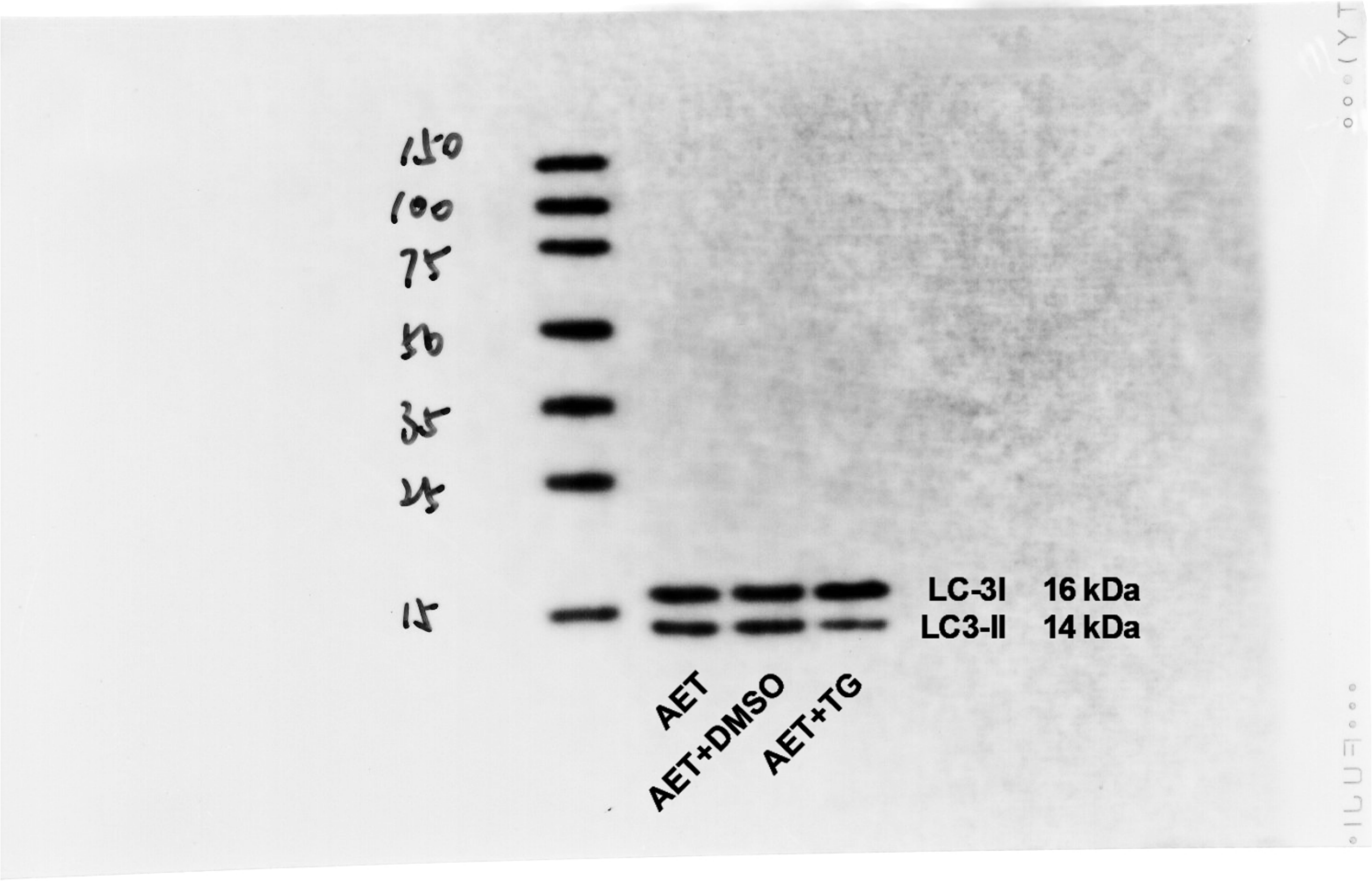

Full unedited gel/blot for Figure 5A

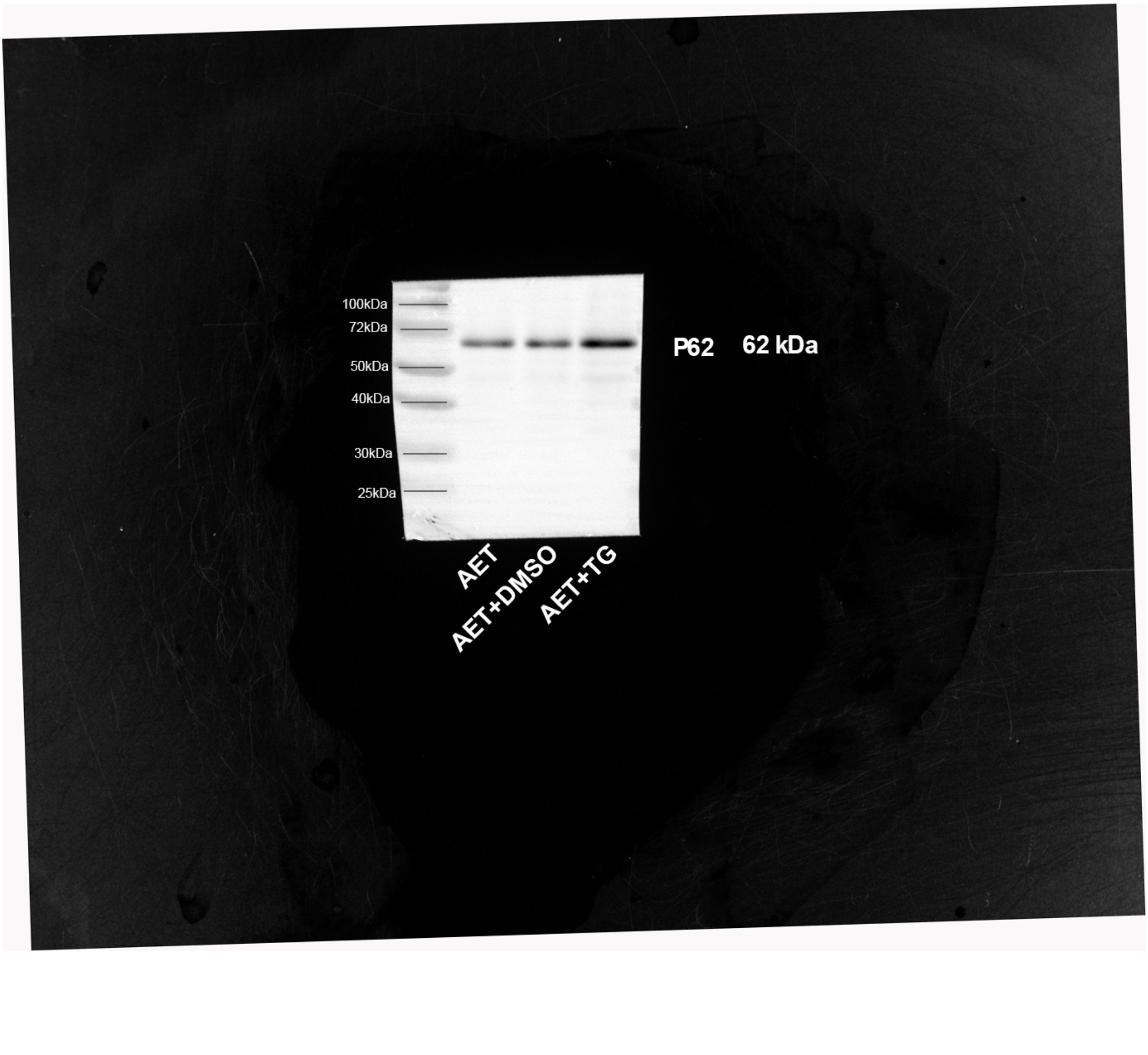

Full unedited gel/blot for Figure 5A

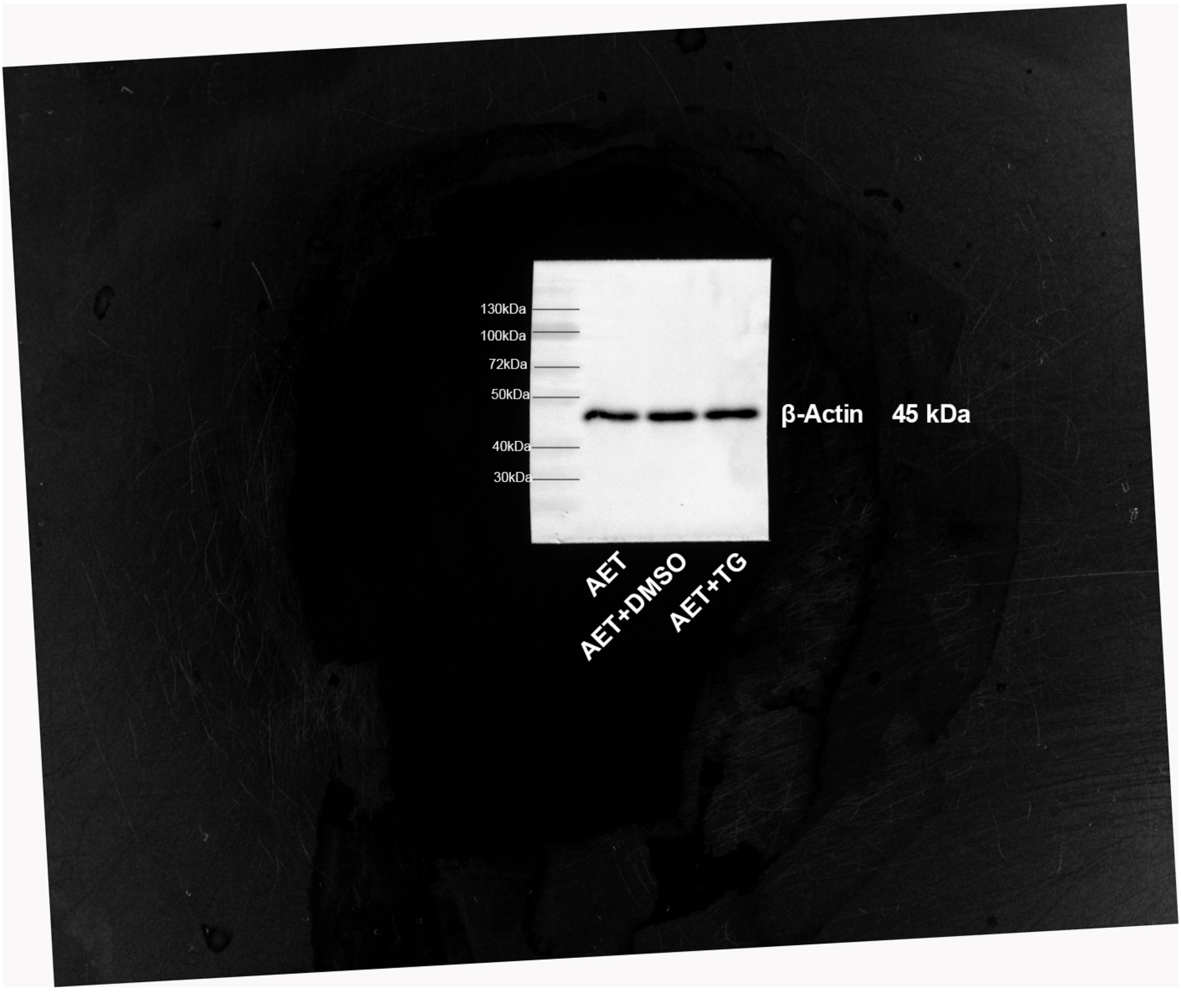

Full unedited gel/blot for Figure 5E

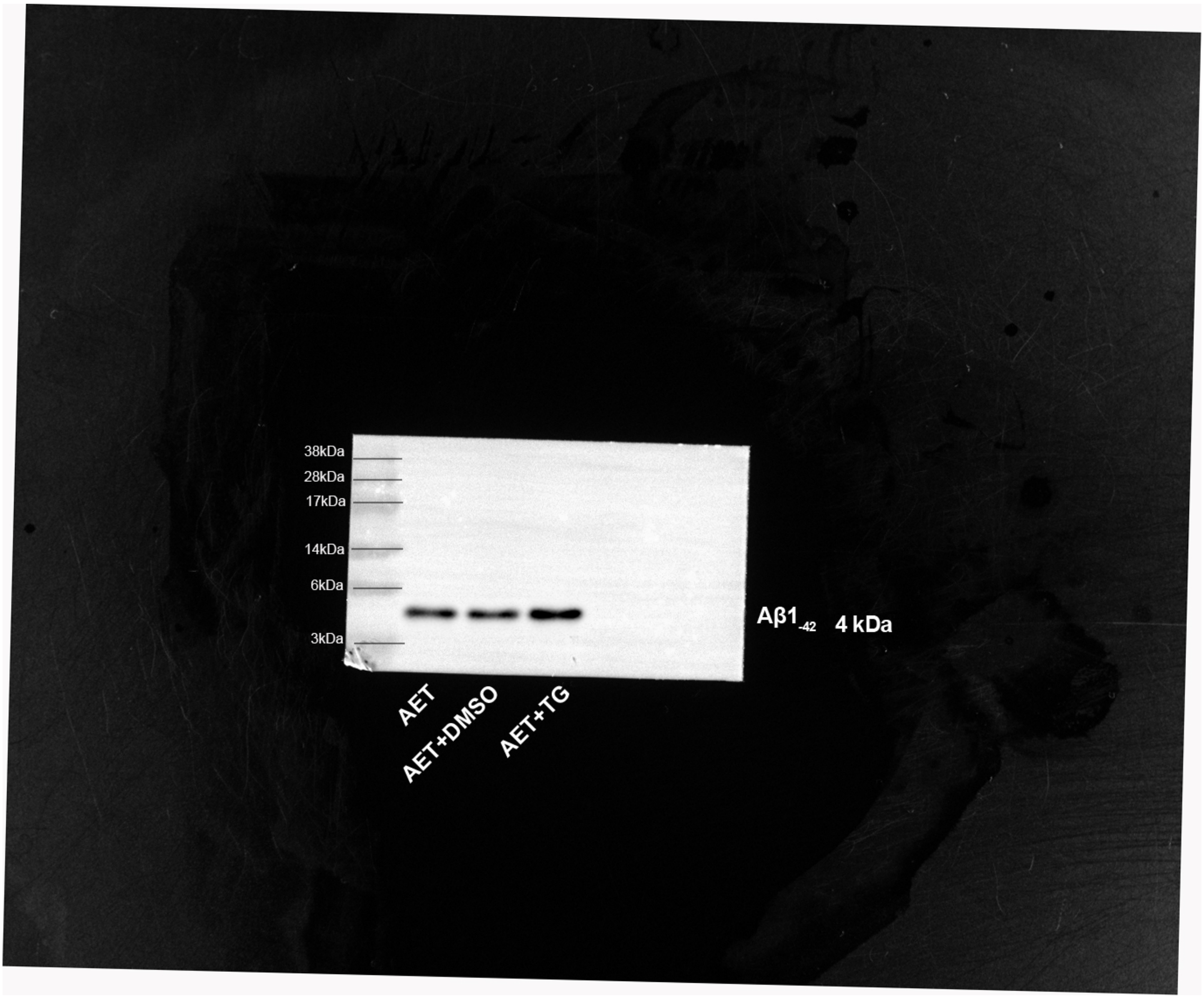

Full unedited gel/blot for Figure 5E

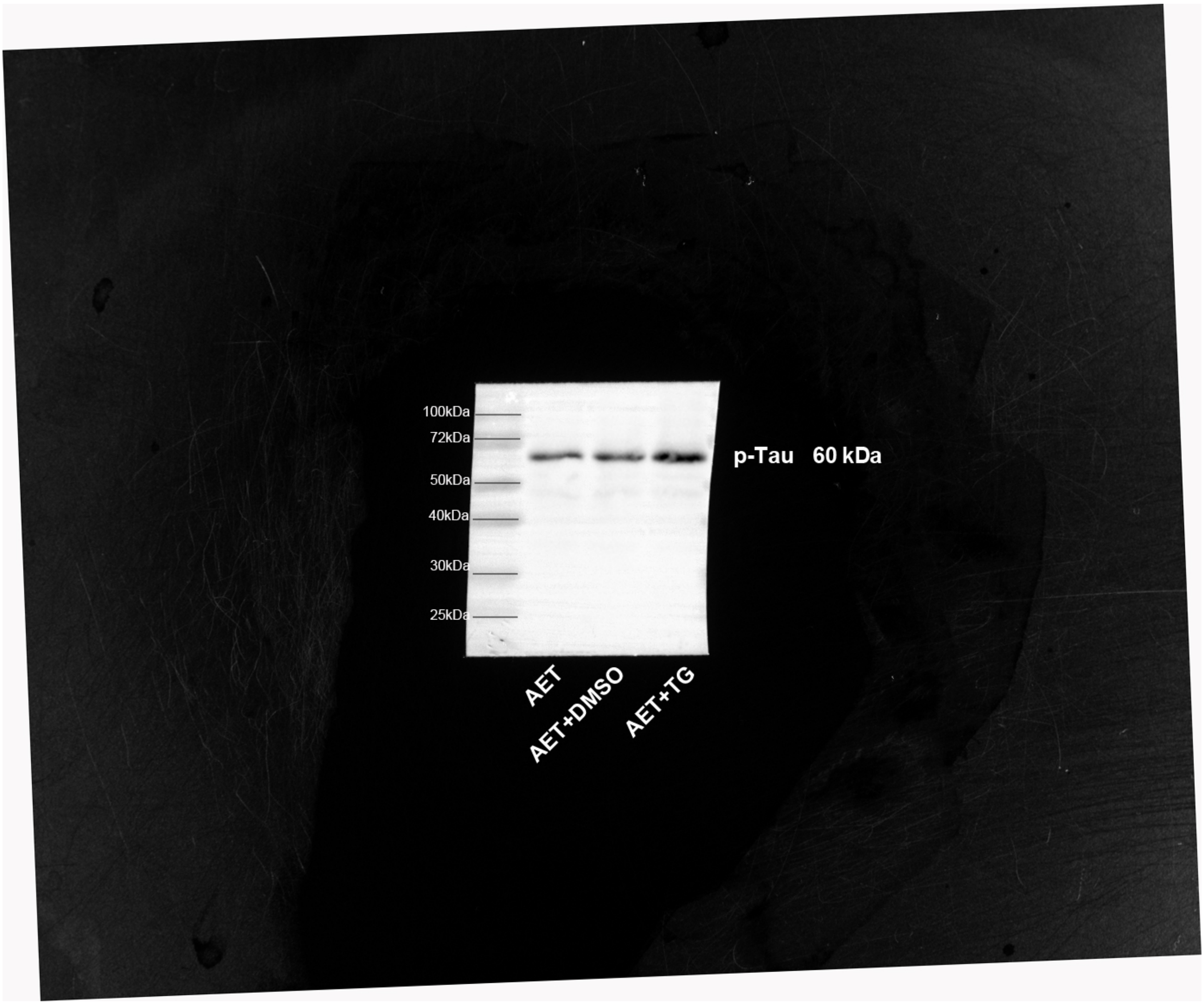

Full unedited gel/blot for Figure 5E

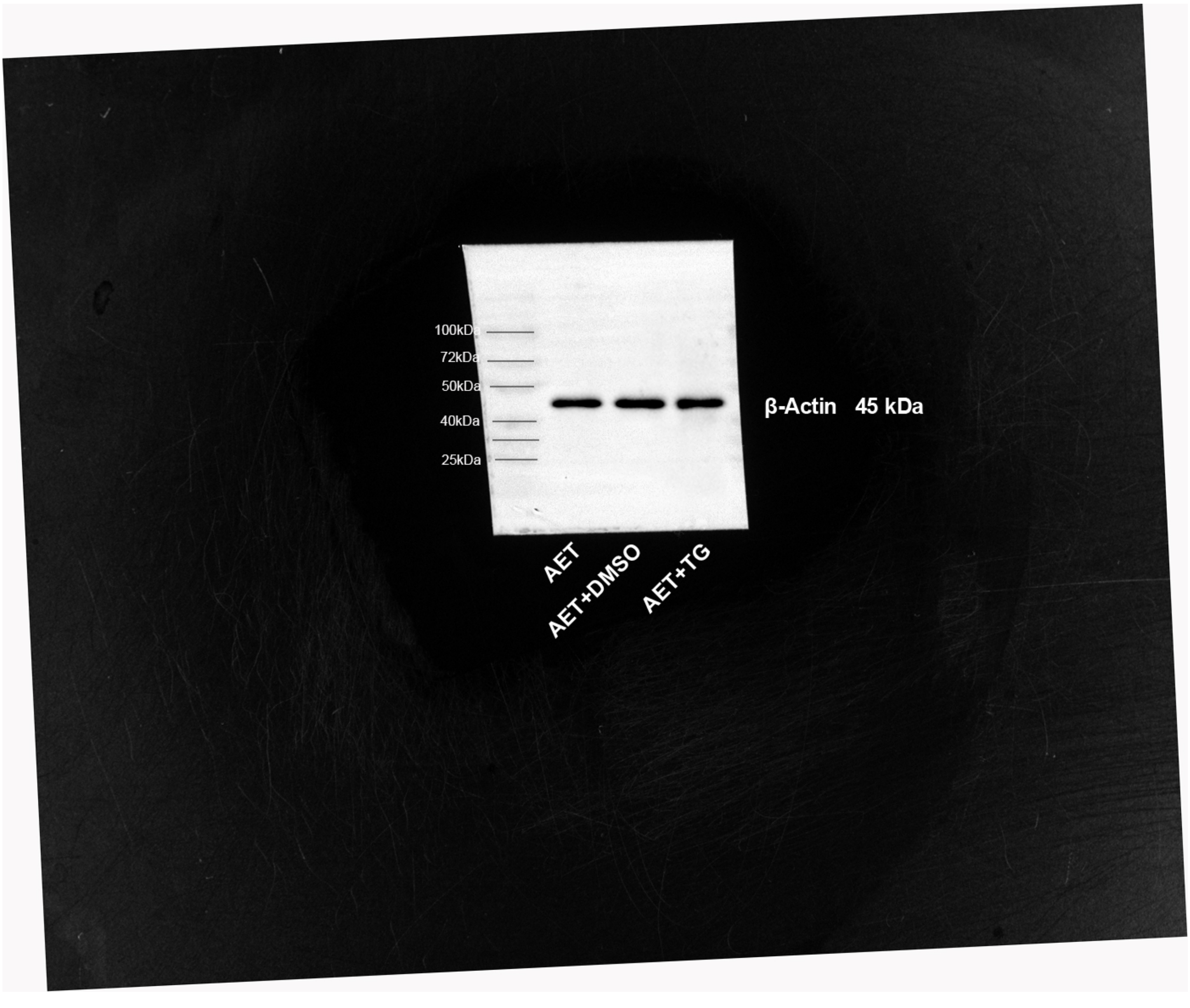

Full unedited gel/blot for Figure 6A

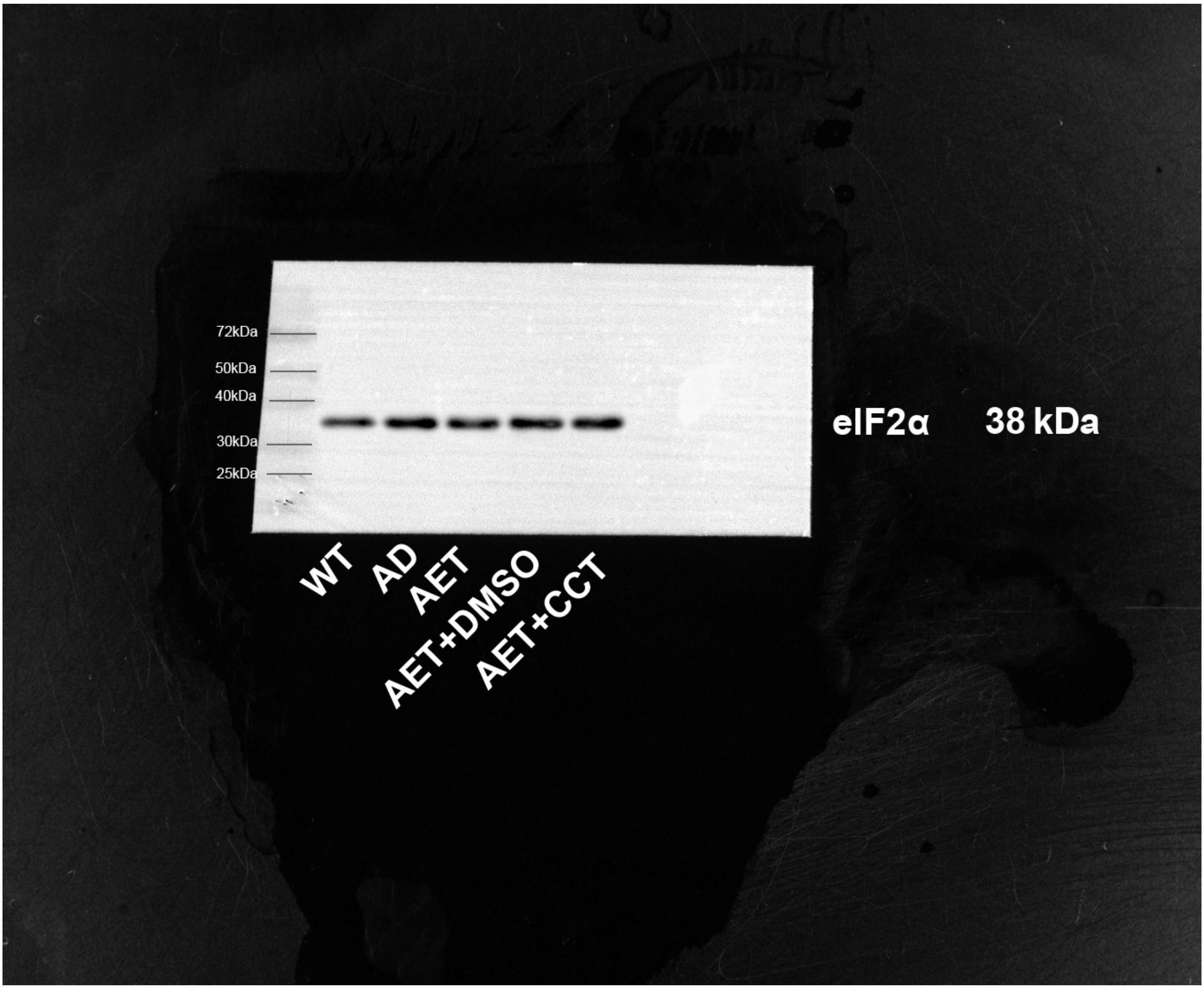

Full unedited gel/blot for Figure 6A

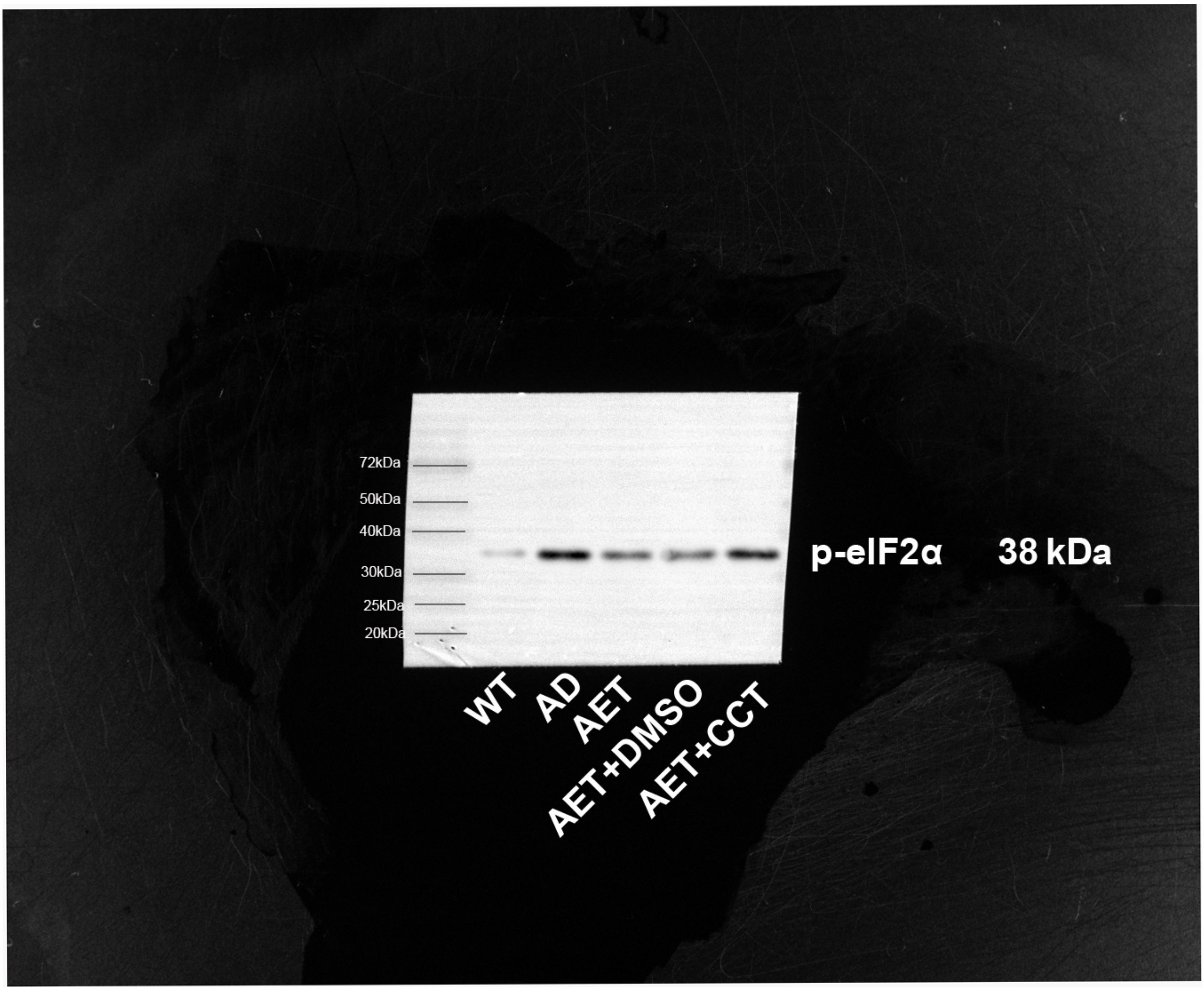

Full unedited gel/blot for Figure 6A

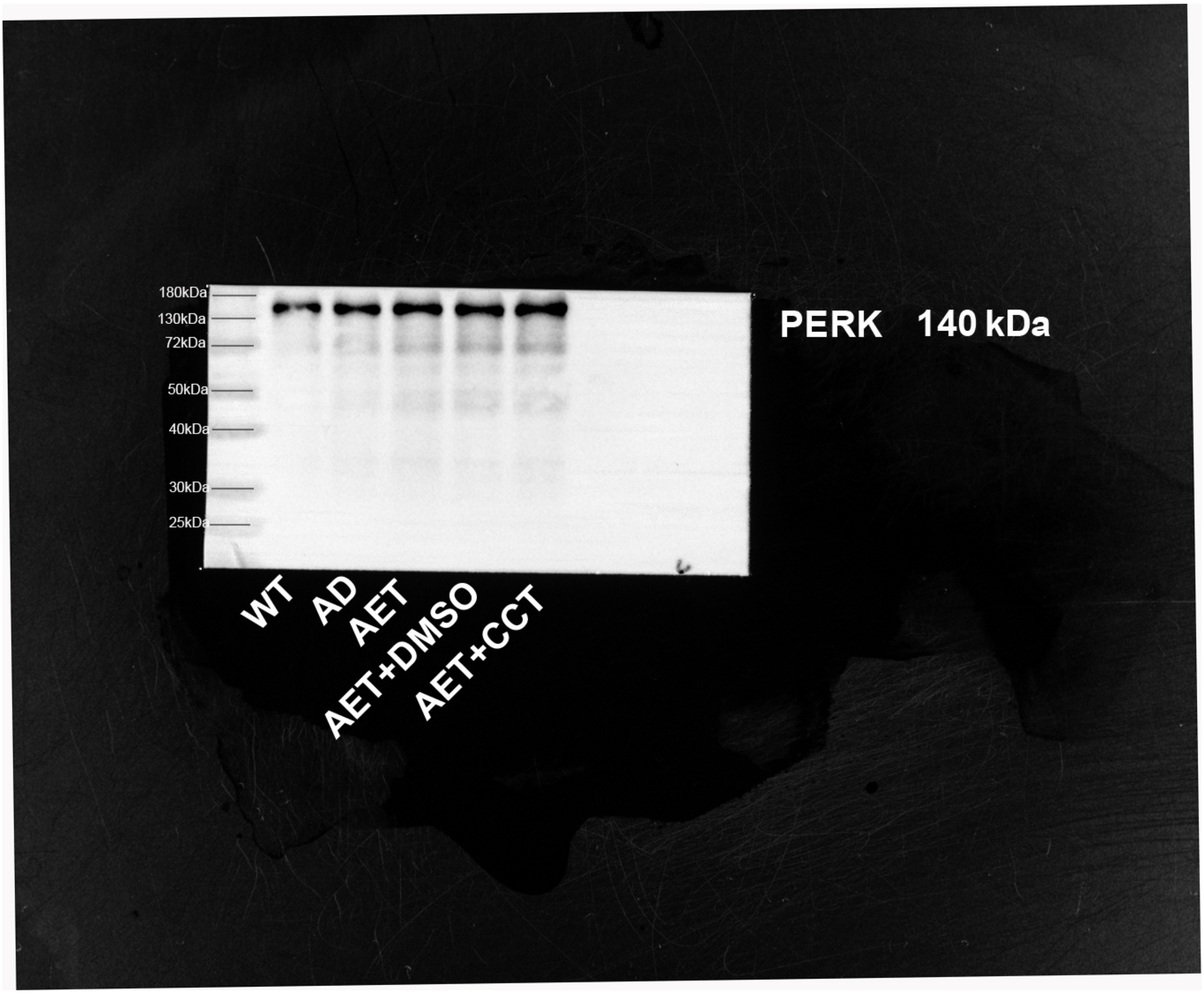

Full unedited gel/blot for Figure 6A

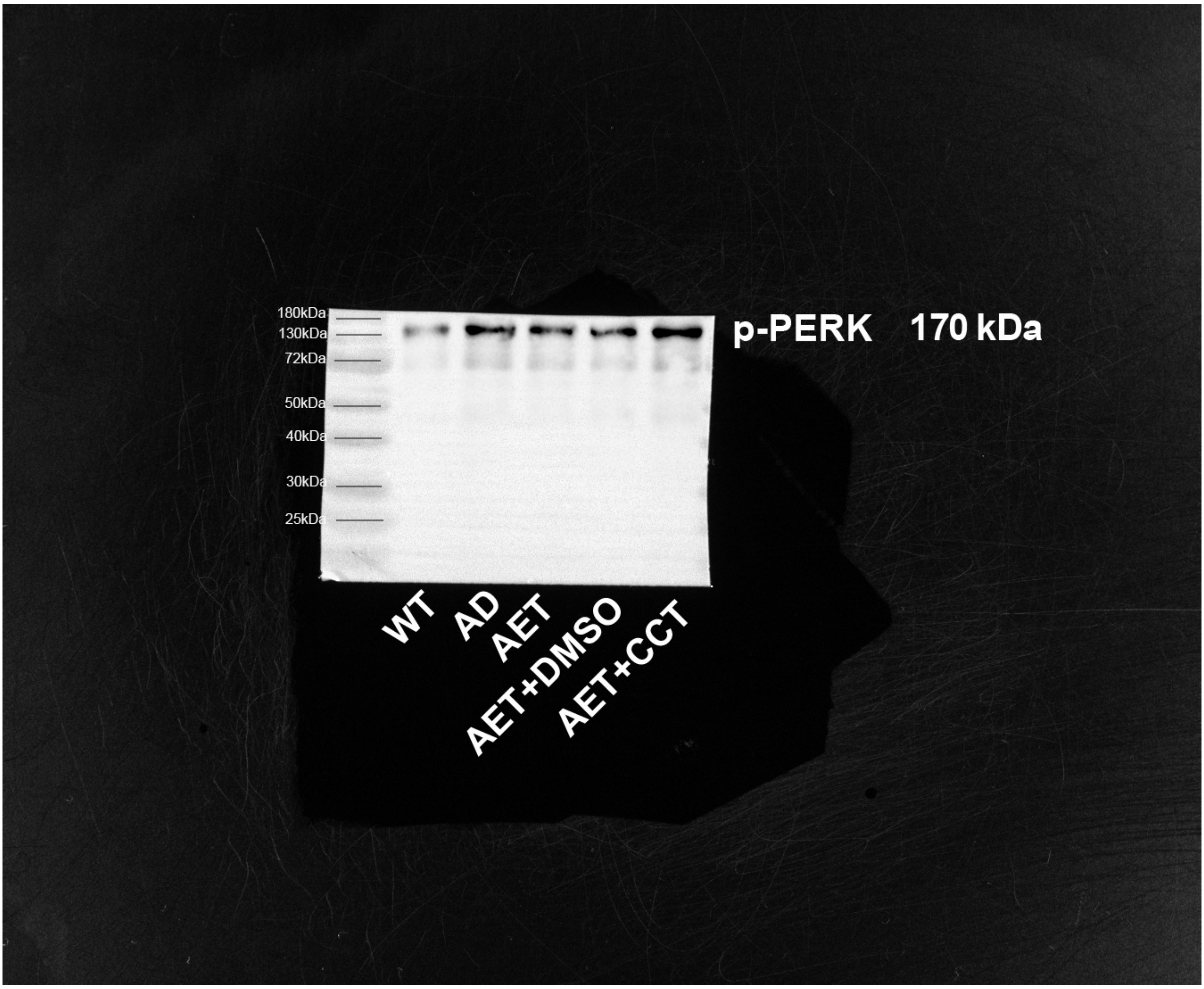

Full unedited gel/blot for Figure 6A

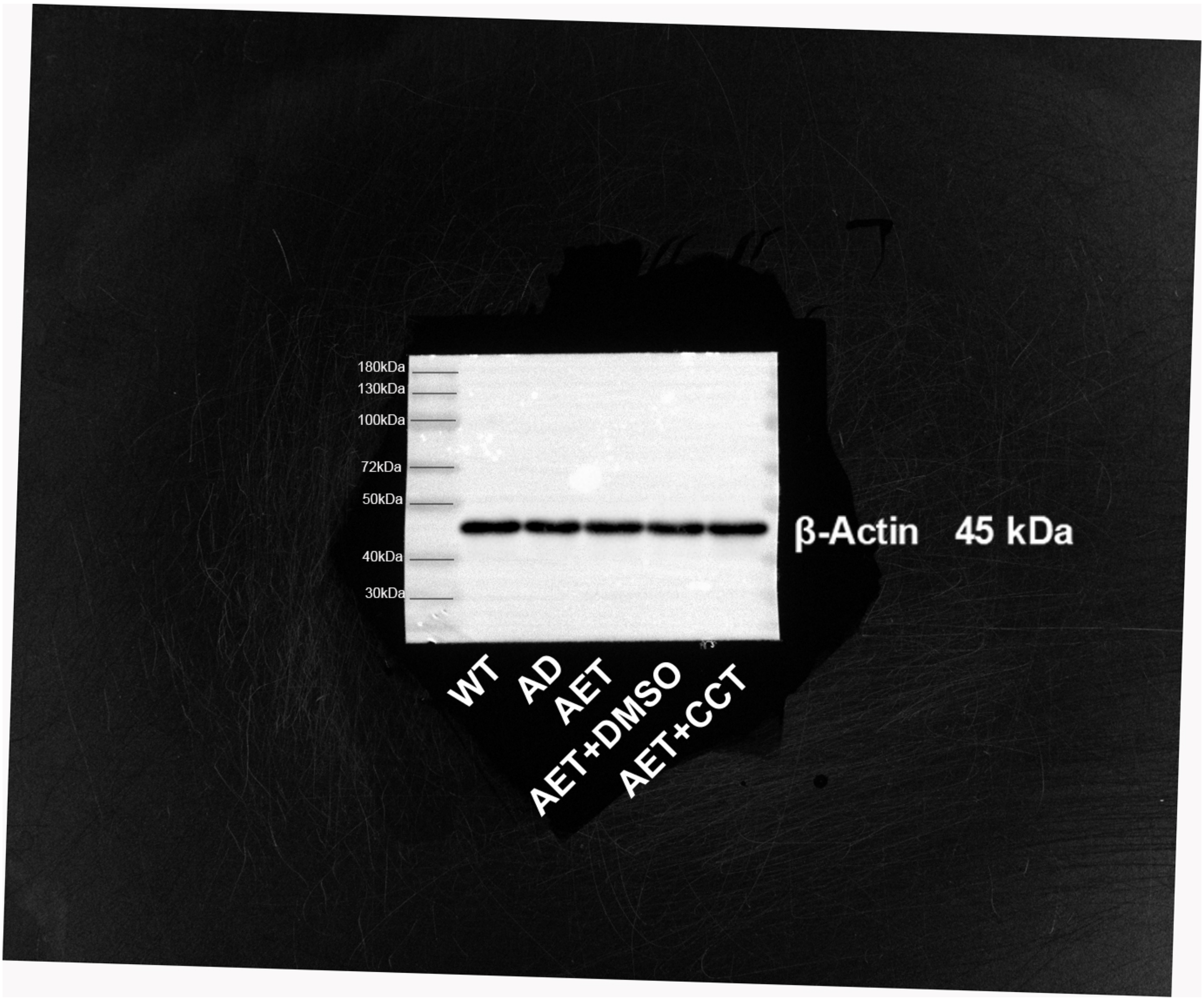

Full unedited gel/blot for Figure 6B

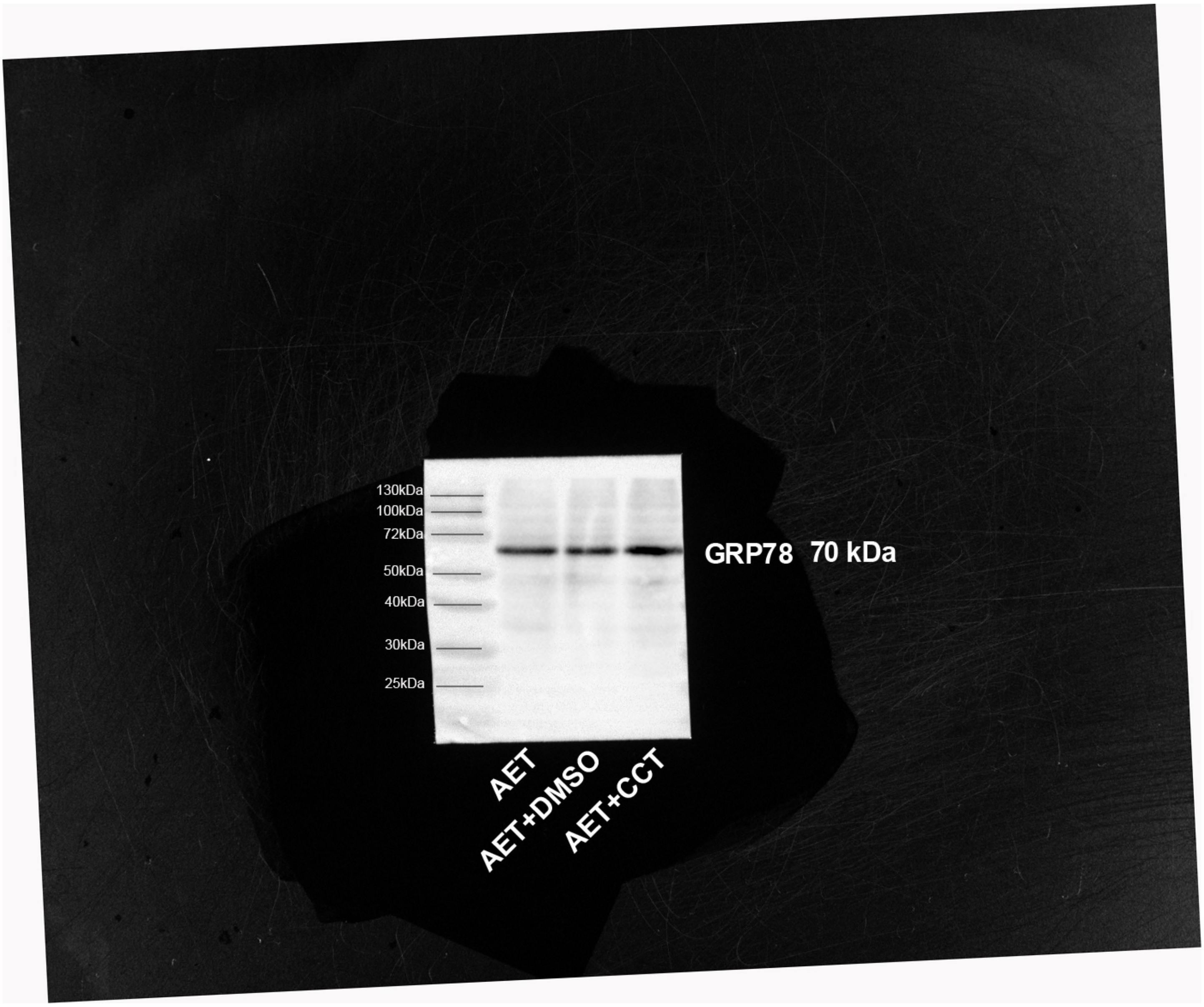

Full unedited gel/blot for Figure 6B

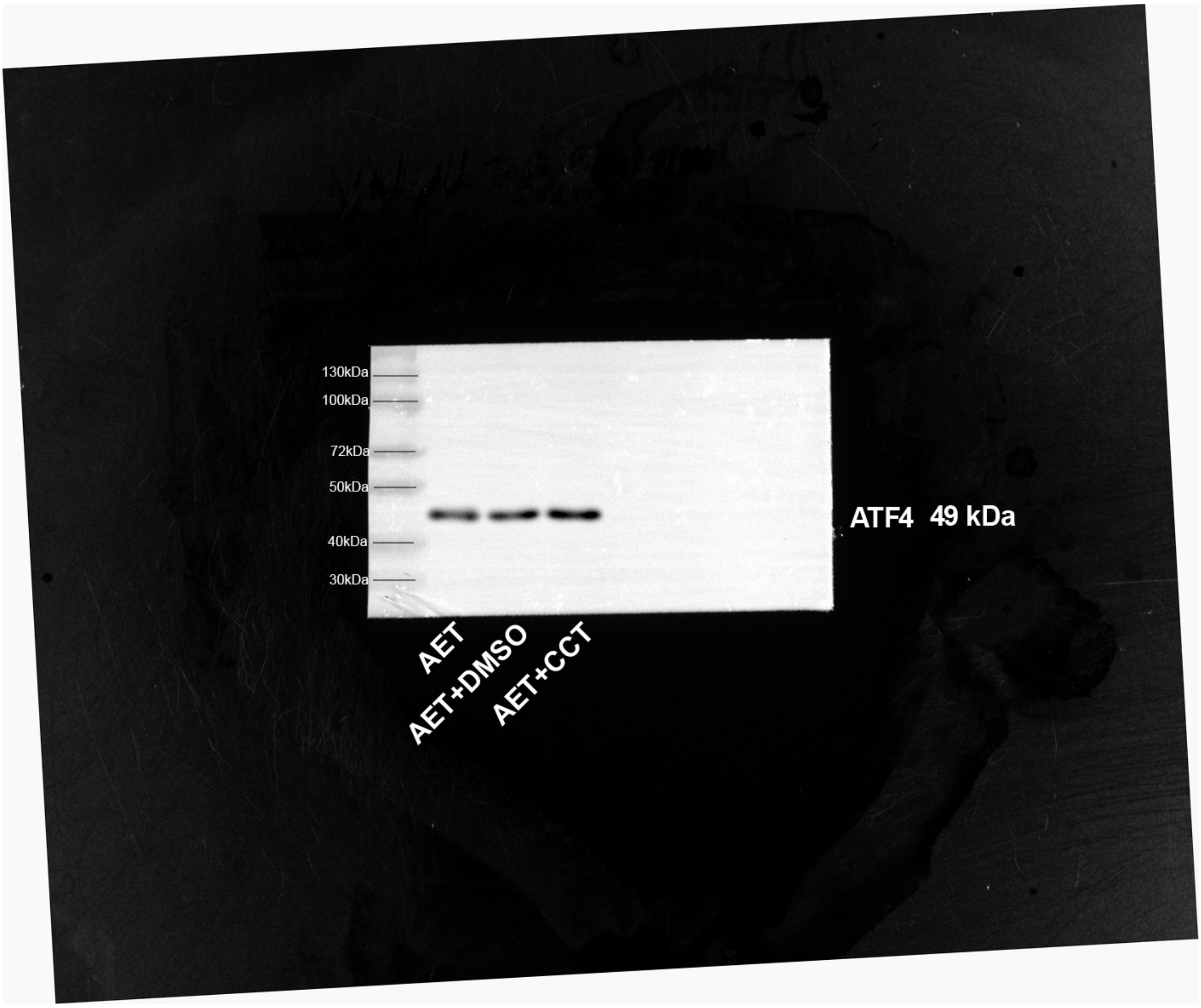

Full unedited gel/blot for Figure 6B

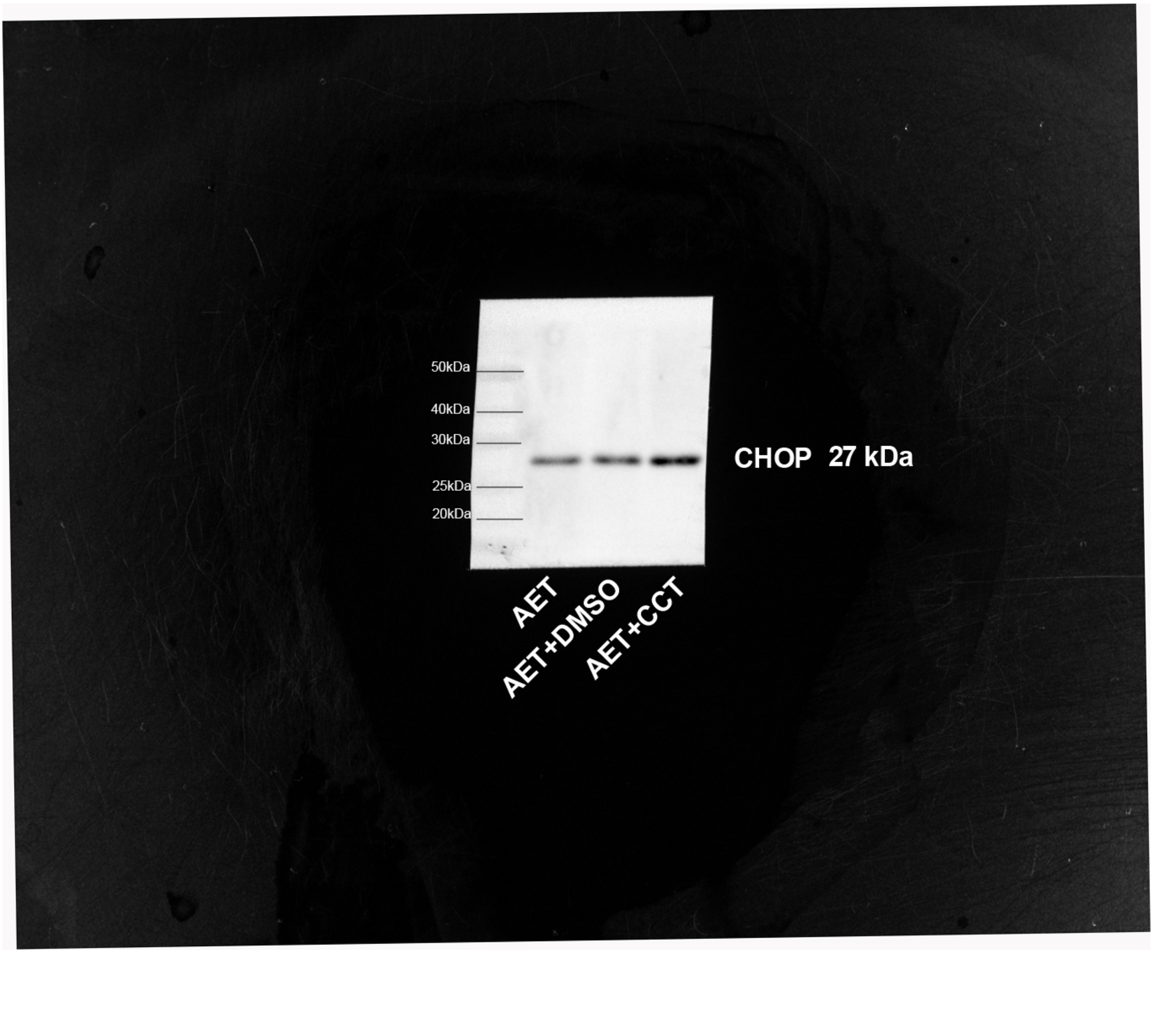

Full unedited gel/blot for Figure 6B

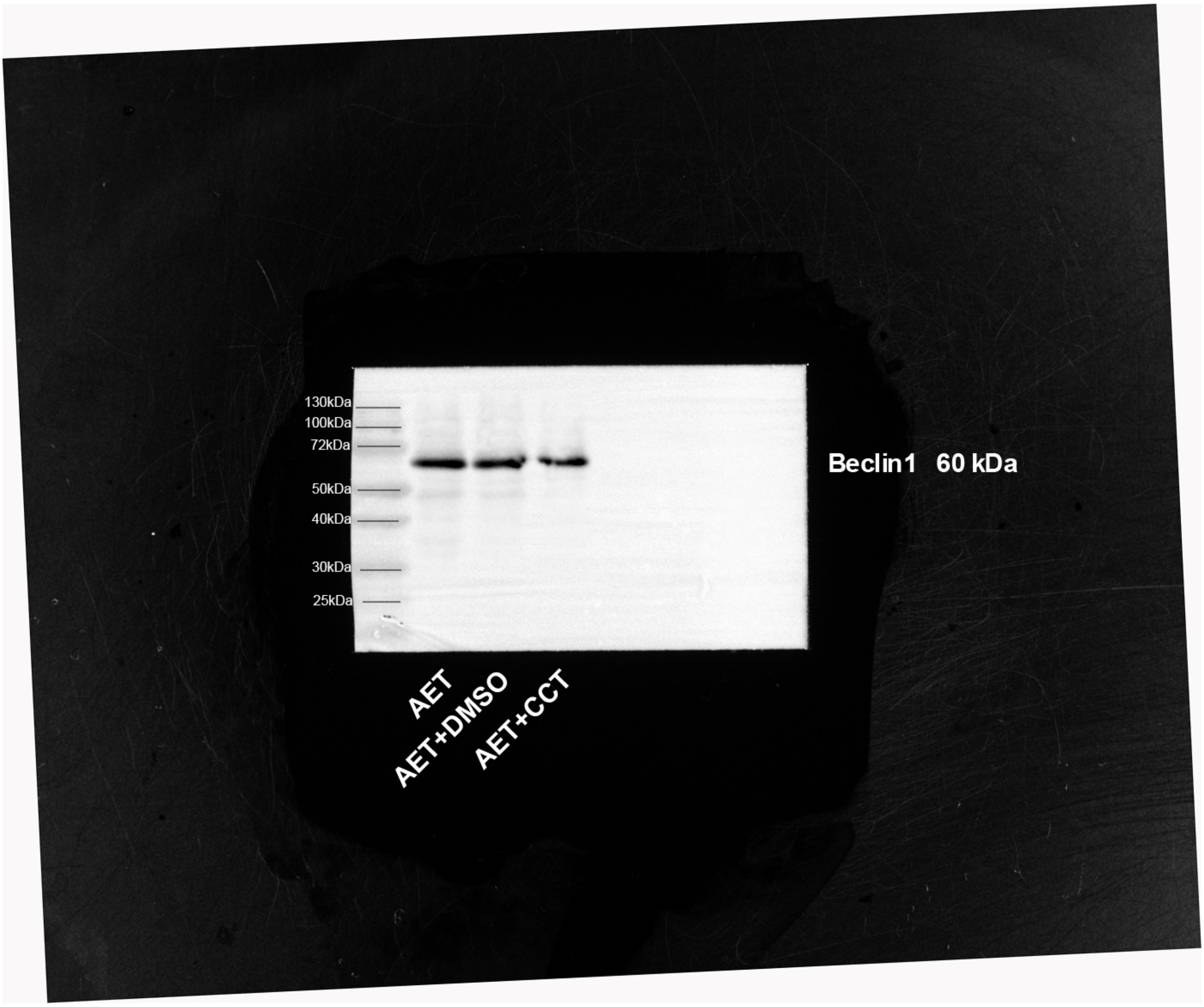

Full unedited gel/blot for Figure 6B

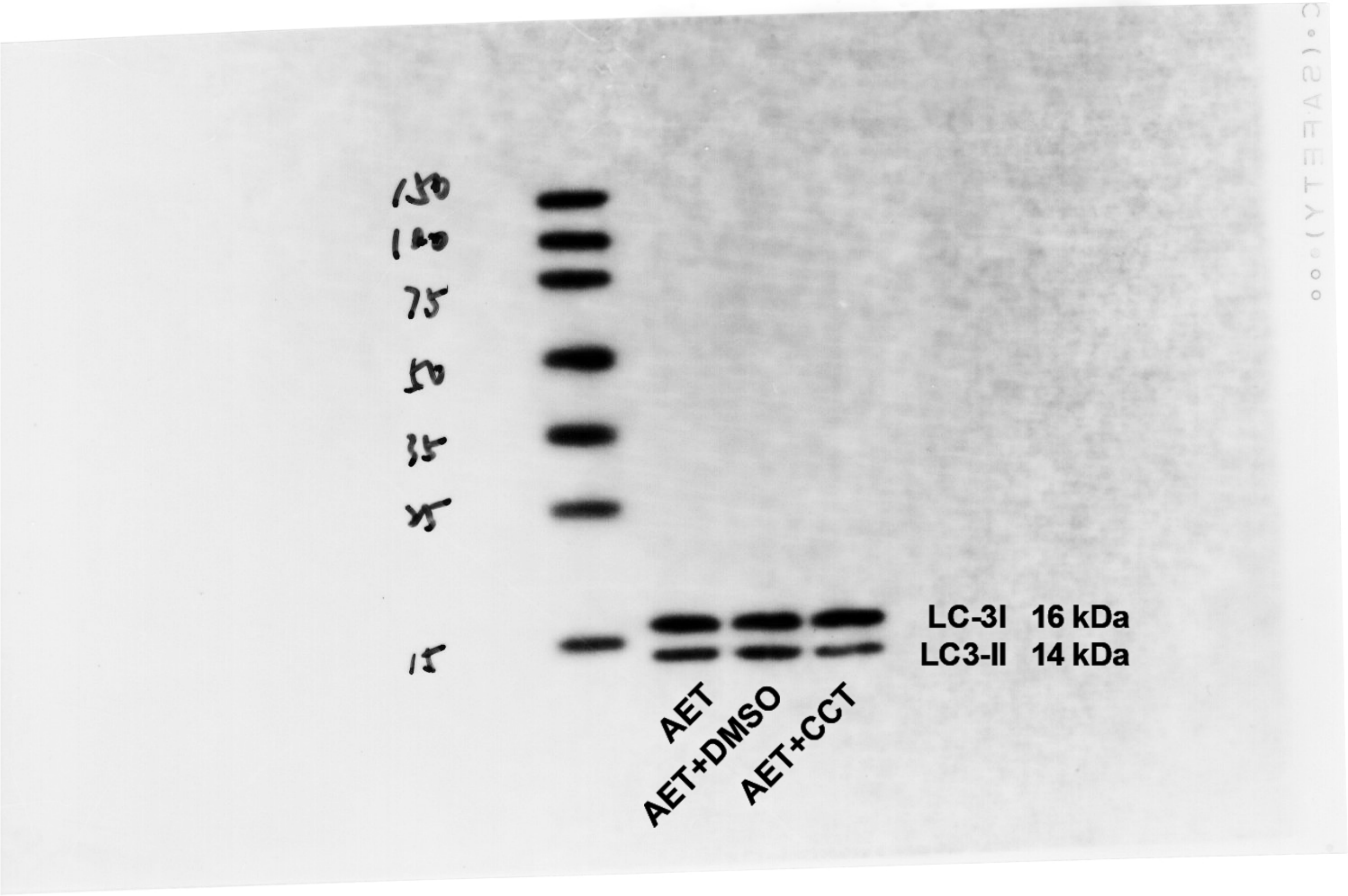

Full unedited gel/blot for Figure 6B

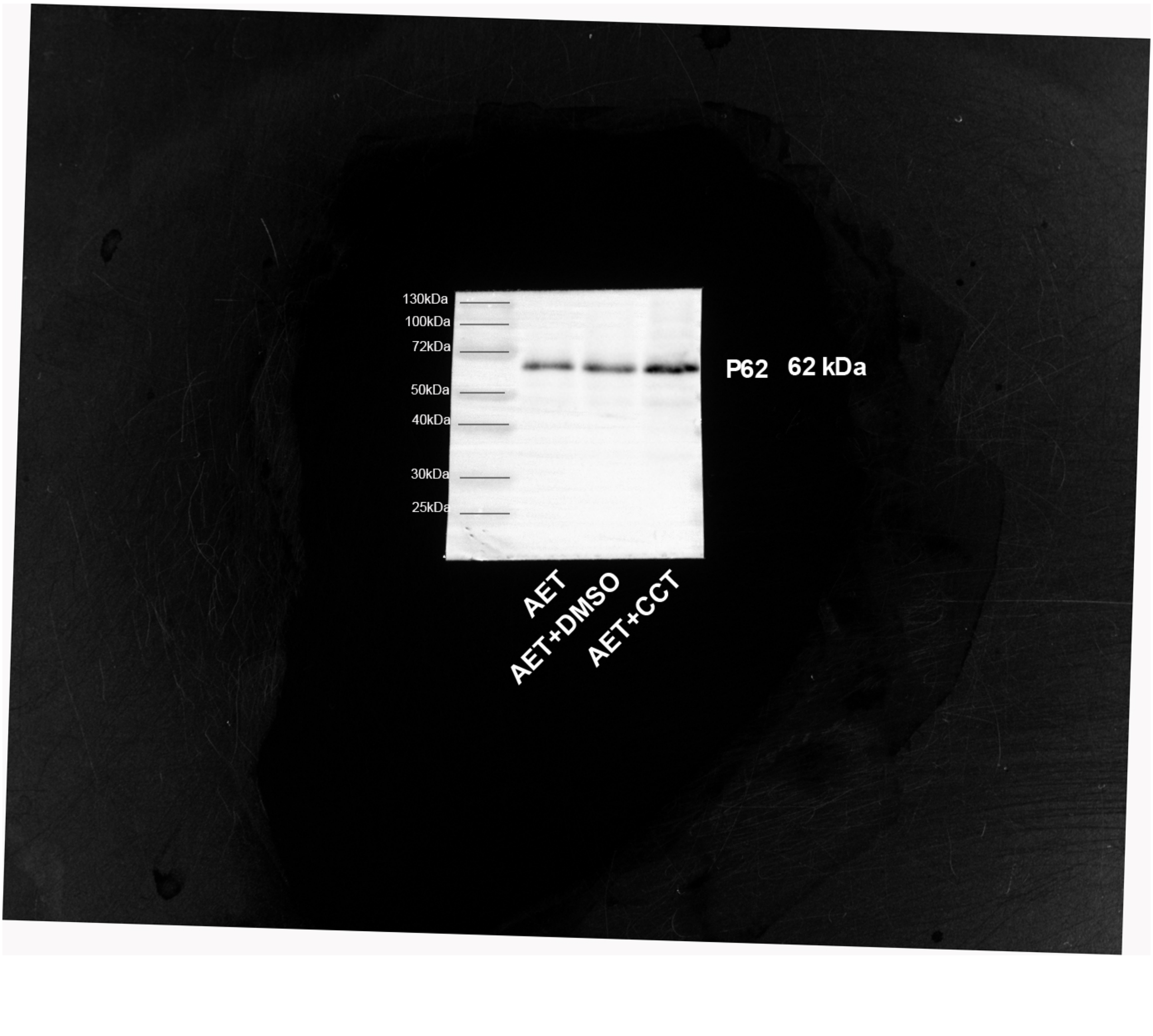

Full unedited gel/blot for Figure 6B

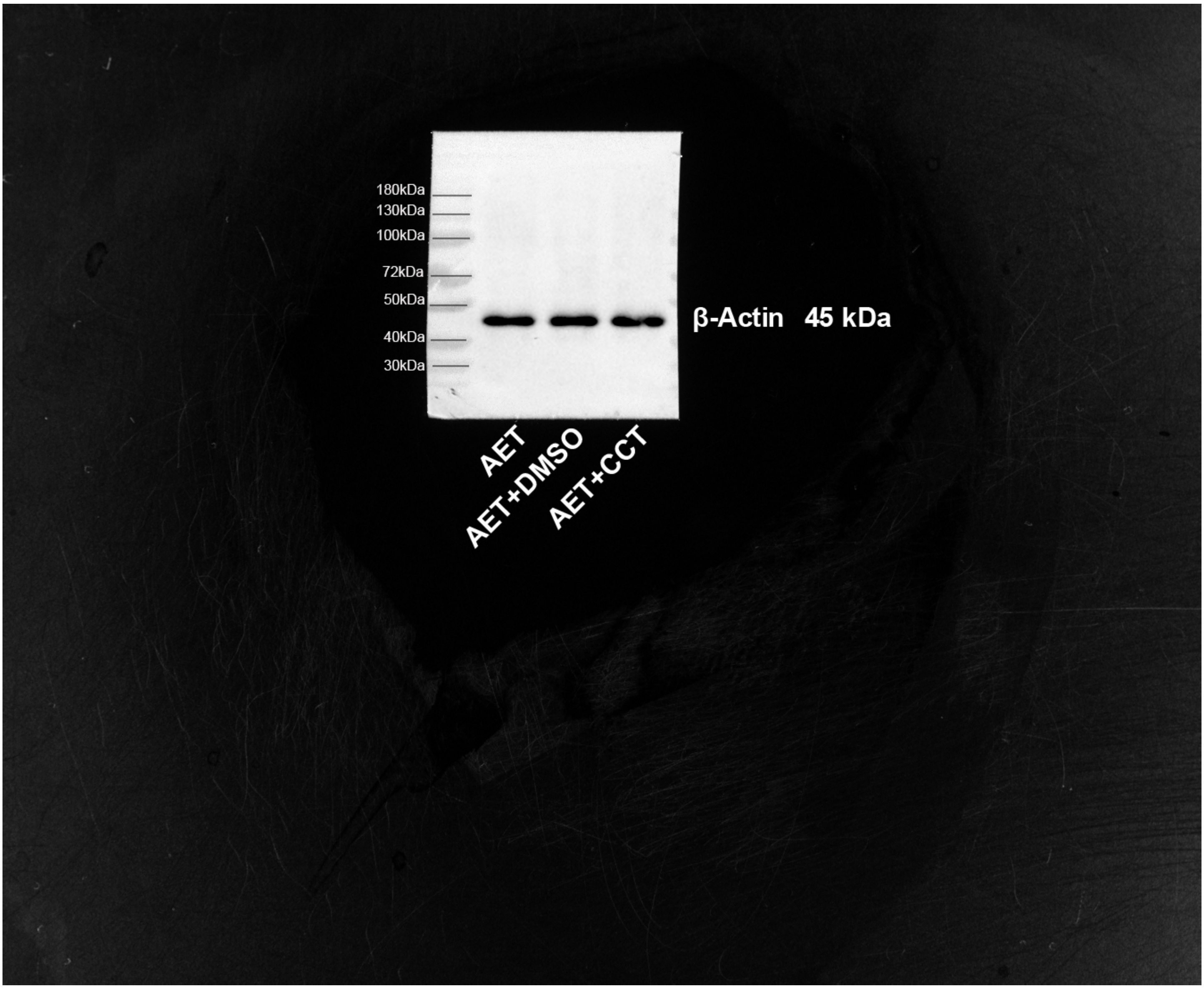

Full unedited gel/blot for Figure 6F

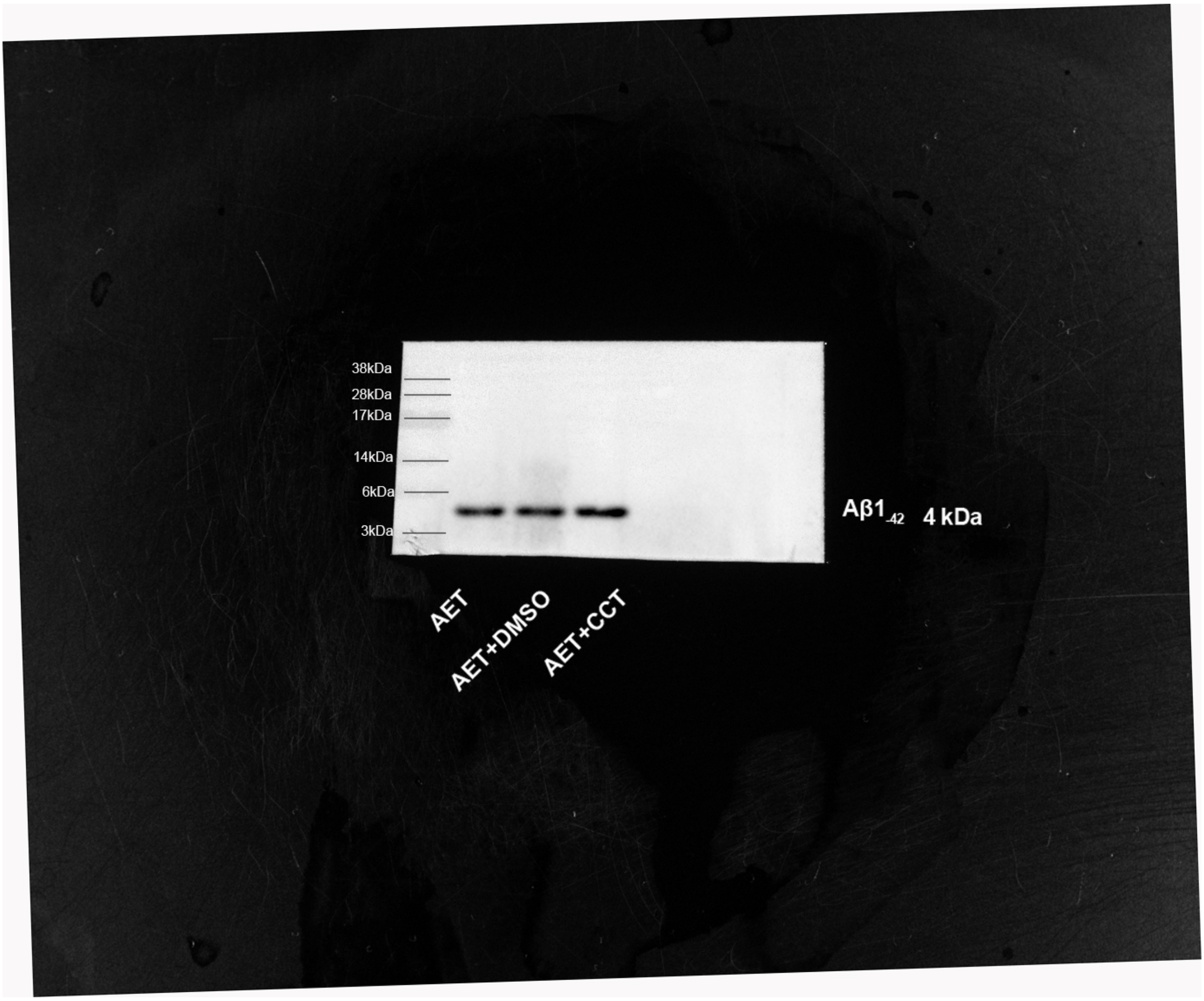

Full unedited gel/blot for Figure 6F

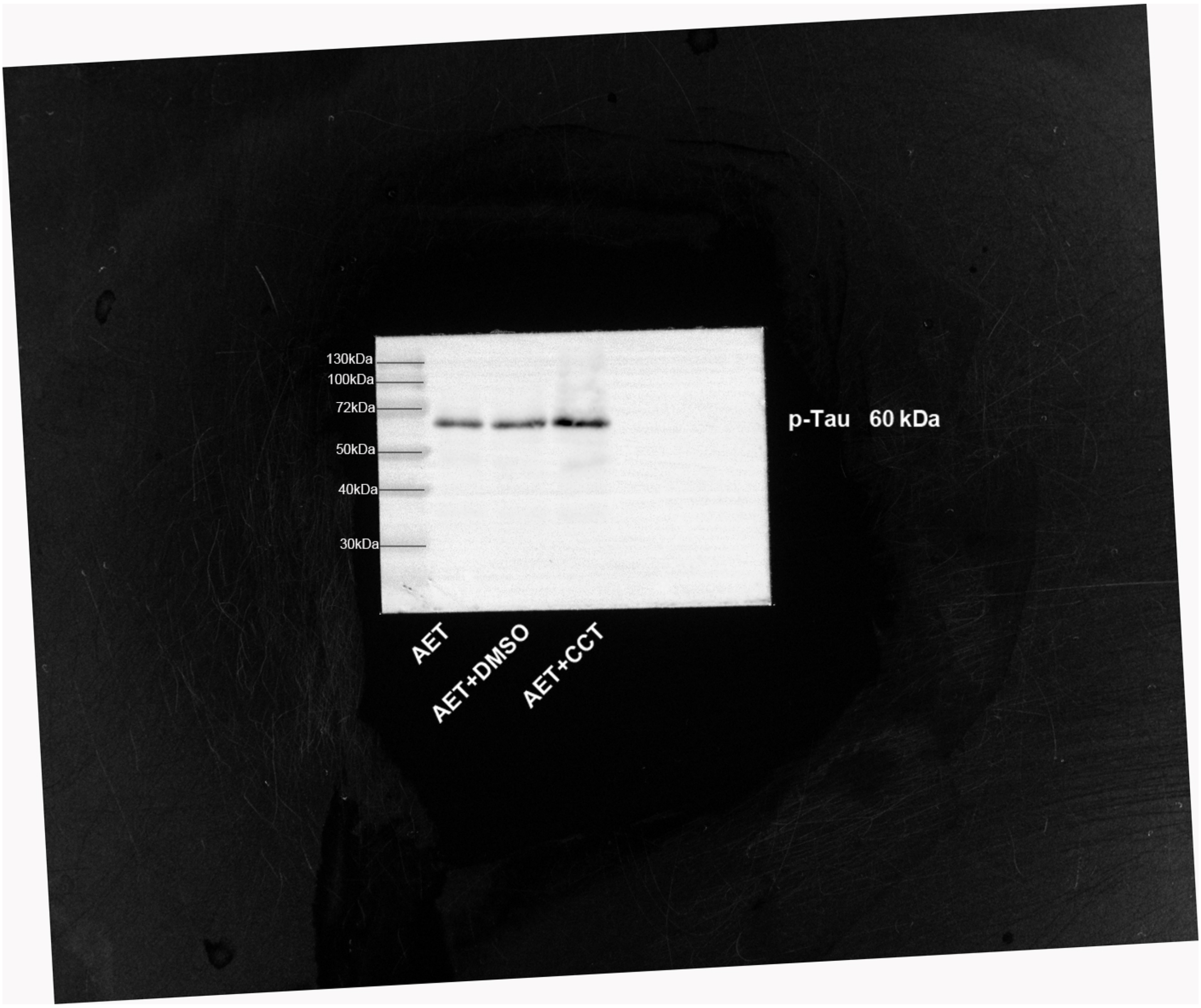

Full unedited gel/blot for Figure 6F

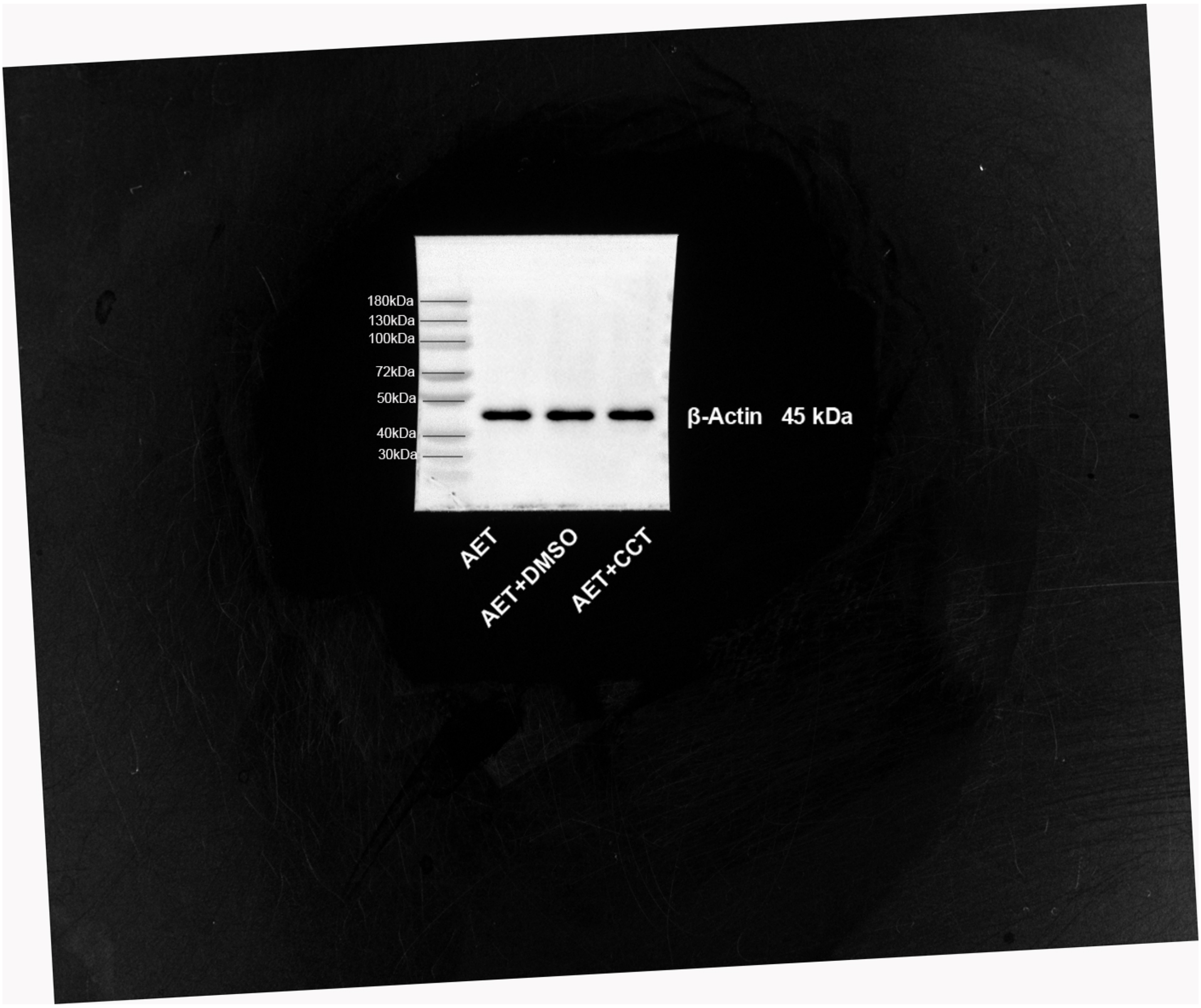

Supplement: Supplementary file 1 — Data S1: cns70620‐sup‐0001‐Supinfo1.pdf. [file CNS-31-e70620-s001.pdf]
